# Assessment of research waste part 1: an exemplar from examining study design, surrogate and clinical endpoints in studies of calcium intake and vitamin D supplementation

**DOI:** 10.1186/s12874-018-0556-0

**Published:** 2018-10-10

**Authors:** Mark J. Bolland, Alison Avenell, Andrew Grey

**Affiliations:** 1 Bone and Joint Research Group, Department of Medicine, University of Auckland, Private Bag 92 019, Auckland, 1142 New Zealand; 2 Health Services Research Unit, University of Aberdeen, Foresterhill, Aberdeen, AB25 2ZD Scotland

**Keywords:** Calcium intake, Vitamin D, Research waste, Observational studies, Randomized controlled trials, Surrogate endpoints, Bone density, Fracture

## Abstract

**Background:**

Research waste is estimated to be very common, but assessments of its prevalence and scope are rare. As an example, we assessed research waste in clinical research on calcium intake (assessing study design and endpoint type) and vitamin D supplementation (assessing endpoint type).

**Methods:**

We examined 404 randomised controlled trials (RCTs) and observational studies of calcium intake (diet or supplements) and bone mineral density (BMD) or fracture, and 547 RCTs of vitamin D supplements, and assessed the proportion of studies that used surrogate or clinical endpoints. For studies with BMD or fracture as an endpoint, we estimated when the ‘tipping’ point occurred indicating the need for RCTs with fracture as an endpoint (based on cumulative meta-analyses of BMD RCTs, and chronological review of observational studies), and whether each study published at least 5y after the tipping point was novel, added new clinical knowledge or was research waste.

**Results:**

Observational studies of calcium intake and BMD or fracture outnumbered RCTs by 3.3–4.5 times. For both calcium intake and vitamin D supplements, studies using surrogate endpoints outnumbered studies using clinical endpoints by 1.6–3 times. Of 41 RCT publications of calcium intake and BMD or fracture published at least 5y after the tipping point in 1994, we considered that 19 (46%) lacked novelty, another 13 (32%) added no new clinical knowledge, and 30 (73%) were research waste. Of 204 observational study publications of calcium intake and BMD or fracture, 197 (96%) lacked novelty, another 5 (2%) added no new clinical knowledge, and 202 (99%) were research waste. Of 39 RCTs of vitamin D supplementation and BMD or fracture published at least 5y after the tipping point in 1999, 14 (36%) lacked novelty, another 13 (33%) added no new clinical knowledge, and 27 (69%) were research waste.

**Conclusions:**

A high proportion of studies of calcium intake since 2000 (95%) and trials of vitamin D supplements since 2005 (69%) on BMD or fracture represent research waste.

**Electronic supplementary material:**

The online version of this article (10.1186/s12874-018-0556-0) contains supplementary material, which is available to authorized users.

## Background

Redundant research represents a large cost to society and misuses participants’ resources and altruism. Chalmers and Glasziou estimated that more than 85% of research might be wasteful because the wrong research questions are studied or because of poorly designed studies, inaccessible results, and biased reporting [1]. Very few studies have systematically examined research topics for evidence of waste, or established the methodology for doing so. In these companion reports [2], we assessed research waste in a single field - calcium and vitamin D research.

Calcium intake (through diet or supplements) and vitamin D supplementation have been very extensively researched as interventions for older adults for over 30 years. While undertaking recent systematic reviews [3–8], we noticed that many studies in these fields seemed to be designed to replicate existing knowledge rather than to determine new clinically relevant findings. The large body of published research in these fields allows an assessment of one important aspect of research waste, the quantity of research conducted that was unnecessary because existing knowledge was extensive. In this first report, we use existing databases compiled from our recent meta-analyses, which collated all studies of increased calcium intake and all randomised controlled trials (RCTs) of vitamin D supplements. We assess whether each study with an endpoint of bone mineral density (BMD) or fracture constitutes research waste, i.e. an unnecessary duplication without addressing shortcomings in previous research [9]. We focus on study design (observational versus RCT) in calcium intake studies and type of endpoint (surrogate versus clinical) in vitamin D supplementation RCTs.

## Methods

### Literature search and study selection

#### Calcium intake studies

The literature search has previously been published and is described in full in the Additional file 1: Supplementary Methods, Table S1, Figure S1 [7, 8]. Briefly, we identified 404 RCTs and observational studies (cohort, case-control, or cross-sectional studies) published before September 2014 of calcium, milk, or dairy intake, or calcium supplements with fracture or BMD as outcomes in participants > 50 years who had no major systemic illnesses other than osteoporosis. As a secondary analysis, we also assessed studies with cardiovascular and cancer endpoints.

#### Vitamin D supplementation RCTs

The search is described in full in the Additional file 1: Supplementary Methods, Tables S2-S3, Figure S2. Briefly, we identified 547 RCTs of vitamin D supplements in adults (>18y) published before December 2015 and classified them by the clinical relevance of their endpoints. We categorised each RCT according to whether clinical or surrogate endpoints were reported in the Abstract, using the Institute of Medicine’s definition of surrogate outcomes as “biomarker[s] intended to substitute for a clinical endpoint [and] expected to predict clinical benefit (or harm …) based on epidemiologic, therapeutic, pathophysiologic, or other scientific evidence” [10]. If there were multiple publications or endpoints, we included the study report with the most relevant clinical endpoint, or if there were no clinical endpoints, the study report with the most clinically relevant surrogate endpoint (Table 1).

**Table 1:** Classification of endpoints in 547 randomised controlled trials of vitamin D supplementation

Endpoint^a^	Number of RCTs
Clinical endpoints	
Fracture	18
Falls	17
Respiratory (eg asthma, COPD, URTI)	14
Musculoskeletal symptoms/Pain	11
Pregnancy outcomes	9
Tuberculosis	8
Multiple Sclerosis	7
Mood	6
SLE/Rheumatoid Arthritis	6
Other	41
Surrogate endpoints	
Bone mineral density	57
25OHD only	49
HbA1c or measures of glycaemia	42
Blood pressure	38
Basic biochemistry	37
Bone turnover markers	31
25OHD, vitamin D metabolites and/or PTH only	24
Muscle Strength	16
Body weight	15
Physical performance tests	14
Vascular properties	7
Hepatitis C viral load	5
Lung function tests	4
Lipids	4
Other laboratory tests/assays	51
Other endpoints	16

*Abbreviations*: *COPD* chronic obstructive pulmonary disease, *URTI* upper respiratory tract infection, *SLE* Systemic lupus erythematosus, *25OHD* 25 hydroxyvitamin D, *PTH* parathyroid hormone

^a^many trials reported more than 1 endpoint in the abstract. The most clinically relevant endpoint was used for each study

### Determination of research waste

For observational studies of calcium intake, we examined the results of publications chronologically and by consensus (MB, AG) determined the ‘tipping’ point - the time at which the hypothesis that calcium intake was associated with BMD or fracture had been generated and warranted testing in RCTs. For RCTs of calcium intake or vitamin D supplements, we conducted cumulative meta-analyses for BMD, a surrogate endpoint for the clinical endpoint of fracture. These meta-analyses were based on previous systematic reviews of calcium intake (with or without vitamin D supplements) and BMD [8] and vitamin D supplements (used as monotherapy, such that treatment groups only differed by vitamin D use) on BMD [6]. We then determined by consensus (MB, AG) if a ‘tipping’ point occurred: that is, the time at which the treatment effect was established and RCTs were required to investigate efficacy for the clinically meaningful outcome of fracture. For each category of research, we allowed a 5 year period after the tipping point, for publication of research in progress and dissemination and uptake of existing knowledge.

Each subsequent publication was independently reviewed by two authors (MB, AG). Firstly, we determined whether its study design was novel, and secondly whether it added to existing clinical knowledge and then, taking into account both these factors in a stepwise fashion, whether it represented research waste. These assessments were not based on study results. We considered a study novel when the population group, intervention, or dose did not overlap with existing publications, or there were potentially important novel features in the study design. Studies not considered novel were classified as research waste. We considered a study added to existing clinical knowledge if, by its design, it could produce results that might potentially modify the existing conclusion that RCTs with fracture as an endpoint were required. Studies that did not add to existing knowledge were considered research waste. Thus, a study could be classified as novel (e.g. a previously unstudied population) but if it could not add to existing knowledge, it was classified as research waste. Assessments were performed independently and any disagreements resolved by discussion. Agreement between assessors for novelty was 69–92%, for addition of knowledge 80–98%, and for waste 76–98%.

### Statistics

Cumulative meta-analyses were performed using random effects meta-analyses based on previously published data [6, 8] using Comprehensive Meta-Analysis (Version 2, Biostat, Englewood New Jersey, USA). *P*-values < 0.05 were considered statistically significant.

## Results

### Calcium intake studies

#### Study design

**Fig. 1:**
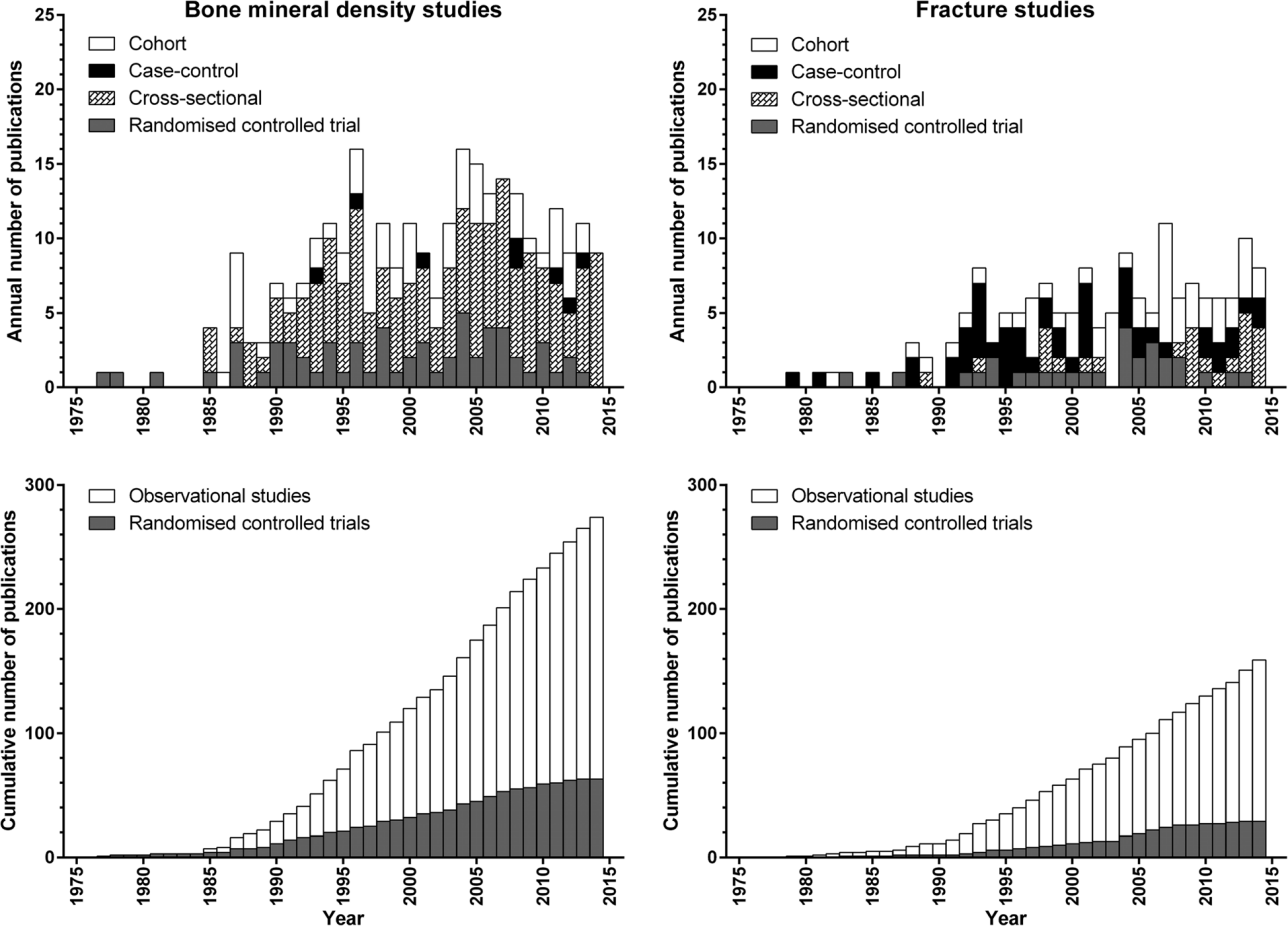
Number of publications over time of calcium intake with bone mineral density (left panels) or fracture (right panels) as endpoints by study design over time

Figure 1 shows the number of publications of calcium intake with BMD or fracture as an endpoint over time, by endpoint and study design. Observational studies outnumber RCTs by 3.3–4.5 times, and studies with BMD as an endpoint outnumber studies with fracture as an endpoint by 1.6–2.2 times. By 2014, the number of RCTs with either endpoint had plateaued, while the number of observational studies continued to steadily increase. For example, between 2009 and 13, there were 43 publications from observational studies and 8 from RCTs with BMD as an endpoint, and 31 publications from observational studies and 3 from RCTs with fracture as an endpoint.

Table 2 shows the secondary analysis results with the number of publications of calcium intake with clinical non-skeletal endpoints by study design. Observational studies outnumber RCTs by 3.4–43 times. Despite the very large number of observational studies, only two RCTs had a primary non-skeletal endpoint.

**Table 2:** Publications of calcium intake with non-skeletal endpoints by study design

Endpoint	Observational studies				RCT^a^
	Cross-sectional	Case-control	Cohort	Total	
IHD/MI	1	4	27	31	9^b^
Stroke	1	2	25	27	8^b^
Cancer (any site)	0	188	112	300	7^c^
Colorectal cancer	0	49	45	94	3^c^
Breast cancer	0	25	25	50	3^c^
Prostate cancer	0	34	32	66	2^c^
Colorectal adenoma	3	14	8	25	2

*Abbreviation*: *IHD/MI* ischaemic heart disease or myocardial infarction, *RCT* randomised controlled trial

^a^Two RCTs had colorectal adenoma as the primary endpoint. All other RCTs reported data as secondary endpoints

^b^Unpublished data for myocardial infarction and stroke from an additional 11 RCTs published in meta-analyses [25, 26]

^c^Unpublished data for cancer published in a meta-analysis [27] from an additional 6 RCTs for total cancer and colorectal cancer, 4 RCTs for breast cancer, and 2 RCTs for prostate cancer

#### Determination of tipping point

**Fig. 2:**
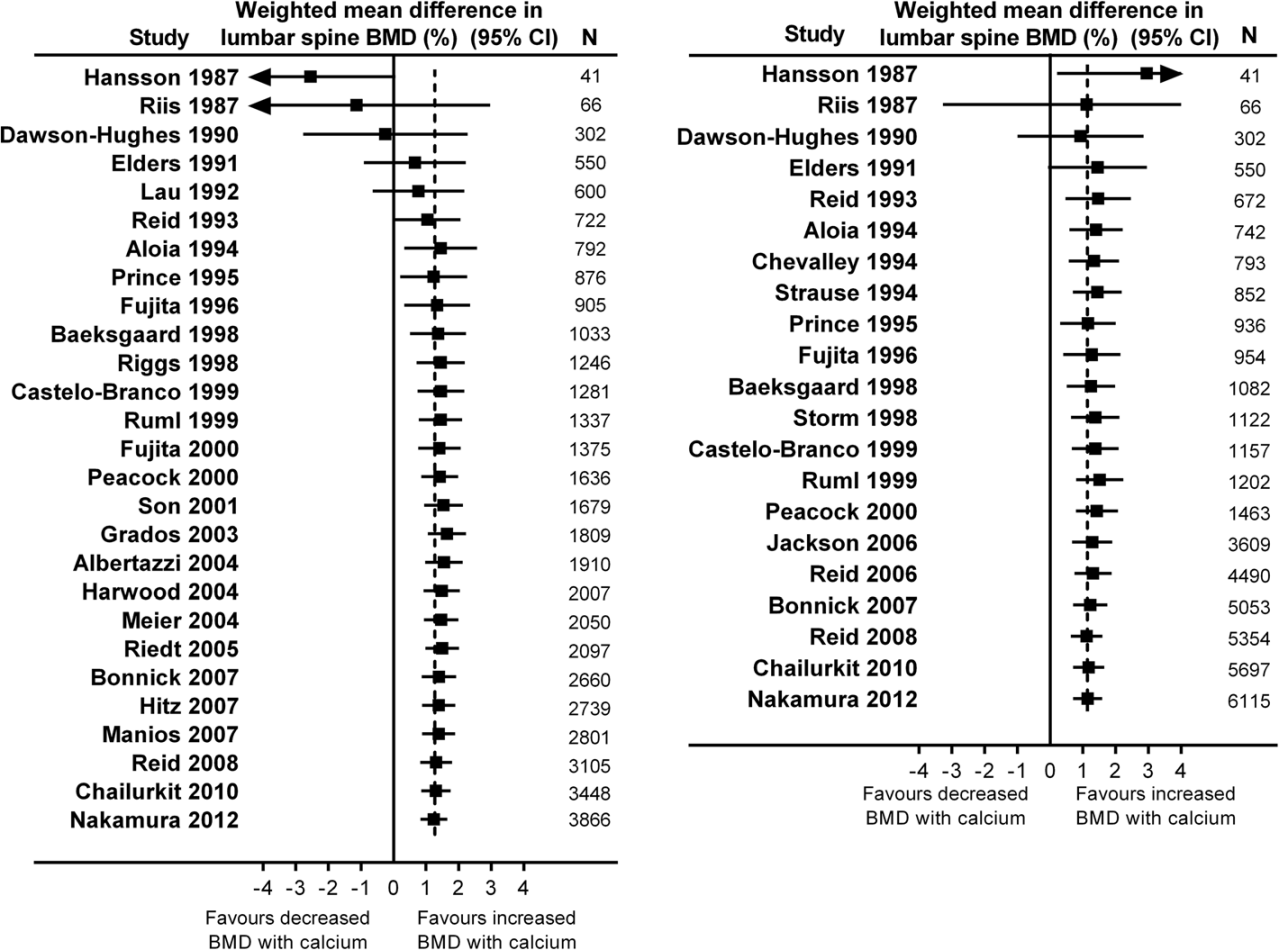
Cumulative meta-analyses of the effect of increased calcium intake (through supplements or dietary sources) with or without vitamin D supplements on lumbar spine bone mineral density (BMD) in randomised controlled trials at 1 year (left panel) and 2 years (right panel)

**Fig. 3:**
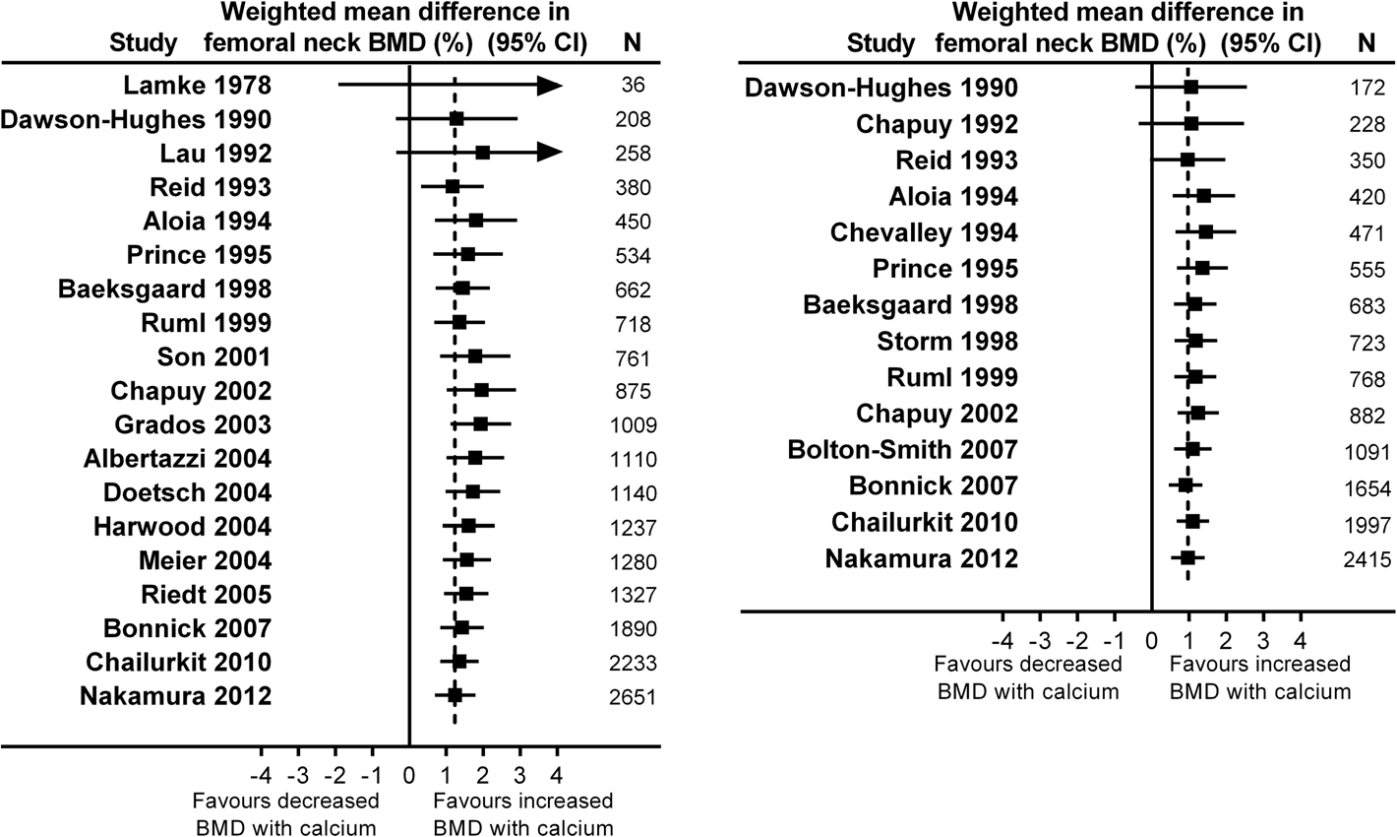
Cumulative meta-analyses of the effect of increased calcium intake (through supplements or dietary sources) with or without vitamin D supplements on femoral neck bone mineral density (BMD) in randomised controlled trials at 1 year (left panel) and 2 years (right panel)

We performed cumulative meta-analyses of the effect of increasing calcium intake on lumbar spine (Fig. 2) or femoral neck BMD (Fig. 3) in RCTs at 1y and 2y. By the end of 1994, the meta-analyses show statistically significant increases in BMD at the lumbar spine of 1.4% (95% CI 0.3, 2.6) at 1y from 7 RCTs involving 792 participants, and 1.4% (95% CI 0.7, 2.2) at 2y from 8 RCTs involving 852 participants. Likewise, by this time the pooled effect was 1.4–1.8% at the femoral neck at 1-2y from 5 RCTs involving 450–471 participants. The pooled effect sizes change little with the addition of data from later RCTs. By the end of 1994, RCTs had been conducted in most clinically relevant populations, including early and late post-menopausal women, men, people with recent fractures, different ethnic populations, and cohorts with low calcium intake. In addition, different doses of calcium supplements, different supplements, and milk products had all been evaluated in RCTs (Additional file 1: Table S4).

The first cohort study with more than 100 fractures and 10,000 participants was published in 1991, and by the end of 1994 there were 6 publications from cohort studies involving > 28,000 participants with > 600 hip fractures. By this time, 24 observational studies (6 cohort, 18 case-control, and 2 cross-sectional studies) had reported on the relationship between dietary calcium, dairy or milk intake or calcium supplements and fracture in cohorts with a wide variety of clinical characteristics.

We therefore defined 1994 as the tipping point for calcium intake and fractures. By this time, observational studies had reported that calcium intake might be associated with fracture risk, and RCTs had confirmed that increasing calcium intake increased the surrogate endpoint of BMD. Thus, further RCTs with BMD as an endpoint or observational studies with either BMD or fracture endpoints were unnecessary unless there was a novel hypothesis to be tested. Allowing 5y for dissemination of this information, we considered whether publications from 2000 onwards represented research waste.

#### Estimating research waste

Of 41 RCTs publications from 2000 onwards (Additional file 1: Table S4), 19 reported fracture data. Seven RCTs had a primary endpoint of fracture and were considered novel and not waste. One publication was a novel exploratory analysis of a previous RCT which had fracture as the primary endpoint, but did not add new clinical knowledge and we considered it research waste. Three RCTs with other novel primary endpoints reported fracture data as a secondary endpoint, but none added new clinical knowledge because of their small size and few fracture events. We considered 1 of the 3 publications to be research waste because it did not report the primary endpoint of the study, instead being a secondary publication that only reported fracture data. The major bone-related focus of the remaining 30 RCTs was BMD, either as the primary (*n* = 23) or secondary (*n* = 7) endpoint. Eight of these 30 RCTs reported fracture data as a secondary endpoint. Of these 30 RCTs, we considered 11 novel (8 novel study design, 2 novel population groups, 1 novel dose/population), but only 2 of these 11 RCTs added new clinical knowledge, and we considered that 28 of the 30 RCTs were research waste. Thus, of the 41 RCT publications at least 5y after the tipping point, 19 (46%) lacked novelty, another 13 (32%) did not add new clinical knowledge, and overall we considered 30 (73%) of these RCT publications were research waste. Twenty-four of the 30 reports were primary publications of RCTs (*n* = 4537 participants).

Of 204 observational study publications from 2000 onwards, 133 reported data on calcium intake and BMD, and 82 on fracture (Additional file 1: Table S5). In 51, 26, and 10 publications respectively, a primary purpose was to examine the relationship between calcium intake and BMD, or fracture, or both BMD and fracture. In the other 82, 56, and 1 publications respectively, examining calcium intake was a secondary hypothesis, with the primary purpose to either investigate the determinants of BMD, or fracture risk, or both BMD and fracture risk, or to examine the relationship between a specific factor and these endpoints. We considered that 4/133 publications reporting BMD relationships and 3/82 reporting fracture relationships were novel. In 1 publication considered a novel study design, the relationship between calcium intake and BMD was the primary focus. In the other 6 publications, the relationship between calcium intake and BMD or fracture was only a secondary focus: 2 examined determinants of fracture and 2 determinants of BMD in previously unreported clinical situations, and 2 developed predictive models for BMD or fracture. We considered that 2 of these 7 papers added new clinical knowledge, whereas the other 5 were research waste. Thus, of the 204 observational study publications from 2000 onwards, 197 (96%) lacked novelty, another 5 (2%) did not add new clinical knowledge, and overall we considered 202 (99%) were research waste. Of the 67 publications with a primary focus on calcium intake and BMD or fracture, only 1 was novel, and all were considered research waste.

### Vitamin D supplementation randomised controlled trials

#### Study classification by endpoint

**Fig. 4:**
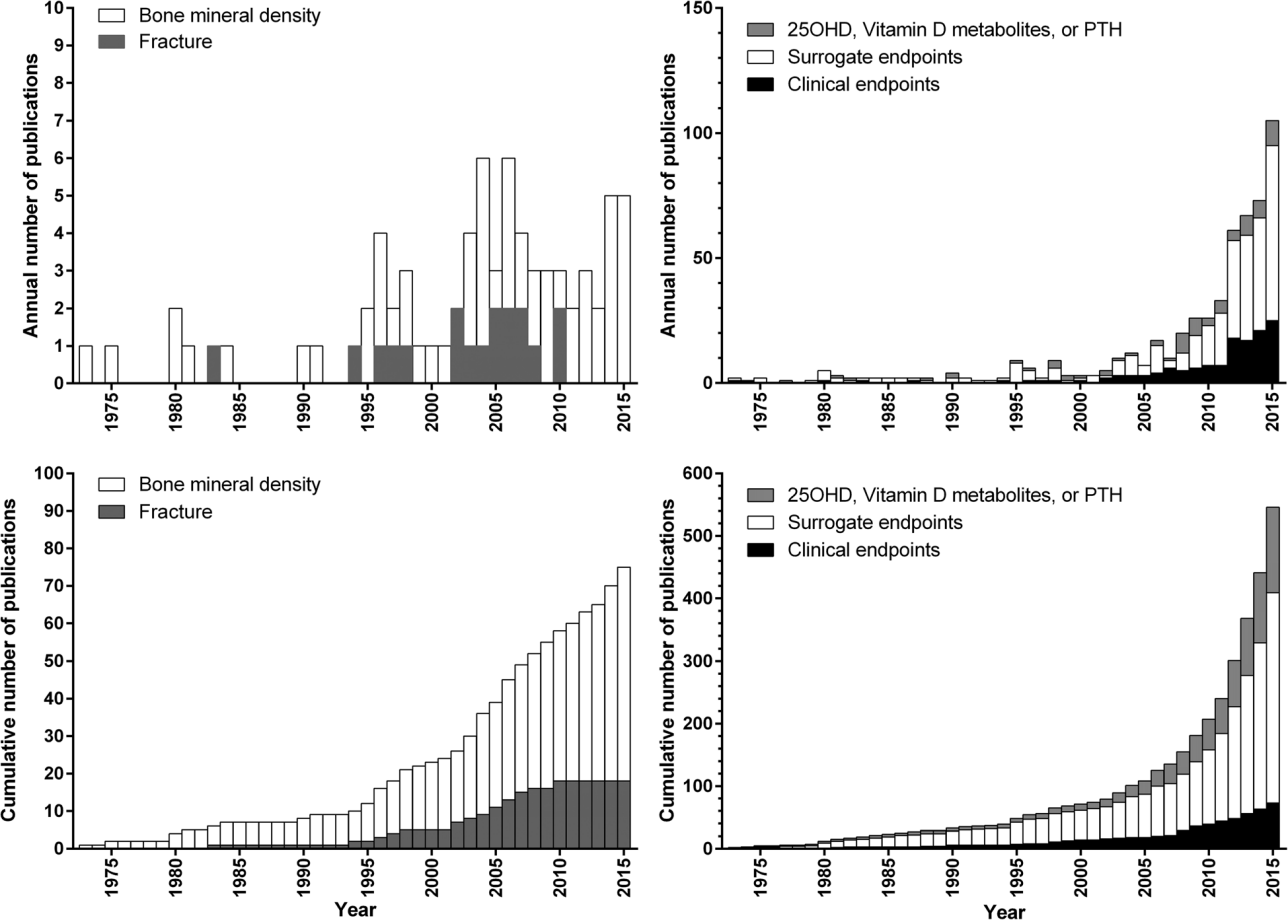
Numbers of publications of randomised clinical trials of vitamin D supplements over time classified according to the clinical or most clinically relevant surrogate endpoint reported in the Abstract. Left panel: trials with bone mineral density or fracture. Right panel: all trials classified according to clinical or surrogate endpoints. 25OHD- 25-hydroxyvitamin D

Figure 4 shows the number of RCTs with clinical endpoints in the abstract (*n* = 137) compared with RCTs with surrogate but no clinical endpoints in the abstract (*n* = 410). The RCTs with surrogate endpoints are further subdivided into those that only report concentrations of 25-hydroxyvitamin D, other vitamin D metabolites, and/or parathyroid hormone in the abstract (*n* = 73) and those that report other endpoints (*n* = 337). There has been a steady increase in the number of RCTs published over time, particularly since 2005. Since 2012, there have been more than 50 RCTs published each year. RCTs with surrogate endpoints outnumber RCTs with clinical endpoints by 3 times. In RCTs with skeletal endpoints, RCTs of the surrogate endpoint, BMD (*n* = 57), outnumber RCTs with the clinical endpoint of fracture (*n* = 18) by 3.2 times.

#### Determination of tipping point

**Fig. 5:**
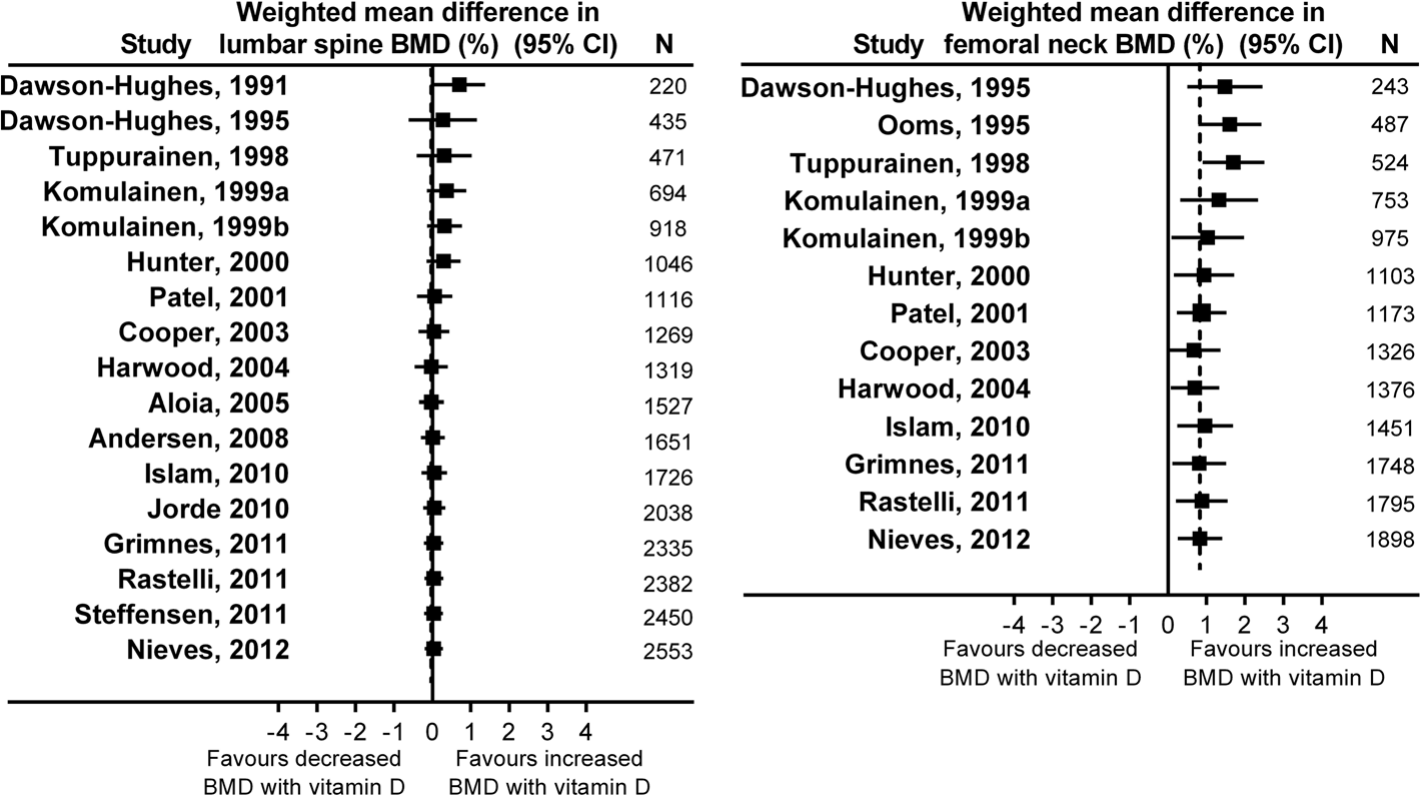
Cumulative meta-analyses of the effect of vitamin D supplements (used as monotherapy, such that treatment groups only differed by vitamin D use) on lumbar spine (left panel) and femoral neck bone mineral density (BMD) (right panel) in randomised controlled trials at 1–2 years

Figure 5 shows cumulative meta-analyses of the effect of vitamin D supplements at 1-2y on lumbar spine and femoral neck BMD in RCTs. By the end of 1999, the meta-analyses show stable effect sizes for BMD at each site from 4 RCTs involving > 900 participants. By this time, vitamin D supplements had no effect on lumbar spine BMD (*n* = 918, RR 0.3%, 95% CI -0.1, 0.8), but increased femoral neck BMD by 1.0% (95% CI 0.1, 2.0, *n* = 975). The pooled effect sizes change little with the addition of data from later RCTs. We therefore defined 1999 as the tipping point and, allowing 5y for dissemination of this information, we considered whether publications from 2005 onwards represented research waste.

#### Estimating research waste

Thirty-nine RCTs of vitamin D supplements with the most clinically relevant endpoint of BMD or fracture were published from 2005 onwards (Additional file 1: Table S6). In 9, the primary endpoint was fracture and all were considered novel and not waste. Of the remaining 30 RCTs that reported BMD data, we considered that 16 were novel because they included novel designs (*n* = 6), or populations (*n* = 10), but only 3 of these 16 studies added new clinical knowledge, and we considered that 27 of the 30 RCTs were research waste. Thus, of the 39 RCTs published at least 5y after the tipping point was reached, 14 (36%) were not novel, another 13 (33%) did not add new clinical knowledge, and overall we considered 27 (69%) of these RCTs (*n* = 3264 participants) were research waste.

## Discussion

These results, obtained in a specific field of research, support the view of Chalmers and Glasziou that a very high proportion of published research is wasteful. For both calcium intake and vitamin D supplementation, both observational studies and RCTs with surrogate endpoints were far more prevalent than RCTs with clinical endpoints. Thus, observational studies of calcium intake outnumbered RCTs by at least 3 times, and for both calcium intake and vitamin D supplementation, studies using surrogate endpoints outnumbered studies using clinical endpoints by 1.6–3 times. For the skeletal endpoints of BMD and fracture, these imbalances persisted long after the tipping point when the hypothesis that calcium intake or vitamin D supplementation might alter fracture risk had been addressed and answered. Of RCTs of calcium intake or vitamin D supplementation and fracture or BMD published at least 5y after the tipping point, 36–46% were not novel, testing hypotheses about effects on BMD that had previously been established. Of those considered novel, 60–80% added no new clinical knowledge, and amounted to a third of the published RCTs. Almost all observational studies of calcium intake and BMD or fracture published 5y after the tipping point were not novel. Thus, we considered that since 2000, 73% of RCTs, 98% of observational studies, and 95% of clinical research on calcium intake and BMD or fracture was wasteful and that since 2005, 69% of RCTs of vitamin D supplements and BMD or fracture were research waste. In research on non-skeletal endpoints, the marked imbalances between observational studies and RCTs, and between RCTs with surrogate and clinical endpoints suggest that waste in researching calcium and vitamin D is not restricted to bone health.

In estimating that 85% of research represents waste [1], Chalmers and Glasziou focused on four aspects - the choice of research questions, quality of research, unpublished results, and the quality of research reports. Our findings are relevant to the first and second aspects. Chalmers and Glasziou estimated that 50% of studies are wasteful because they are unnecessary, poorly designed without reference to a systematic review, and/or have major methodological weaknesses [1]. Our findings suggest that this may be a considerable underestimate, at least in some research areas. Studies with weaker designs (observational studies or RCTs with surrogate endpoints) were far more prevalent than RCTs with clinical endpoints, but we considered almost all (90–98%) such studies published over a 15–20 year period to represent research waste. If our findings apply to other fields, the overwhelming majority of RCTs and observational studies published in mature research areas may be wasteful.

A strength of our study is the extensive systematic literature searches and categorisation of large, complete sets of observational studies and RCTs of calcium intake and skeletal health, and RCTs of vitamin D supplements published over a long period of time. We are not aware of previous attempts to categorise or quantify research waste, but our methods are very labour and time intensive, and require adequate resourcing if they were to be more widely utilised. Limitations include that only two research areas were studied and therefore the results might not apply to other fields, and that we estimated the scope but not cost of the waste. We also considered both study design and study endpoints, which we think makes any conclusions more compelling than if the research areas or the study design and endpoints were assessed separately. Assessing studies as novel, contributing to clinical knowledge, or representing research waste requires subjective decisions. In general, however, agreement between the independent assessments was high. Agreement was lowest for novelty because many studies were of highly selected cohorts not previously studied. Deciding whether small differences from previous cohorts (such as ethnicity, gender or age) are sufficiently unique to be considered novel is difficult. Replication of previous studies is essential, and while disagreements may occur as to whether a study represents replication or redundancy, the number of observational studies and RCTs with surrogate endpoints extended well beyond the number needed for replication. Research can take a long time from inception to completion, and it is possible that some studies were initiated before the tipping point but not published until more than 5y after the tipping point, and were then assessed as not contributing to current knowledge. The literature search includes papers published until September 2014. As a methodological study, it was an extremely comprehensive, but very time-consuming attempt to identify all relevant clinical studies (observational and RCT) for a large number of outcomes. This search took almost 12 months to complete, and so it was not practical to update it subsequently. We are not aware of an RCT of calcium or vitamin D with fracture as the primary endpoint reported since 2014. Based on the data from 2011 to 2014, we would expect more than 75 observational studies to have been published since 2014, but < 10 RCTs of calcium and > 200 RCTs of vitamin D supplements. These recent publications are therefore unlikely to alter our conclusions related to research waste in this field.

An important issue raised by this research is how to assess research waste. Widely accepted definitions of waste are not yet available, and an element of subjectivity will always be required. To minimise the impact of subjectivity, we followed approaches used for high quality evidence-based systematic reviews: we explicitly stated the criteria for studies to be categorised as research waste, independently assessed studies, documented levels of agreement, resolved disagreements by consensus discussion, and reported our judgements for each study. However, other researchers might classify studies differently, and differences of opinion are common in similar circumstances. For example, Cochrane researchers recently reported that 45/156 risk of bias judgements differed between two groups of authors [11]. Differences in definitions and study classification between researchers might provide insight into this issue and lead to better definitions of research waste, and better approaches to its assessment.

A related issue is when the tipping point can be considered to have occurred. In the design phase of any RCT, a literature review should be standard practice. Thus, trialists should be aware of the most recent evidence before the study protocol is finalised, and therefore whether RCTs with meaningful clinical endpoints should be undertaken, or whether more observational studies or RCTs with surrogate endpoints are still required. Researchers may have different views on the exact date the tipping point occurred, but the cumulative meta-analyses and publication rates of observational studies highlight that it was many years ago that the hypothesis of fracture prevention for both calcium and vitamin D supplementation was formulated. The exact date of the tipping point, and whether 5 years is sufficient to allow dissemination of that information may be open to debate, but, in our example, would not change the conclusion that the majority of recent published research has not addressed the issue of fundamental clinical importance- whether calcium or vitamin D supplementation prevents fractures.

Research waste and how it can be reduced is increasingly being considered an important topic [9, 12–19]. The suggestions most relevant to our findings are the improvement of research priorities [9] and study design [15], in particular, performing systematic reviews to inform research proposals, and ensuring that study methods are optimum. Our finding that research waste continues unabated for long periods of time after a hypothesis has been generated or a research question has been answered suggests that most academics and funders have not so far corrected this problem. Publication of all RCTs is recommended, so if research has progressed to the stage of publication, it is difficult to argue that the primary RCT results should not be published. Considerable onus therefore necessarily falls upon assessments performed at the funding or ethical approval stage. These should be informed by a systematic review [9, 20], but that may not be straightforward. If systematic reviews exist, they often have discordant findings [21], and “living” systematic reviews updated with new evidence in real-time are not yet available [22]. Conducting a systematic review is time-consuming but necessary if one is not available or out-of-date. Finding relevant research is difficult because bibliographic database searches are non-specific, require screening of very many titles and abstracts, and frequently miss relevant studies. Involvement of information scientists and methodological expertise in the appraisal of funding applications might help to reduce research waste. This would require investment in appropriate resources but might improve the efficiency and accuracy of research funding.

We found that a very high proportion of observational research on calcium intake was wasteful. Many observational analyses are conducted opportunistically [23], and journal reviewers and editors could play important roles in declining manuscripts that examine established hypotheses that need, or have already been tested in, randomised studies, or where the findings are fragile and unlikely to be correct [24]. However, the large number of journals courting publications and academic incentives for publication quantity suggest that this strategy may be difficult to realise.

## Conclusion

We identified a very high proportion of research waste in the fields of calcium intake and vitamin D supplementation for bone health after the need for RCTs with meaningful clinical endpoints had been established. This waste was largely due to continued publication of observational studies and RCTs with surrogate endpoints unable to address the relevant clinical questions. Nearly all the observational studies and nearly half the RCTs were not novel and another third of RCTs added no new clinical knowledge. Thus, collectively, 95% of recent RCT or observational studies of calcium intake and BMD or fracture, and 69% of recent RCTs of vitamin D supplements and BMD or fracture were research waste. Strategies to reduce research waste should be devised and studied to determine their effectiveness.

## Additional file

Additional file 1: **Supplementary methods**. **Table S1**. Literature searches for calcium intake studies. **Table S2**. Searches of Pubmed for vitamin D randomised controlled trials undertaken in December 2015. **Table S3**. Thirty-eight systematic reviews of vitamin D supplements identified in Pubmed search. **Table S4**. Classification and characteristics of randomised controlled trials of increased calcium intake and bone mineral density or fracture. **Table S5**. Classification and characteristics of observational studies of calcium intake and bone mineral density or fracture. **Table S6**. Classification and characteristics of randomised controlled trials of vitamin D supplements and bone mineral density or fracture. **Figure S1**. Flow of studies for calcium intake. **Figure S2**. Flow of studies for randomised controlled trials of vitamin D supplements. (DOCX 922 kb)

## Abbreviations

BMD: bone mineral density
RCT: randomised controlled trial
