# Supplementary material for: Assessment of research waste part 1: an exemplar from examining study design, surrogate and clinical endpoints in studies of calcium intake and vitamin D supplementation
Source: BMC Med Res Methodol. 2018 Oct 10;18:103. doi: 10.1186/s12874-018-0556-0 (PMC6180413; doi:10.1186/s12874-018-0556-0)
Supplement: Supplementary file 1 — Supplementary methods. Table S1. Literature searches for calcium intake studies. Table S2. Searches of Pubmed for vitamin D randomised controlled trials undertaken in December 2015. Table S3. Thirty-eight systematic reviews of vitamin D supplements identified in Pubmed search. Table S4. Classification and characteristics of randomised controlled trials of increased calcium intake and bone mineral density or fracture. Table S5. Classification and characteristics of observational studies of calcium intake and bone mineral density or fracture. Table S6. Classification and characteristics of randomised controlled trials of vitamin D supplements and bone mineral density or fracture. Figure S1. Flow of studies for calcium intake. Figure S2. Flow of studies for randomised controlled trials of vitamin D supplements. (DOCX 922 kb) [file 12874_2018_556_MOESM1_ESM.docx]

**Additional file**

**Supplementary methods**

Literature search and study selection

*Calcium intake studies*

The literature search has previously been published in full (Table S1).^1,2^ Briefly, in July 2013, we searched Ovid Medline and Embase since inception for English language studies of calcium, milk, or dairy intake, or calcium supplements with or without vitamin D that reported on a broad range of skeletal and non-skeletal endpoints including fracture and BMD. We also identified 120 systematic reviews or meta-analyses on these topics and hand-searched these papers and other recent reviews on fracture risk for other relevant articles. We updated these results in September 2014 with a focused search (no language restrictions) of Pubmed and Embase for studies with fracture or BMD as an endpoint.

We included randomised controlled trials (RCTs), cohort, case-control, or cross-sectional studies with fracture or BMD as an outcome where participants were aged >50 years at baseline, or for cohort studies, where the majority of follow-up occurred in participants >50 years. We excluded studies where most participants had a major systemic pathology at baseline other than osteoporosis, such as renal failure or malignancy. We included studies of calcium supplements used in combination with other treatment provided that the other treatment was given to both arms. The flow of articles is shown in Figure S1. This dataset of 404 studies represents RCTs and observational studies published on calcium intake and BMD or fracture before September 2014.

As a secondary analysis, we also assessed studies with cardiovascular and cancer endpoints identified in our initial search in July 2013. We did not update these results with a focused search for studies with non-skeletal endpoints. Based on our experience with the searches for the endpoints of BMD and fracture, the initial search reliably identified RCTs but did not identify a number of relevant observational studies, often because calcium intake was not mentioned in the abstract, title, or keywords.

*Vitamin D RCTs*

In December 2015, we searched Pubmed for RCTs of vitamin D in adults (>18y) (Table S2) and for recent systematic reviews on clinical conditions and major surrogate endpoints that were the primary endpoints in identified RCTs (Tables S2, S3). We included trials with an untreated or placebo group, trials comparing higher with lower dose vitamin D, trials with or without calcium supplements, and trials with multiple interventions provided that 2 study arms differed only by the use of vitamin D. We included quasi-randomized trials but excluded trials where the method of allocation was sequential or unreported, trials where vitamin D was administered routinely post-thyroidectomy, and trials of hydroxylated vitamin D analogues. The flow of articles is shown in Figure S2.

We categorised each RCT according to whether clinical or surrogate endpoints were reported in the Abstract (or full-text where there was no Abstract), using the Institute of Medicine definition of surrogate outcomes as “biomarker[s] intended to substitute for a clinical endpoint [and] expected to predict clinical beneﬁt (or harm …) based on epidemiologic, therapeutic, pathophysiologic, or other scientiﬁc evidence”.^3^ One author (MB) extracted data and classified the RCTs, and a second author (AG) checked the results. Where multiple endpoints were reported, we recorded the most relevant clinical endpoint, and if there were no clinical endpoints, the most clinically relevant surrogate endpoint (Table 1). Where there were multiple publications from the same RCT, we included the study with the most relevant clinical endpoint or the most clinically relevant surrogate endpoint. This dataset of 547 RCTs represents all RCTs of vitamin D supplements published before December 2015, classified according to the clinical relevance of their endpoints.

**References**

1. Bolland MJ, Leung W, Tai V, Bastin S, Gamble GD, Grey A, et al. Calcium intake and risk of fracture: systematic review. BMJ 2015; 351:h4580.

2. Tai V, Leung W, Grey A, Reid IR, Bolland MJ. Calcium intake and bone mineral density: systematic review and meta-analysis. BMJ 2015; 351:h4183.

3. IOM (Institute of Medicine). Evaluation of Biomarkers and Surrogate Endpoints in Chronic Disease. Washington (DC): National Academies Press (US); 2010.

**Table S1: Literature searches for calcium intake studies**

Ovid Medline search July 2013

1. Randomized Controlled Trials as Topic/

2. randomized controlled trial.pt. or randomied controlled trial.mp. or Randomized Controlled Trial/

3. controlled clinical trial.pt. or Controlled Clinical Trial/

4. Random Allocation/

5. Double-Blind Method/

6. Single-Blind Method/

7. clinical trial.pt. or exp Clinical Trials/

8. multicenter study.pt. or multicenter study.tw.

9. or/1-8

10. (clinical adj trial$).tw.

11. ((singl$ or doubl$ or trebl$ or tripl$) adj (mask$ or blind$)).tw.

12. placebo$.tw. or Placebos/ or (control adj (arm or group)).tw.

13. (allocat$ adj2 random$).tw.

14. or/10-13

15. 9 or 14

16. case report.tw.

17. letter/

18. historical article/

19. or/16-18

20. 15 not 19

21. Epidemiologic studies/

22. (case control or case-control).tw. or exp case control studies/

23. cohort.tw. or exp Cohort Studies/

24. (Follow up or follow-up).tw. or Follow-Up Studies/

25. (observational adj (study or studies)).tw.

26. Longitudinal.tw. or Longitudinal Studies/

27. (prospective adj (study or studies)).tw. or Prospective Studies/

28. (retrospective adj (study or studies)).tw. or Retrospective Studies/

29. ((cross sectional or cross-sectional) adj (study or studies or analy$)).tw. or Cross-Sectional Studies/

30. ((cross-over or crossover) adj (study or studies)).tw. or Cross-Over Studies/

31. risk factor$.tw. or Risk factors/

32. exp Calcium, Dietary/

33. milk.tw. or Milk/

34. (dairy or cheese).tw. or dairy products/ or butter/ or cheese/ or ice cream/ or margarine/

35. yoghurt.tw. or cultured milk products/ or yoghurt/

36. Milk Substitutes/ or Soy Milk/

37. calcium.tw. or exp Calcium/

38. (calcium adj3 supplement$).mp.

39. Dietary Supplements/ and calcium.mp.

40. calcium carbonate$.mp. or exp Calcium Carbonate/

41. calcium citrate$.mp. or exp Calcium Citrate/

42. calcium phosphate$.mp. or exp Calcium Phosphates/

43. calcium gluconate$.mp. or exp Calcium Gluconate/

44. hydroxyapatite$.mp. or exp Hydroxyapatites/

45. fracture$.tw. or exp Fractures, Bone/

46. osteoporo$.tw. or Osteoporosis/ or Osteoporosis, Postmenopausal/

47. (bone density or BMD).tw. or Bone Density/

48. bone mineral density.tw.

49. (bone adj (mass or strength or loss or accret$ or remodel$ or resorp$)).tw.

50. (bone mineral content or BMC).tw.

51. (cardiovascular adj (disease$ or event$ or acute)).tw.

52. isch$ heart disease$.tw. or Myocardial Ischemia/

53. (acute coronary syndrome or ACS).tw. or Acute Coronary Syndrome/

54. unstable angina.tw. or Angina, Unstable/

55. coronary artery disease$.tw. or Coronary Artery Disease/

56. (myocardial infarct$ or MI or AMI).tw. or Myocardial Infarction/

57. (cerebrovascular adj (accident$ or disease$ or acute)).tw. or exp Cerebrovascular Accident/

58. stroke$.tw. or exp Stroke/

59. mortality.tw. or Mortality/ or Hospital Mortality/ or Mortality, Premature/

60. fatal outcome.tw. or Fatal Outcome/

61. death$.tw. or Death/ or Cause of Death/ or Death, Sudden, Cardiac/

62. cancer$.tw. or exp Neoplasms/dh, pc, et, di, ep

63. ((risk$ or occurrence$ or case$ or incidence) adj3 (cancer$ or neoplasm$ or malignan$ or adenocarcinom$ or carcinom$ or melanom$ or lymphom$ or leuk?emi$ or myelodysplas$ or myelom$ or sarcom$)).tw.

64. ((breast or prostate or colo$ or rect$) adj3 (neoplasm$ or cancer$ or tumo$ or malignan$ or carcinom$ or adenocarcinom$)).tw.

65. Breast Neoplasms/

66. Prostatic Neoplasms/

67. Colonic Neoplasms/ or Colorectal Neoplasms/

68. Adenomatous Polyps/ or Colonic Polyps/ or Intestinal Polyps/

69. ((colo$ or rect$) adj3 (adenoma$ or polyp$)).tw.

70. (weight adj2 (body or gain or increas$ or rise$ or rose or loss or reduc$ or fall or fell or change)).tw. or Weight Loss/

71. blood pressure.tw. or Blood Pressure/

72. Hypertension/ or hypertension.tw.

73. cholesterol.tw. or Cholesterol/ or Cholesterol, Dietary/

74. Cholesterol, VLDL/ or Cholesterol, LDL/ or Cholesterol,HDL/

75. ((serum or blood) adj calcium).tw.

76. ((kidney or renal) adj (calcul$ or stone$ or lithiasis)).tw. or Kidney Calculi/

77. ((gastrointestinal or GI) adj (discomfort or side-effect$ or side effect$)).tw.

78. ((abdominal or stomach) adj (pain or cramp$)).tw. or Abdominal Pain/

79. constipation.tw. or Constipation/

80. indigestion.tw. or Dyspepsia/

81. Diarrhea/ or diarrhea.tw.

82. Flatulence/ or bloating.tw.

83. or/20-31

84. or/32-44

85. or/45-82

86. and/83-85

87. limit 86 to english language

88. limit 87 to animal

89. limit 87 to human

90. 88 not 89

91. 87 not 90

92. *Dialysis/ or *hemodialysis/ or *peritoneal dialysis/

93. 91 not 92

94. limit 93 to (addresses or bibliography or biography or comment or congresses or consensus development conference or consensus development conference, nih or dictionary or directory or duplicate publication or editorial or in vitro or interview or lectures or letter or news or newspaper article or "review")

95. 93 not 94

96. limit 95 to ("all adult (19 plus years)" or "middle age (45 to 64 years)" or "middle aged (45 plus years)" or "all aged (65 and over)" or "aged (80 and over)")

Pubmed search September 2014

(dietary calcium OR calcium intake OR milk OR dairy OR calcium supplement* OR calcium supplement) AND (fracture OR bone density OR bone mineral)

**Table S2: Searches of Pubmed for vitamin D randomised controlled trials undertaken in December 2015**

| **Database** | **Search Terms** | **Citations** |
| --- | --- | --- |
| Pubmed | Vitamin D with clinical trials filter | 4018 |
| Pubmed | Within title: (“Vitamin D” or “cholecalciferol” or “colecalciferol” or “ergocalciferol” or “calciferol”) and (random* or “trial”) | 631 |
| Pubmed | Vitamin D, publication date after 1/1/2015 | 634 |
| Pubmed | Systematic reviews or Meta-analyses of randomized controlled trials of vitamin D with clinical endpoints. We included the latest 2 publications on each topic but included any review published in 2014-5 | 38 |

**Table S3: 38 Systematic reviews of vitamin D supplements identified in Pubmed search**

| **Subject** | **Reference** |
| --- | --- |
| Rheumatoid Arthritis, Systemic lupus erythematosus | Antico A, Tampoia M, Tozzoli R, Bizzaro N. Can supplementation with vitamin D reduce the risk or modify the course of autoimmune diseases? A systematic review of the literature. *Autoimmun Rev.* 2012;12(2):127-136. |
| Mood; Diabetes; Cancer; Multiple sclerosis; | Autier P, Boniol M, Pizot C, Mullie P. Vitamin D status and ill health: a systematic review. *Lancet Diabetes Endocrinol.* 2014;2(1):76-89. |
| Fracture; Mortality | Avenell A, Mak JC, O'Connell D. Vitamin D and vitamin D analogues for preventing fractures in post-menopausal women and older men. *Cochrane Database Syst Rev.* 2014;4:CD000227. |
| Muscle strength | Beaudart C, Buckinx F, Rabenda V, et al. The effects of vitamin D on skeletal muscle strength, muscle mass, and muscle power: a systematic review and meta-analysis of randomized controlled trials. *J Clin Endocrinol Metab.* 2014;99(11):4336-4345. |
| Respiratory tract infection | Bergman P, Lindh AU, Bjorkhem-Bergman L, Lindh JD. Vitamin D and Respiratory Tract Infections: A Systematic Review and Meta-Analysis of Randomized Controlled Trials. *PLoS One.* 2013;8(6):e65835. |
| Blood pressure | Beveridge LA, Struthers AD, Khan F, et al. Effect of Vitamin D Supplementation on Blood Pressure: A Systematic Review and Meta-analysis Incorporating Individual Patient Data. *JAMA Intern Med.* 2015;175(5):745-754. |
| Cancer | Bjelakovic G, Gluud LL, Nikolova D, et al. Vitamin D supplementation for prevention of cancer in adults. *Cochrane Database Syst Rev.* 2014;6:CD007469. |
| Mortality | Bjelakovic G, Gluud LL, Nikolova D, et al. Vitamin D supplementation for prevention of mortality in adults. *Cochrane Database Syst Rev.* 2014;1:CD007470. |
| Fracture | Bolland MJ, Grey A. A case study of discordant overlapping meta-analyses: vitamin d supplements and fracture. *PLoS One.* 2014;9(12):e115934. |
| Fracture, Cardiovascular events; Cancer, Mortality | Bolland MJ, Grey A, Gamble GD, Reid IR. The effect of vitamin D supplementation on skeletal, vascular, or cancer outcomes: a trial sequential meta-analysis. *Lancet Diabetes Endocrinol.* 2014;2(4):307-320. |
| Falls | Bolland MJ, Grey A, Gamble GD, Reid IR. Vitamin D supplementation and falls: a trial sequential meta-analysis. *Lancet Diabetes Endocrinol.* 2014;2(7):573-580. |
| Falls | Bolland MJ, Grey A, Reid IR. Differences in overlapping meta-analyses of vitamin d supplements and falls. *J Clin Endocrinol Metab.* 2014;99(11):4265-4272. |
| Colorectal adenoma | Carroll C, Cooper K, Papaioannou D, Hind D, Pilgrim H, Tappenden P. Supplemental calcium in the chemoprevention of colorectal cancer: a systematic review and meta-analysis. *Clin Ther.* 2010;32(5):789-803. |
| Weight | Chandler PD, Wang L, Zhang X, et al. Effect of vitamin D supplementation alone or with calcium on adiposity measures: a systematic review and meta-analysis of randomized controlled trials. *Nutr Rev.* 2015;73(9):577-593. |
| Mortality | Chowdhury R, Kunutsor S, Vitezova A, et al. Vitamin D and risk of cause specific death: systematic review and meta-analysis of observational cohort and randomised intervention studies. *BMJ.* 2014;348:g1903. |
| Depression | Gowda U, Mutowo MP, Smith BJ, Wluka AE, Renzaho AM. Vitamin D supplementation to reduce depression in adults: meta-analysis of randomized controlled trials. *Nutrition.* 2015;31(3):421-429. |
| Pregnancy | Harvey NC, Holroyd C, Ntani G, et al. Vitamin D supplementation in pregnancy: a systematic review. *Health Technol Assess.* 2014;18(45):1-190. |
| General health | Hoffmann MR, Senior PA, Mager DR. Vitamin D supplementation and health-related quality of life: a systematic review of the literature. *J Acad Nutr Diet.* 2015;115(3):406-418. |
| Glycaemia | Jamka M, Wozniewicz M, Jeszka J, Mardas M, Bogdanski P, Stelmach-Mardas M. The effect of vitamin D supplementation on insulin and glucose metabolism in overweight and obese individuals: systematic review with meta-analysis. *Sci Rep.* 2015;5:16142. |
| Cancer | Keum N, Giovannucci E. Vitamin D supplements and cancer incidence and mortality: a meta-analysis. *Br J Cancer.* 2014;111(5):976-980. |
| Depression | Li G, Mbuagbaw L, Samaan Z, et al. Efficacy of vitamin D supplementation in depression in adults: a systematic review. *J Clin Endocrinol Metab.* 2014;99(3):757-767. |
| Lipids, weight, blood pressure | Manousopoulou A, Al-Daghri NM, Garbis SD, Chrousos GP. Vitamin D and cardiovascular risk among adults with obesity: a systematic review and meta-analysis. *Eur J Clin Invest.* 2015;45(10):1113-1126. |
| Respiratory tract infection | Mao S, Huang S. Vitamin D supplementation and risk of respiratory tract infections: a meta-analysis of randomized controlled trials. *Scand J Infect Dis.* 2013;45(9):696-702. |
| Crohn’s disease | Nicholson I, Dalzell AM, El-Matary W. Vitamin D as a therapy for colitis: a systematic review. *J Crohns Colitis.* 2012;6(4):405-411. |
| Pregnancy | Perez-Lopez FR, Pasupuleti V, Mezones-Holguin E, et al. Effect of vitamin D supplementation during pregnancy on maternal and neonatal outcomes: a systematic review and meta-analysis of randomized controlled trials. *Fertil Steril.* 2015;103(5):1278-1288 e1274. |
| Multiple sclerosis | Pozuelo-Moyano B, Benito-Leon J, Mitchell AJ, Hernandez-Gallego J. A systematic review of randomized, double-blind, placebo-controlled trials examining the clinical efficacy of vitamin D in multiple sclerosis. *Neuroepidemiology.* 2013;40(3):147-153. |
| Bone density | Reid IR, Bolland MJ, Grey A. Effects of vitamin D supplements on bone mineral density: a systematic review and meta-analysis. *Lancet.* 2014;383(9912):146-155. |
| Systemic lupus erythematosus | Sakthiswary R, Raymond AA. The clinical significance of vitamin D in systemic lupus erythematosus: a systematic review. *PLoS One.* 2013;8(1):e55275. |
| Glycaemia | Sarathy H, Pramanik V, Kahn J, et al. The effects of short-term vitamin D supplementation on glucose metabolism in dialysis patients: a systematic review and meta-analysis. *Int Urol Nephrol.* 2015;47(3):537-549. |
| Glycaemia; Diabetes | Seida JC, Mitri J, Colmers IN, et al. Clinical review: Effect of vitamin D3 supplementation on improving glucose homeostasis and preventing diabetes: a systematic review and meta-analysis. *J Clin Endocrinol Metab.* 2014;99(10):3551-3560. |
| Mood | Shaffer JA, Edmondson D, Wasson LT, et al. Vitamin D supplementation for depressive symptoms: a systematic review and meta-analysis of randomized controlled trials. *Psychosom Med.* 2014;76(3):190-196. |
| Bone density | Silk LN, Greene DA, Baker MK. The Effect of Calcium or Calcium and Vitamin D Supplementation on Bone Mineral Density in Healthy Males: A Systematic Review and Meta-Analysis. *Int J Sport Nutr Exerc Metab.* 2015;25(5):510-524. |
| Endothelial function | Stojanovic M, Radenkovic M. Vitamin D versus placebo in improvement of endothelial dysfunction: a meta-analysis of randomized clinical trials. *Cardiovasc Ther.* 2015;33(3):145-154. |
| Tuberculosis | Sutaria N, Liu CT, Chen TC. Vitamin D Status, Receptor Gene Polymorphisms, and Supplementation on Tuberculosis: A Systematic Review of Case-Control Studies and Randomized Controlled Trials. *J Clin Transl Endocrinol.* 2014;1(4):151-160. |
| Bone density | Tai V, Leung W, Grey A, Reid IR, Bolland MJ. Calcium intake and bone mineral density: systematic review and meta-analysis. *BMJ.* 2015;351:h4183. |
| Mortality, Fracture, Falls | Theodoratou E, Tzoulaki I, Zgaga L, Ioannidis JP. Vitamin D and multiple health outcomes: umbrella review of systematic reviews and meta-analyses of observational studies and randomised trials. *BMJ.* 2014;348:g2035. |
| Muscle strength | Tomlinson PB, Joseph C, Angioi M. Effects of vitamin D supplementation on upper and lower body muscle strength levels in healthy individuals. A systematic review with meta-analysis. *J Sci Med Sport.* 2015;18(5):575-580. |
| Tuberculosis | Xia J, Shi L, Zhao L, Xu F. Impact of vitamin D supplementation on the outcome of tuberculosis treatment: a systematic review and meta-analysis of randomized controlled trials. *Chin Med J (Engl).* 2014;127(17):3127-3134. |

No systematic reviews of randomised controlled trials of vitamin D were identified for Asthma, Chronic obstructive pulmonary disease, Pain, Dermatitis, ICU admissions, Seizure, Raynauds, Gingivitis, Osteoarthritis, Migraine, Uraemia, Chillblains, Alzheimer’s disease, Parkinson’s disease, Leg ulcer, Bacterial vaginosis, Vascular fistula, Infertility, Vaginal atrophy, Chronic fatigue, and Hepatitis.

**Table S4: Classification and characteristics of randomised controlled trials of increased calcium intake and bone mineral density or fracture**

| **Study** | **Primary**  **endpoint** | **BMD**  **data** | **Fracture**  **data** | **Novel** | **Reason** | **Add new clinical**  **knowledge** | **Reason** | **Waste** |
| --- | --- | --- | --- | --- | --- | --- | --- | --- |
| Recker, 1977^1^ | BMB | Yes | No |  |  |  |  |  |
| Lamke, 1978^2^ | BMC | Yes | No |  |  |  |  |  |
| Smith, 1981^3^ | BMC | Yes | No |  |  |  |  |  |
| Inkovaara, 1983^4^ | Biochem | No | Yes |  |  |  |  |  |
| Recker, 1985^5^ | Ca balance | Yes | No |  |  |  |  |  |
| Hansson, 1987^6^ | BMC | Yes | Yes |  |  |  |  |  |
| Polley, 1987^7^ | BMD | Yes | No |  |  |  |  |  |
| Riis, 1987^8^ | BMD | Yes | No |  |  |  |  |  |
| Smith, 1989^9^ | BMC | Yes | No |  |  |  |  |  |
| Dawson-Hughes, 1990^10^ | BMD | Yes | No |  |  |  |  |  |
| Fujita, 1990^11^ | BMD | Yes | No |  |  |  |  |  |
| Orwoll, 1990^12^ | BMC | Yes | No |  |  |  |  |  |
| Elders, 1991^13^ | BMD | Yes | No |  |  |  |  |  |
| Nelson, 1991^14^ | BMD | Yes | No |  |  |  |  |  |
| Prince, 1991^15^ | BMD | Yes | No |  |  |  |  |  |
| Chapuy, 1992^16^ | Fracture | Yes | Yes |  |  |  |  |  |
| Lau, 1992^17^ | BMD | Yes | No |  |  |  |  |  |
| Reid, 1993^18^ | BMD | Yes | Yes |  |  |  |  |  |
| Aloia, 1994^19^ | TBC | Yes | No |  |  |  |  |  |
| Chapuy, 1994^20^ | Fracture | No | Yes |  |  |  |  |  |
| Chevalley, 1994^21^ | BMD | Yes | Yes |  |  |  |  |  |
| Strause, 1994^22^ | BMD | Yes | No |  |  |  |  |  |
| Prince, 1995^23^ | BMD | Yes | No |  |  |  |  |  |
| Fujita, 1996^24^ | BMD | Yes | No |  |  |  |  |  |
| Perez-Jaraiz, 1996^25^ | BMD | Yes | No |  |  |  |  |  |
| Recker, 1996^26^ | Fracture | Yes | Yes |  |  |  |  |  |
| Dawson-Hughes, 1997^27^ | BMD | Yes | Yes |  |  |  |  |  |
| Baeksgaard, 1998^28^ | BMD | Yes | No |  |  |  |  |  |
| Ricci, 1998^29^ | Weight loss | Yes | No |  |  |  |  |  |
| Riggs, 1998^30^ | BMD | Yes | Yes |  |  |  |  |  |
| Storm, 1998^31^ | BMD | Yes | No |  |  |  |  |  |
| Castelo-Branco, 1999^32^ | BMD | Yes | No |  |  |  |  |  |
| Ruml, 1999^33^ | BMD | Yes | Yes |  |  |  |  |  |
| Fujita, 2000^34^ | BMD | Yes | No | Yes | Novel design | No | Confirmed | Yes |
| Peacock, 2000^35^ | BMD | Yes | Yes | Yes | Novel design | No | Confirmed | Yes |
| Cleghorn, 2001^36^ | BMD | Yes | No | No | Confirmed | No | Confirmed | Yes |
| Lau, 2001^37^ | BMD | Yes | Yes | No | Confirmed | No | Confirmed | Yes |
| Son, 2001^38^ | BMD | Yes | No | No | Confirmed | No | Confirmed | Yes |
| Chapuy, 2002^39^ | Biochem | Yes | Yes | No | Confirmed | No | Confirmed | Yes |
| Chee, 2003^40^ | BMD | Yes | No | No | Confirmed | No | Confirmed | Yes |
| Grados, 2003^41^ | Biochem | Yes | No | Yes | Novel population | No | Confirmed | Yes |
| Albertazzi, 2004^42^ | BMD | Yes | No | No | Confirmed | No | Confirmed | Yes |
| Avenell, 2004^43^ | Compliance | No | Yes | Yes | Secondary | No | Size | No |
| Doetsch, 2004^44^ | Callus | Yes | No | Yes | Secondary | Yes | Novel design | No |
| Fujita, 2004^45^ | BMD | Yes | Yes | No | Size | No | Size | Yes |
| Harwood, 2004^46^ | Biochem | Yes | Yes | No | Confirmed | No | Confirmed | Yes |
| Larsen, 2004^47^ | Fracture | No | Yes | Yes | Fracture study | Yes | Fracture study | No |
| Meier, 2004^48^ | Biochem | Yes | No | No | Confirmed | No | Confirmed | Yes |
| Coiro, 2005^49^ | BMD | Yes | No | Yes | Novel design | No | Confirmed | Yes |
| Grant, 2005^50^ | Fracture | No | Yes | Yes | Fracture study | Yes | Fracture study | No |
| Porthouse, 2005^51^ | Fracture | No | Yes | Yes | Fracture study | Yes | Fracture study | No |
| Riedt, 2005^52^ | Biochem | No | No | No | Confirmed | No | Confirmed | Yes |
| Daly, 2006^53^ | BMD | Yes | No | Yes | Novel population | No | Confirmed | Yes |
| Jackson, 2006^54^ | Fracture | Yes | Yes | Yes | Fracture study | Yes | Fracture study | No |
| Prince, 2006^55^ | Fracture | Yes | Yes | Yes | Fracture study | Yes | Fracture study | No |
| Reid, 2006^56^ | Fracture | Yes | Yes | Yes | Fracture study | Yes | Fracture study | No |
| Bolton-Smith, 2007^57^ | BMD | Yes | Yes | Yes | Novel design | No | Confirmed | Yes |
| Bonnick, 2007^58^ | BMD | Yes | Yes | Yes | Novel design | Yes | Novel design | No |
| Hitz, 2007^59^ | BMD | Yes | No | No | Confirmed | No | Confirmed | Yes |
| Manios, 2007^60^ | Biochem | Yes | No | No | Confirmed | No | Confirmed | Yes |
| Bischoff-Ferrari, 2008^61^ | Col aden. | No | Yes | Yes | Secondary | No | Size | Yes |
| Reid, 2008^62^ | BMD | Yes | Yes | Yes | Novel dose/  population | No | Confirmed | Yes |
| Zhu, 2008^63^ | BMD | Yes | No | No | Confirmed | No | Confirmed | Yes |
| Kukuljan, 2009^64^ | BMD | Yes | No | Yes | Novel design | No | Confirmed | Yes |
| Chailurkit, 2010^65^ | BMD | Yes | No | No | Confirmed | No | Confirmed | Yes |
| Karkkainen, 2010^66^ | BMD | Yes | No | No | Confirmed | No | Confirmed | Yes |
| Moschonis, 2010^67^ | BMD | Yes | No | No | Confirmed | No | Confirmed | Yes |
| Salovaara, 2010^68^ | Fracture | No | Yes | Yes | Fracture study | Yes | Fracture study | No |
| Kukuljan, 2011^69^ | BMD | Yes | No | No | Confirmed | No | Confirmed | Yes |
| Gui, 2012^70^ | BMD | Yes | No | No | Confirmed | No | Confirmed | Yes |
| Nakamura, 2012^71^ | BMD | Yes | No | Yes | Novel design | No | Confirmed | Yes |
| Sambrook, 2012^72^ | Falls | No | Yes | Yes | Secondary | No | Size | No |
| Prentice, 2013^73^ | Exploratory | No | Yes | Yes | Novel design | No | Confirmed | Yes |
| Rajatanavin, 2013^74^ | BMD | Yes | No | No | Confirmed | No | Confirmed | Yes |

Abbreviations:

BMD- bone mineral density; BMB- bone mineral balance; BMC- bone mineral content; Biochem- laboratory biochemistry tests; Ca balance- calcium balance; TBC- total body calcium; Callus- callus formation in fracture healing; Col aden- colorectal adenoma; Confirmed- hypothesis already confirmed in previous trials; Secondary- secondary endpoint from novel randomised controlled trials; Size- trial too small to measure effect with adequate precision; Fracture study- large randomised controlled trial with fracture as primary endpoint.

**Table S5: Classification and characteristics of observational studies of calcium intake and bone mineral density or fracture**

|  | **Study design** | | | **Endpoint** | |  |  |  |  |  |
| --- | --- | --- | --- | --- | --- | --- | --- | --- | --- | --- |
| **Study** | **Cross-sectional** | **Case-control** | **Cohort** | **Fracture** | **BMD** | **Study purpose** | **Novel** | **Reason** | **Add new clinical information** | **Waste** |
| Wootton, 1979^75^ |  | Yes |  | Yes |  | Fracture risk |  |  |  |  |
| Paganini-Hill, 1981^76^ |  | Yes |  | Yes |  | Fracture risk |  |  |  |  |
| Riggs, 1982^77^ |  |  | Yes | Yes |  | Calcium-fracture |  |  |  |  |
| Aloia, 1985^78^ |  | Yes |  | Yes |  | Fracture risk |  |  |  |  |
| Sandler, 1985^79^ |  |  |  |  | Yes | Calcium-BMD |  |  |  |  |
| Sowers, 1985^80^ |  |  |  |  | Yes | BMD risk |  |  |  |  |
| Yano, 1985^81^ |  |  |  |  | Yes | Calcium-BMD |  |  |  |  |
| Freudenheim, 1986^82^ |  |  | Yes |  | Yes | BMD risk |  |  |  |  |
| Dawson-Hughes, 1987^83^ |  |  | Yes |  | Yes | Calcium-BMD |  |  |  |  |
| Ettinger, 1987^84^ |  |  | Yes |  | Yes | Calcium-BMD |  |  |  |  |
| Lukert, 1987^85^ |  |  | Yes |  | Yes | BMD risk |  |  |  |  |
| Nordin, 1987^86^ |  |  | Yes |  | Yes | BMD risk |  |  |  |  |
| Riggs, 1987^87^ |  |  | Yes |  | Yes | Calcium-BMD |  |  |  |  |
| Angus, 1988^88^ |  |  |  |  | Yes | BMD risk |  |  |  |  |
| Cooper, 1988^89^ |  | Yes |  | Yes |  | Fracture risk |  |  |  |  |
| Holbrook, 1988^90^ |  |  | Yes | Yes |  | Calcium-fracture |  |  |  |  |
| Lau, 1988^91^ |  | Yes |  | Yes |  | Calcium-fracture |  |  |  |  |
| Stevenson, 1988^92^ |  |  |  |  | Yes | Calcium-BMD |  |  |  |  |
| Tylavsky, 1988^93^ |  |  |  |  | Yes | BMD risk |  |  |  |  |
| Cimino, 1989^94^ |  |  | Yes |  | Yes | Calcium-BMD |  |  |  |  |
| Kleerekoper, 1989^95^ |  |  |  | Yes |  | Fracture risk |  |  |  |  |
| Stevenson, 1989^96^ |  |  |  |  | Yes | BMD risk |  |  |  |  |
| Wickham, 1989^97^ |  |  | Yes | Yes |  | Calcium-fracture |  |  |  |  |
| Lutz, 1990^98^ |  |  |  |  | Yes | Calcium-BMD |  |  |  |  |
| Slemenda, 1990^99^ |  |  |  |  | Yes | BMD risk |  |  |  |  |
| van Beresteijn, 1990^100^ |  |  | Yes |  | Yes | Calcium-BMD |  |  |  |  |
| van Beresteijn, 1990^101^ |  |  |  |  | Yes | Calcium-BMD |  |  |  |  |
| Andon, 1991^102^ |  |  |  |  | Yes | Calcium-BMD |  |  |  |  |
| Chi, 1991^103^ |  | Yes |  | Yes |  | Calcium-fracture |  |  |  |  |
| Hansen, 1991^104^ |  |  | Yes |  | Yes | BMD risk |  |  |  |  |
| Lacey, 1991^105^ |  |  |  |  | Yes | BMD risk |  |  |  |  |
| Paganini-Hill, 1991^106^ |  |  | Yes | Yes |  | Fracture risk |  |  |  |  |
| Wheadon, 1991^107^ |  | Yes |  | Yes |  | Calcium-fracture |  |  |  |  |
| Buchs, 1992^108^ |  |  |  |  | Yes | BMD risk |  |  |  |  |
| Howard, 1992^109^ |  |  |  |  | Yes | Calcium-fracture |  |  |  |  |
| Kanis, 1992^110^ |  | Yes |  | Yes |  | Calcium-fracture |  |  |  |  |
| Kelsey, 1992^111^ |  |  | Yes | Yes |  | Fracture risk |  |  |  |  |
| Kreiger, 1992^112^ |  | Yes |  | Yes |  | Fracture risk |  |  |  |  |
| Nieves, 1992^113^ |  | Yes |  | Yes |  | Fracture risk |  |  |  |  |
| Reid, 1992^114^ |  |  |  |  | Yes | BMD risk |  |  |  |  |
| Spector, 1992^115^ |  |  | Yes |  | Yes | Calcium-BMD |  |  |  |  |
| Bauer, 1993^116^ |  |  |  |  | Yes | BMD risk |  |  |  |  |
| Hernandez-Avila, 1993^117^ |  |  |  |  | Yes | BMD risk |  |  |  |  |
| Hu, 1993^118^ |  |  |  |  | Yes | Calcium-BMD |  |  |  |  |
| Jaglal, 1993^119^ |  | Yes |  | Yes |  | Fracture risk |  |  |  |  |
| Jonsson, 1993^120^ |  |  |  | Yes |  | Fracture risk |  |  |  |  |
| Lau, 1993^121^ |  | Yes |  | Yes |  | Calcium-fracture |  |  |  |  |
| Looker, 1993^122^ |  |  | Yes | Yes |  | Calcium-fracture |  |  |  |  |
| Nordin, 1993^123^ |  |  | Yes |  | Yes | BMD risk |  |  |  |  |
| Ooms, 1993^124^ |  |  |  |  | Yes | BMD risk |  |  |  |  |
| Perez Cano, 1993^125^ |  | Yes |  | Yes |  | Calcium-fracture |  |  |  |  |
| Prince, 1993^126^ |  |  |  |  | Yes | BMD risk |  |  |  |  |
| Ribot, 1993^127^ |  | Yes |  | Yes |  | Fracture risk |  |  |  |  |
| Shaw, 1993^128^ |  |  |  |  | Yes | BMD risk |  |  |  |  |
| Sowers, 1993^129^ |  |  | Yes |  | Yes | BMD risk |  |  |  |  |
| Stracke, 1993^130^ |  | Yes |  |  | Yes | Calcium-BMD |  |  |  |  |
| Wyshak, 1993^131^ |  | Yes |  | Yes |  | Calcium-fracture |  |  |  |  |
| Cumming, 1994^132^ |  | Yes |  | Yes |  | Fracture risk |  |  |  |  |
| Gilfillan, 1994^133^ |  |  |  |  | Yes | BMD risk |  |  |  |  |
| Hu, 1994^134^ |  |  |  |  | Yes | Calcium-BMD |  |  |  |  |
| Kroger, 1994^135^ |  |  |  |  | Yes | BMD risk |  |  |  |  |
| Murphy, 1994^136^ |  |  |  |  | Yes | Calcium-BMD |  |  |  |  |
| Nguyen, 1994^137^ |  |  |  |  | Yes | BMD risk |  |  |  |  |
| Reid, 1994^138^ |  |  | Yes |  | Yes | BMD risk |  |  |  |  |
| Soroko, 1994^139^ |  |  |  |  | Yes | Calcium-BMD |  |  |  |  |
| Tranquilli, 1994^140^ |  |  |  |  | Yes | Calcium-BMD |  |  |  |  |
| Cummings, 1995^141^ |  |  | Yes | Yes |  | Fracture risk |  |  |  |  |
| Davis, 1995^142^ |  |  | Yes |  | Yes | Calcium-BMD |  |  |  |  |
| Devine, 1995^143^ |  |  | Yes |  | Yes | Calcium-BMD |  |  |  |  |
| Glynn, 1995^144^ |  |  |  |  | Yes | BMD risk |  |  |  |  |
| Greendale, 1995^145^ |  |  |  |  | Yes | BMD risk |  |  |  |  |
| Ho, 1995^146^ |  |  |  |  | Yes | BMD risk |  |  |  |  |
| Holbrook, 1995^147^ |  |  |  |  | Yes | Calcium-BMD |  |  |  |  |
| Johnell, 1995^148^ |  | Yes |  | Yes |  | Fracture risk |  |  |  |  |
| Meyer, 1995^149^ |  | Yes |  | Yes |  | Fracture risk |  |  |  |  |
| Michaelsson, 1995^150^ |  | Yes |  | Yes |  | Fracture risk |  |  |  |  |
| Tavani, 1995^151^ |  | Yes |  | Yes |  | Calcium-fracture |  |  |  |  |
| Vico, 1995^152^ |  |  |  |  | Yes | BMD risk |  |  |  |  |
| Ward, 1995^153^ |  |  |  |  | Yes | BMD risk |  |  |  |  |
| Bendavid, 1996^154^ |  |  |  |  | Yes | BMD risk |  |  |  |  |
| Chan, 1996^155^ |  | Yes |  | Yes |  | Calcium-fracture |  |  |  |  |
| Clemente, 1996^156^ |  | Yes |  |  | Yes | Calcium-BMD |  |  |  |  |
| Cooper, 1996^157^ |  |  |  |  | Yes | BMD risk |  |  |  |  |
| Cosman, 1996^158^ |  |  | Yes |  | Yes | BMD risk |  |  |  |  |
| Honkanen, 1996^159^ |  |  |  |  | Yes | BMD risk |  |  |  |  |
| Hoover, 1996^160^ |  |  |  |  | Yes | Calcium-BMD |  |  |  |  |
| Huang, 1996^161^ |  |  | Yes | Yes |  | Fracture risk |  |  |  |  |
| Nguyen, 1996^162^ |  |  | Yes |  | Yes | BMD risk |  |  |  |  |
| O'Neill, 1996^163^ |  | Yes |  | Yes |  | Fracture risk |  |  |  |  |
| Orwoll, 1996^164^ |  |  |  |  | Yes | BMD risk |  |  |  |  |
| Pouilles, 1996^165^ |  |  | Yes |  | Yes | BMD risk |  |  |  |  |
| Sone, 1996^166^ |  |  |  |  | Yes | Calcium-BMD |  |  |  |  |
| Ueda, 1996^167^ |  |  |  |  | Yes | BMD risk |  |  |  |  |
| Ulrich, 1996^168^ |  |  |  |  | Yes | Calcium-BMD |  |  |  |  |
| Valdivia, 1996^169^ |  | Yes |  | Yes |  | Fracture risk |  |  |  |  |
| Cumming, 1997^170^ |  |  | Yes | Yes |  | Calcium-fracture |  |  |  |  |
| Earnshaw, 1997^171^ |  |  |  |  | Yes | Calcium-BMD |  |  |  |  |
| Fujiwara, 1997^172^ |  |  | Yes | Yes |  | Fracture risk |  |  |  |  |
| Meyer, 1997^173^ |  |  | Yes | Yes |  | Calcium-fracture |  |  |  |  |
| Michaelsson, 1997^174^ |  |  |  |  | Yes | Calcium-BMD |  |  |  |  |
| Owusu, 1997^175^ |  |  | Yes | Yes |  | Calcium-fracture |  |  |  |  |
| Suleiman, 1997^176^ |  |  |  |  | Yes | Calcium-BMD |  |  |  |  |
| Suzuki, 1997^177^ |  | Yes |  | Yes |  | Fracture risk |  |  |  |  |
| Wang, 1997^178^ |  |  |  |  | Yes | Calcium-BMD |  |  |  |  |
| Burger, 1998^179^ |  |  | Yes |  | Yes | BMD risk |  |  |  |  |
| Clark, 1998^180^ |  | Yes |  | Yes |  | Fracture risk |  |  |  |  |
| Hosking, 1998^181^ |  |  | Yes |  | Yes | Calcium-BMD |  |  |  |  |
| Lau, 1998^182^ |  |  |  |  | Yes | BMD risk |  |  |  |  |
| Masaryk, 1998^183^ |  |  |  |  | Yes | BMD risk |  |  |  |  |
| Mosquera, 1998^184^ |  | Yes |  | Yes |  | Fracture risk |  |  |  |  |
| Mussolino, 1998^185^ |  |  | Yes | Yes |  | Fracture risk |  |  |  |  |
| Rodriguez, 1998^186^ |  |  | Yes |  | Yes | Calcium-BMD |  |  |  |  |
| Turner, 1998^187^ |  |  |  | Yes |  | Fracture risk |  |  |  |  |
| Turner, 1998^187^ |  |  |  | Yes |  | Fracture risk |  |  |  |  |
| Turner, 1998^187^ |  |  |  | Yes |  | Fracture risk |  |  |  |  |
| Uusi-Rasi, 1998^188^ |  |  |  |  | Yes | Calcium-BMD |  |  |  |  |
| Aptel, 1999^189^ |  |  |  |  | Yes | Calcium-BMD |  |  |  |  |
| Bernad, 1999^190^ |  |  |  |  | Yes | Calcium-BMD |  |  |  |  |
| Brot, 1999^191^ |  |  |  |  | Yes | Calcium-BMD |  |  |  |  |
| Dennison, 1999^192^ |  |  | Yes |  | Yes | BMD risk |  |  |  |  |
| Goulding, 1999^193^ |  |  | Yes |  | Yes | BMD risk |  |  |  |  |
| Kanis, 1999^194^ |  | Yes |  | Yes |  | Fracture risk |  |  |  |  |
| Melhus, 1999^195^ |  | Yes |  | Yes |  | Fracture risk |  |  |  |  |
| Metz, 1999^196^ |  |  |  |  | Yes | Calcium-BMD |  |  |  |  |
| Munger, 1999^197^ |  |  | Yes | Yes |  | Fracture risk |  |  |  |  |
| Turner, 1999^198^ |  |  |  | Yes |  | Calcium-fracture |  |  |  |  |
| Varenna, 1999^199^ |  |  |  |  | Yes | BMD risk |  |  |  |  |
| Di Monaco, 2000^200^ |  |  |  |  | Yes | BMD risk | No |  | No | Yes |
| Dirschl, 2000^201^ |  |  | Yes |  | Yes | BMD risk | No |  | No | Yes |
| Guthrie, 2000^202^ |  |  | Yes |  | Yes | BMD risk | No |  | No | Yes |
| Hannan, 2000^203^ |  |  | Yes |  | Yes | BMD risk | No |  | No | Yes |
| Honkanen, 2000^204^ |  |  | Yes | Yes |  | Fracture risk | No |  | No | Yes |
| Huo, 2000^205^ |  | Yes |  | Yes |  | Fracture risk | No |  | No | Yes |
| Huopio, 2000^206^ |  |  | Yes | Yes |  | Fracture risk | Yes | Novel population | Yes | No |
| Huuskonen, 2000^207^ |  |  |  |  | Yes | BMD risk | No |  | No | Yes |
| Jaime, 2000^208^ |  |  |  |  | Yes | Calcium-BMD | No |  | No | Yes |
| Kato, 2000^209^ |  |  | Yes | Yes |  | Fracture risk | No |  | No | Yes |
| Nguyen, 2000^210^ |  |  |  |  | Yes | Calcium-BMD | No |  | No | Yes |
| Orwoll, 2000^211^ |  |  |  |  | Yes | BMD risk | No |  | No | Yes |
| Picard, 2000^212^ |  |  | Yes |  | Yes | BMD risk | No |  | No | Yes |
| Jitapunkul, 2001^213^ |  | Yes |  | Yes |  | Fracture risk | No |  | No | Yes |
| Lau, 2001^214^ |  | Yes |  | Yes |  | Fracture risk | No |  | No | Yes |
| Lumbers, 2001^215^ |  | Yes |  | Yes |  | Fracture risk | No |  | No | Yes |
| Lunt, 2001^216^ |  |  |  | Yes | Yes | Calcium-fracture | No |  | No | Yes |
| Nguyen, 2001^217^ |  |  | Yes | Yes |  | Fracture risk | No |  | No | Yes |
| Piaseu, 2001^218^ |  |  |  |  | Yes | Calcium-BMD | No |  | No | Yes |
| Ramalho, 2001^219^ |  | Yes |  | Yes |  | Fracture risk | No |  | No | Yes |
| Sasaki, 2001^220^ |  |  |  |  | Yes | BMD risk | No |  | No | Yes |
| Tanaka, 2001^221^ |  |  |  |  | Yes | BMD risk | No |  | No | Yes |
| Varenna, 2001^222^ |  | Yes |  | Yes | Yes | Calcium-fracture | No |  | No | Yes |
| Vestergaard, 2001^223^ |  |  |  |  | Yes | BMD risk | No |  | No | Yes |
| Blain, 2002^224^ |  |  |  |  | Yes | BMD risk | No |  | No | Yes |
| Dargent-Molina, 2002^225^ |  |  | Yes | Yes |  | Fracture risk | Yes | Predictive model | Yes | No |
| del Puente, 2002^226^ |  |  | Yes |  | Yes | Calcium-BMD | No |  | No | Yes |
| Ilich-Ernst, 2002^227^ |  |  |  |  | Yes | Calcium-BMD | No |  | No | Yes |
| Lee, 2002^228^ |  |  | Yes | Yes |  | Fracture risk | No |  | No | Yes |
| Mendez, 2002^229^ |  |  |  |  | Yes | Calcium-BMD | No |  | No | Yes |
| Roman Garcia, 2002^230^ |  |  |  | Yes |  | Fracture risk | No |  | No | Yes |
| Wu, 2002^231^ |  |  | Yes |  | Yes | BMD risk | No |  | No | Yes |
| Albrand, 2003^232^ |  |  | Yes | Yes |  | Fracture risk | No |  | No | Yes |
| Ballard, 2003^233^ |  |  |  |  | Yes | BMD risk | No |  | No | Yes |
| Feskanich, 2003^234^ |  |  | Yes | Yes |  | Calcium-fracture | No |  | No | Yes |
| Ilich, 2003^235^ |  |  |  |  | Yes | BMD risk | No |  | No | Yes |
| Knoke, 2003^236^ |  |  | Yes |  | Yes | BMD risk | No |  | No | Yes |
| Korpelainen, 2003^237^ |  |  |  |  | Yes | BMD risk | No |  | No | Yes |
| MacInnis, 2003^238^ |  |  |  |  | Yes | BMD risk | No |  | No | Yes |
| Melton, 2003^239^ |  |  | Yes | Yes |  | Fracture risk | No |  | No | Yes |
| Michaelsson, 2003^240^ |  |  | Yes | Yes |  | Calcium-fracture | No |  | No | Yes |
| Roy, 2003^241^ |  |  | Yes | Yes |  | Fracture risk | No |  | No | Yes |
| Runyan, 2003^242^ |  |  |  |  | Yes | BMD risk | No |  | No | Yes |
| Sirola, 2003^243^ |  |  | Yes |  | Yes | Calcium-BMD | No |  | No | Yes |
| Sirola, 2003^244^ |  |  | Yes |  | Yes | Calcium-BMD | No |  | No | Yes |
| Suzuki, 2003^245^ |  |  |  |  | Yes | Calcium-BMD | No |  | No | Yes |
| Bakhireva, 2004^246^ |  |  | Yes |  | Yes | BMD risk | No |  | No | Yes |
| Bhattoa, 2004^247^ |  |  |  |  | Yes | BMD risk | No |  | No | Yes |
| Chu, 2004^248^ |  | Yes |  | Yes |  | Fracture risk | No |  | No | Yes |
| Devine, 2004^249^ |  |  |  |  | Yes | Calcium-BMD | No |  | No | Yes |
| Hagino, 2004^250^ |  | Yes |  | Yes |  | Fracture risk | No |  | No | Yes |
| Ho, 2004^251^ |  |  | Yes |  | Yes | Calcium-BMD | No |  | No | Yes |
| Kurabayashi, 2004^252^ |  |  | Yes |  | Yes | Calcium-BMD | No |  | No | Yes |
| Lopez-Caudana, 2004^253^ |  |  |  |  | Yes | BMD risk | No |  | No | Yes |
| Luetters, 2004^254^ |  | Yes |  | Yes |  | Fracture risk | No |  | No | Yes |
| Macdonald, 2004^255^ |  |  | Yes |  | Yes | BMD risk | No |  | No | Yes |
| McCabe, 2004^256^ |  |  |  |  | Yes | Calcium-BMD | No |  | No | Yes |
| Pongchaiyakul, 2004^257^ |  |  |  |  | Yes | BMD risk | No |  | No | Yes |
| Van der Klift, 2004^258^ |  |  | Yes | Yes |  | Fracture risk | No |  | No | Yes |
| Wengreen, 2004^259^ |  | Yes |  | Yes |  | Fracture risk | No |  | No | Yes |
| Yarbrough, 2004^260^ |  |  |  |  | Yes | BMD risk | No |  | No | Yes |
| Cauley, 2005^261^ |  |  |  |  | Yes | BMD risk | No |  | No | Yes |
| Chen, 2005^262^ |  |  |  |  | Yes | BMD risk | No |  | No | Yes |
| Cheung, 2005^263^ |  |  |  |  | Yes | BMD risk | No |  | No | Yes |
| Cussler, 2005^264^ |  |  | Yes |  | Yes | Calcium-BMD | No |  | No | Yes |
| Hassa, 2005^265^ |  |  |  |  | Yes | BMD risk | No |  | No | Yes |
| Kanis, 2005^266^ |  |  | Yes | Yes |  | Calcium-fracture | No |  | No | Yes |
| Kelsey, 2005^267^ |  | Yes |  | Yes |  | Fracture risk | No |  | No | Yes |
| Kung, 2005^268^ |  |  |  |  | Yes | BMD risk | Yes | Predictive model | No | Yes |
| Lau, 2005^269^ |  |  |  |  | Yes | BMD risk | No |  | No | Yes |
| Meier, 2005^270^ |  | Yes |  | Yes |  | Fracture risk | No |  | No | Yes |
| Morita, 2005^271^ |  |  | Yes |  | Yes | BMD risk | No |  | No | Yes |
| Naves, 2005^272^ |  |  | Yes |  | Yes | BMD risk | No |  | No | Yes |
| Ofluoglu, 2005^273^ |  |  |  |  | Yes | BMD risk | No |  | No | Yes |
| Papaioannou, 2005^274^ |  |  | Yes | Yes |  | Fracture risk | No |  | No | Yes |
| Romero, 2005^275^ |  |  |  |  | Yes | BMD risk | No |  | No | Yes |
| Uusi-Rasi, 2005^276^ |  |  | Yes |  | Yes | BMD risk | No |  | No | Yes |
| Abraham, 2006^277^ |  |  | Yes |  | Yes | BMD risk | No |  | No | Yes |
| Babbar, 2006^278^ |  |  |  |  | Yes | BMD risk | No |  | No | Yes |
| Dong, 2006^279^ |  |  |  |  | Yes | BMD risk | No |  | No | Yes |
| Jaime, 2006^280^ |  |  |  |  | Yes | Calcium-fracture | No |  | No | Yes |
| Lau, 2006^281^ |  |  |  |  | Yes | BMD risk | No |  | No | Yes |
| Michaelsson, 2006^282^ |  |  | Yes |  | Yes | BMD risk | Yes | Novel study design | No | Yes |
| Nakamura, 2006^283^ |  |  |  |  | Yes | BMD risk | No |  | No | Yes |
| Van Geel, 2006^284^ |  |  | Yes | Yes |  | Fracture risk | No |  | No | Yes |
| Wang, 2006^285^ |  |  |  |  | Yes | Calcium-BMD | No |  | No | Yes |
| Xu, 2006^286^ |  | Yes |  | Yes |  | Calcium-fracture | No |  | No | Yes |
| Angbratt, 2007^287^ |  |  |  |  | Yes | Calcium-BMD | No |  | No | Yes |
| Cauley, 2007^288^ |  |  | Yes | Yes |  | Fracture risk | No |  | No | Yes |
| Center, 2007^289^ |  |  | Yes | Yes |  | Fracture risk | Yes | Novel population | No | Yes |
| Choi, 2007^290^ |  |  |  |  | Yes | BMD risk | No |  | No | Yes |
| Diez-Perez, 2007^291^ |  |  | Yes | Yes |  | Fracture risk | No |  | No | Yes |
| Fominykh, 2007^292^ |  |  |  |  | Yes | BMD risk | No |  | No | Yes |
| Frazao, 2007^293^ |  |  |  |  | Yes | BMD risk | No |  | No | Yes |
| Gu, 2007^294^ |  |  |  |  | Yes | BMD risk | No |  | No | Yes |
| Hamdi Kara, 2007^295^ |  |  |  |  | Yes | BMD risk | No |  | No | Yes |
| Key, 2007^296^ |  |  | Yes | Yes |  | Calcium-fracture | No |  | No | Yes |
| Kung, 2007^297^ |  |  | Yes | Yes |  | Fracture risk | No |  | No | Yes |
| Lewis, 2007^298^ |  |  | Yes | Yes |  | Fracture risk | No |  | No | Yes |
| Masoni, 2007^299^ |  | Yes |  | Yes |  | Fracture risk | No |  | No | Yes |
| Napoli, 2007^300^ |  |  |  |  | Yes | Calcium-BMD | No |  | No | Yes |
| Nguyen, 2007^301^ |  |  | Yes | Yes |  | Fracture risk | No |  | No | Yes |
| Nurzenski, 2007^302^ |  |  |  |  | Yes | Calcium-BMD | Yes | Novel study design | No | Yes |
| Saitoglu, 2007^303^ |  |  |  |  | Yes | BMD risk | No |  | No | Yes |
| Van Geel, 2007^304^ |  |  | Yes | Yes |  | Fracture risk | No |  | No | Yes |
| Varenna, 2007^305^ |  |  |  |  | Yes | Calcium-BMD | No |  | No | Yes |
| Bianco, 2008^306^ |  |  |  |  | Yes | BMD risk | No |  | No | Yes |
| Dargent-Molina, 2008^307^ |  |  | Yes | Yes |  | Calcium-fracture | No |  | No | Yes |
| El Maghraoui, 2008^308^ |  |  |  | Yes |  | Fracture risk | No |  | No | Yes |
| Farrin, 2008^309^ |  |  |  |  | Yes | BMD risk | No |  | No | Yes |
| Keramat, 2008^310^ |  | Yes |  |  | Yes | BMD risk | No |  | No | Yes |
| Kim, 2008^311^ |  | Yes |  |  | Yes | BMD risk | No |  | No | Yes |
| Ma, 2008^312^ |  |  |  |  | Yes | BMD risk | No |  | No | Yes |
| Meier, 2008^313^ |  |  | Yes | Yes |  | Fracture risk | No |  | No | Yes |
| Nieves, 2008^314^ |  |  | Yes | Yes | Yes | Calcium-fracture | No |  | No | Yes |
| Ozdemir, 2008^315^ |  |  |  |  | Yes | Calcium-BMD | No |  | No | Yes |
| Pongchaiyakul, 2008^316^ |  |  |  |  | Yes | Calcium-BMD | No |  | No | Yes |
| Uusi-Rasi, 2008^317^ |  |  | Yes |  | Yes | Calcium-BMD | No |  | No | Yes |
| Wang, 2008^318^ |  |  |  |  | Yes | BMD risk | No |  | No | Yes |
| Zhai, 2008^319^ |  |  | Yes |  | Yes | BMD risk | No |  | No | Yes |
| Atalar, 2009^320^ |  |  |  |  | Yes | BMD risk | No |  | No | Yes |
| Duncea, 2009^321^ |  |  |  |  | Yes | Calcium-BMD | No |  | No | Yes |
| Farrell, 2009^322^ |  |  |  |  | Yes | BMD risk | No |  | No | Yes |
| Hejazi, 2009^323^ |  |  |  |  | Yes | BMD risk | No |  | No | Yes |
| Ho-Pham, 2009^324^ |  |  |  | Yes |  | Fracture risk | No |  | No | Yes |
| Ho-Pham, 2009^325^ |  |  |  |  | Yes | BMD risk | No |  | No | Yes |
| Koh, 2009^326^ |  |  | Yes | Yes |  | Calcium-fracture | No |  | No | Yes |
| Lopes, 2009^327^ |  |  |  | Yes |  | Fracture risk | No |  | No | Yes |
| Mavroeidi, 2009^328^ |  |  |  |  | Yes | Calcium-BMD | No |  | No | Yes |
| Nakamura, 2009^329^ |  |  | Yes | Yes |  | Calcium-fracture | No |  | No | Yes |
| Nawata, 2009^330^ |  |  |  |  | Yes | Calcium-BMD | No |  | No | Yes |
| Pinheiro, 2009^331^ |  |  |  | Yes |  | Fracture risk | No |  | No | Yes |
| Popov, 2009^332^ |  |  |  |  | Yes | BMD risk | No |  | No | Yes |
| Thomas-John, 2009^333^ |  |  | Yes | Yes | Yes | Fracture risk | No |  | No | Yes |
| Zhong, 2009^334^ |  |  |  | Yes |  | Calcium-fracture | No |  | No | Yes |
| Cauley, 2010^335^ |  |  |  |  | Yes | BMD risk | No |  | No | Yes |
| Chee, 2010^336^ |  |  |  |  | Yes | Calcium-BMD | No |  | No | Yes |
| Fardellone, 2010^337^ |  |  |  | Yes |  | Calcium-fracture | No |  | No | Yes |
| Gronskag, 2010^338^ |  |  | Yes | Yes | Yes | Calcium-fracture | No |  | No | Yes |
| Jha, 2010^339^ |  | Yes |  | Yes |  | Fracture risk | No |  | No | Yes |
| Lan, 2010^340^ |  | Yes |  | Yes |  | Fracture risk | No |  | No | Yes |
| Sahni, 2010^341^ |  |  | Yes | Yes |  | Calcium-fracture | No |  | No | Yes |
| Shin, 2010^342^ |  |  |  |  | Yes | BMD risk | No |  | No | Yes |
| Shin, 2010^343^ |  |  |  |  | Yes | BMD risk | No |  | No | Yes |
| Shubeska-Stratrova, 2010^344^ |  |  |  |  | Yes | Calcium-BMD | No |  | No | Yes |
| Aggarwal, 2011^345^ |  |  |  |  | Yes | BMD risk | No |  | No | Yes |
| Alissa, 2011^346^ |  |  |  |  | Yes | BMD risk | No |  | No | Yes |
| Benetou, 2011^347^ |  |  | Yes | Yes |  | Fracture risk | No |  | No | Yes |
| Chan, 2011^348^ |  |  | Yes |  | Yes | BMD risk | No |  | No | Yes |
| Fairweather-Tait, 2011^349^ |  |  |  |  | Yes | Calcium-BMD | No |  | No | Yes |
| Khoo, 2011^350^ |  |  |  |  | Yes | BMD risk | No |  | No | Yes |
| Kotsalou, 2011^351^ |  |  |  |  | Yes | Calcium-BMD | No |  | No | Yes |
| Nakamura, 2011^352^ |  |  | Yes | Yes |  | Fracture risk | No |  | No | Yes |
| Ostertag, 2011^353^ |  |  | Yes |  | Yes | BMD risk | Yes | Novel population | No | Yes |
| Park, 2011^354^ |  | Yes |  |  | Yes | Calcium-BMD | No |  | No | Yes |
| Perez Durillo, 2011^355^ |  | Yes |  | Yes |  | BMD risk | No |  | No | Yes |
| Schwetz, 2011^356^ |  |  | Yes |  | Yes | BMD risk | No |  | No | Yes |
| Simoes, 2011^357^ |  | Yes |  | Yes |  | Calcium-fracture | No |  | No | Yes |
| Tsang, 2011^358^ |  |  |  | Yes |  | Fracture risk | No |  | No | Yes |
| Warensjo, 2011^359^ |  |  | Yes | Yes | Yes | Calcium-fracture | No |  | No | Yes |
| Yazdani, 2011^360^ |  |  |  |  | Yes | BMD risk | No |  | No | Yes |
| Anderson, 2012^361^ |  |  |  |  | Yes | Calcium-BMD | No |  | No | Yes |
| Arabi, 2012^362^ |  |  | Yes |  | Yes | BMD risk | No |  | No | Yes |
| Connie, 2012^363^ |  |  |  |  | Yes | BMD risk | No |  | No | Yes |
| Khan, 2012^364^ |  |  | Yes | Yes |  | Calcium-fracture | No |  | No | Yes |
| Martinez-Ramirez, 2012^365^ |  | Yes |  | Yes |  | Calcium-fracture | No |  | No | Yes |
| Marwaha, 2012^366^ |  | Yes |  | Yes |  | Fracture risk | No |  | No | Yes |
| Mihalcea, 2012^367^ |  |  |  |  | Yes | BMD risk | No |  | No | Yes |
| Nakamura, 2012^368^ |  |  | Yes |  | Yes | BMD risk | No |  | No | Yes |
| Rouzi, 2012^369^ |  |  | Yes | Yes |  | Fracture risk | No |  | No | Yes |
| Sarkis, 2012^370^ |  | Yes |  |  | Yes | BMD risk | No |  | No | Yes |
| Thomas, 2012^371^ |  |  | Yes |  | Yes | Calcium-BMD | No |  | No | Yes |
| Castro-Lionard, 2013^372^ |  |  |  | Yes |  | Fracture risk | No |  | No | Yes |
| Chatterjee, 2013^373^ |  |  |  |  | Yes | Calcium-BMD | No |  | No | Yes |
| Ebeling, 2013^374^ |  |  |  | Yes | Yes | Calcium-BMD | No |  | No | Yes |
| Feart, 2013^375^ |  |  | Yes | Yes |  | Fracture risk | No |  | No | Yes |
| Gonnelli, 2013^376^ |  |  |  |  | Yes | BMD risk | No |  | No | Yes |
| Jakobsen, 2013^377^ |  |  |  | Yes |  | Fracture risk | No |  | No | Yes |
| Kwok, 2013^378^ |  |  |  | Yes |  | Fracture risk | No |  | No | Yes |
| Onat, 2013^379^ |  |  |  |  | Yes | BMD risk | No |  | No | Yes |
| Prentice, 2013^73^ |  |  | Yes | Yes |  | Calcium-fracture | No |  | No | Yes |
| Quesada-Gomez, 2013^380^ |  | Yes |  |  | Yes | Calcium-BMD | No |  | No | Yes |
| Sahni, 2013^381^ |  |  | Yes | Yes | Yes | Calcium-fracture | No |  | No | Yes |
| Samieri, 2013^382^ |  |  | Yes | Yes |  | Fracture risk | No |  | No | Yes |
| Speer, 2013^383^ |  |  |  |  | Yes | Calcium-BMD | No |  | No | Yes |
| Tajik, 2013^384^ |  |  |  |  | Yes | Calcium-BMD | No |  | No | Yes |
| Tseng, 2013^385^ |  | Yes |  | Yes |  | Fracture risk | No |  | No | Yes |
| Vyskocil, 2013^386^ |  |  |  |  | Yes | Calcium-BMD | No |  | No | Yes |
| Zhou, 2013^387^ |  |  | Yes |  | Yes | Calcium-BMD | No |  | No | Yes |
| Alissa, 2014^388^ |  |  |  |  | Yes | BMD risk | No |  | No | Yes |
| Betancourt Ortiz, 2014^389^ |  |  |  |  | Yes | Calcium-BMD | No |  | No | Yes |
| Borges, 2014^390^ |  |  |  | Yes |  | Fracture risk | No |  | No | Yes |
| Domiciano, 2014^391^ |  |  | Yes | Yes |  | Fracture risk | No |  | No | Yes |
| Gunn, 2014^392^ |  |  |  |  | Yes | BMD risk | No |  | No | Yes |
| Hassan, 2014^393^ |  | Yes |  | Yes |  | Fracture risk | No |  | No | Yes |
| Kim, 2014^394^ |  |  |  | Yes |  | Fracture risk | No |  | No | Yes |
| Kim, 2014^395^ |  |  |  |  | Yes | Calcium-BMD | No |  | No | Yes |
| Paknahad, 2014^396^ |  |  |  |  | Yes | BMD risk | No |  | No | Yes |
| Radavelli-Bagatini, 2014^397^ |  |  |  |  | Yes | Calcium-BMD | No |  | No | Yes |
| Sahni, 2014^398^ |  |  | Yes | Yes |  | Calcium-fracture | No |  | No | Yes |
| Shankar, 2014^399^ |  | Yes |  | Yes |  | Calcium-fracture | No |  | No | Yes |
| Skowronska-Jozwiak, 2014^400^ |  |  |  | Yes | Yes | Calcium-fracture | No |  | No | Yes |
| van den Berg, 2014^401^ |  |  |  |  | Yes | Calcium-BMD | No |  | No | Yes |
| Wlodarek, 2014^402^ |  |  |  | Yes | Yes | Calcium-fracture | No |  | No | Yes |

Abbreviations: BMD- bone mineral density; Fracture Risk- investigate determinants of fracture risk or relationships between risk factors and fracture; BMD risk- investigate determinants of BMD or relationships between risk factors and BMD; Calcium-fracture- determine relationship between calcium intake and fracture risk; Calcium-BMD- determine relationship between calcium intake and BMD.

**Table S6: Classification and characteristics of randomised controlled trials of vitamin D supplements and bone mineral density or fracture**

| **Study** | **Primary**  **endpoint** | **BMD**  **data** | **Fracture**  **data** | **Novel** | **Reason** | **Add new clinical**  **knowledge** | **Reason** | **Waste** |
| --- | --- | --- | --- | --- | --- | --- | --- | --- |
| Christiansen, 1973^403^ | BMC | Yes |  |  |  |  |  |  |
| Alhava, 1975^404^ | BMD | Yes |  |  |  |  |  |  |
| Christiansen, 1980^405^ | BMC | Yes |  |  |  |  |  |  |
| Takizawa, 1980^406^ | BMD | Yes |  |  |  |  |  |  |
| Smith, 1981^3^ | BMC | Yes |  |  |  |  |  |  |
| Inkovaara, 1983^4^ | Biochem |  | Yes |  |  |  |  |  |
| Mobarhan, 1984^407^ | BMD | Yes |  |  |  |  |  |  |
| Orwoll, 1990^12^ | BMD | Yes |  |  |  |  |  |  |
| Dawson-Hughes, 1991^408^ | BMD | Yes |  |  |  |  |  |  |
| Chapuy, 1994^20^ | Fracture |  | Yes |  |  |  |  |  |
| Dawson-Hughes, 1995^409^ | BMD | Yes |  |  |  |  |  |  |
| Vogelsang, 1995^410^ | BMD | Yes |  |  |  |  |  |  |
| Adachi, 1996^411^ | BMD | Yes |  |  |  |  |  |  |
| Bernstein, 1996^412^ | BMD | Yes |  |  |  |  |  |  |
| Buckley, 1996^413^ | BMD | Yes |  |  |  |  |  |  |
| Lips, 1996^414^ | Fracture |  | Yes |  |  |  |  |  |
| Dawson-Hughes, 1997^27^ | BMD |  | Yes |  |  |  |  |  |
| Mautalen, 1997^415^ | BMD | Yes |  |  |  |  |  |  |
| Baeksgaard, 1998^28^ | BMD | Yes |  |  |  |  |  |  |
| Komulainen, 1998^416^ | BMD |  | Yes |  |  |  |  |  |
| Tuppurainen, 1998^417^ | BMD | Yes |  |  |  |  |  |  |
| Krieg, 1999^418^ | BMD | Yes |  |  |  |  |  |  |
| Hunter, 2000^419^ | BMD | Yes |  |  |  |  |  |  |
| Patel, 2001^420^ | BMD | Yes |  |  |  |  |  |  |
| Chapuy, 2002^39^ | Biochem |  | Yes |  |  |  |  |  |
| Meyer, 2002^421^ | Fracture |  | Yes |  |  |  |  |  |
| Cooper, 2003^422^ | BMD | Yes |  |  |  |  |  |  |
| Grados, 2003^41^ | BMD | Yes |  |  |  |  |  |  |
| Trivedi, 2003^423^ | Fracture |  | Yes |  |  |  |  |  |
| Venkatachalam, 2003^424^ | BMD | Yes |  |  |  |  |  |  |
| Di Daniele, 2004^425^ | BMD | Yes |  |  |  |  |  |  |
| Doetsch, 2004^44^ | Callus | Yes |  |  |  |  |  |  |
| Goode, 2004^426^ | BMD | Yes |  |  |  |  |  |  |
| Haworth, 2004^427^ | BMD | Yes |  |  |  |  |  |  |
| Larsen, 2004^47^ | Fracture |  | Yes |  |  |  |  |  |
| Meier, 2004^48^ | BMD | Yes |  |  |  |  |  |  |
| Grant, 2005^50^ | Fracture |  | Yes | Yes | Fracture study | Yes | Fracture study | No |
| Porthouse, 2005^51^ | Fracture |  | Yes | Yes | Fracture study | Yes | Fracture study | No |
| Wissing, 2005^428^ | BMD | Yes |  | Yes | Novel population | No | Confirmed | Yes |
| Daly, 2006^53^ | BMD | Yes |  | No | Confirmed |  |  | Yes |
| Jackson, 2006^429^ | Fracture |  | Yes | Yes | Fracture study | Yes | Fracture study | No |
| Law, 2006^430^ | Fracture |  | Yes | Yes | Fracture study | Yes | Fracture study | No |
| Mikati, 2006^431^ | BMD | Yes |  | Yes | Novel population | No | Confirmed | Yes |
| Moschonis, 2006^432^ | BMD | Yes |  | No | Confirmed |  |  | Yes |
| Zhang, 2006^433^ | BMD | Yes |  | Yes | Novel design | No | Confirmed | Yes |
| Bolton-Smith, 2007^57^ | BMD | Yes |  | Yes | Novel design | No | Confirmed | Yes |
| Hitz, 2007^59^ | BMD | Yes |  | No | Confirmed |  |  | Yes |
| Lyons, 2007^434^ | Fracture |  | Yes | Yes | Fracture study | Yes | Fracture study | No |
| Smith, 2007^435^ | Fracture |  | Yes | Yes | Fracture study | Yes | Fracture study | No |
| Andersen, 2008^436^ | BMD | Yes |  | Yes | Novel population | Yes | Novel population | No |
| Lappe, 2008^437^ | Fracture |  | Yes | Yes | Fracture study | Yes | Fracture study | No |
| Zhu, 2008^63^ | BMD | Yes |  | No | Confirmed |  |  | Yes |
| Carlin, 2009^438^ | 25D | Yes |  | Yes | Novel design | Yes | Novel design | No |
| Kukuljan, 2009^64^ | BMD | Yes |  | Yes | Novel design | No | Confirmed | Yes |
| Viljakainen, 2009^439^ | BMD | Yes |  | No | Confirmed |  |  | Yes |
| Islam, 2010^440^ | BMD | Yes |  | No | Confirmed |  |  | Yes |
| Salovaara, 2010^68^ | Fracture |  | Yes | Yes | Fracture study | Yes | Fracture study | No |
| Sanders, 2010^441^ | Fracture |  | Yes | Yes | Fracture study | Yes | Fracture study | No |
| Moschonis, 2011^442^ | BMD | Yes |  | No | Confirmed |  |  | Yes |
| Verschueren, 2011^443^ | BMD | Yes |  | Yes | Novel design | No | Confirmed | Yes |
| Grimnes, 2012^444^ | BMD | Yes |  | Yes | Novel design | Yes | Confirmed | No |
| Iuliano-Burns, 2012^445^ | BMD | Yes |  | No | Confirmed |  |  | Yes |
| Nieves, 2012^446^ | BMD | Yes |  | No | Confirmed |  |  | Yes |
| Bolland, 2013^447^ | BMD | Yes |  | Yes | Novel population | No | Confirmed | Yes |
| Wamberg, 2013^448^ | BMD | Yes |  | No | Confirmed |  |  | Yes |
| Czech-Kowalska, 2014^449^ | BMD | Yes |  | Yes | Novel population | No | Confirmed | Yes |
| Gaffney-Stomberg, 2014^450^ | BMD | Yes |  | No | Confirmed |  |  | Yes |
| Mieczkowski, 2014^451^ | BMD | Yes |  | Yes | Novel population | No | Confirmed | Yes |
| Norenstedt, 2014^452^ | BMD | Yes |  | Yes | Novel population | No | Confirmed | Yes |
| Rolighed, 2014^453^ | BMD | Yes |  | Yes | Novel population | No | Confirmed | Yes |
| Chen, 2015^454^ | BMD | Yes |  | No | Confirmed |  |  | Yes |
| Hansen, 2015^455^ | BMD | Yes |  | No | Confirmed |  |  | Yes |
| Overton, 2015^456^ | BMD | Yes |  | Yes | Novel population | No | Confirmed | Yes |
| Rousseau, 2015^457^ | BMD | Yes |  | Yes | Novel population | No | Confirmed | Yes |
| Silk, 2015^458^ | BMD | Yes |  | No | Confirmed |  |  | Yes |

BMD- bone mineral density; BMC- bone mineral content. Biochem- laboratory biochemistry tests; Callus- callus formation in fracture healing; Confirmed- hypothesis already confirmed in previous trials; Fracture study- large randomised controlled trial with fracture as primary endpoint.

**Figure S1: flow of studies for calcium intake**

**Figure S2: flow of studies for randomised controlled trials of vitamin D supplements**

RCT- randomised controlled trial. One trial (Sato et al. Cerebrovasc Dis 2005;20:187-92) was excluded because multiple other trials from this group have been recently retracted due to concerns about fraudulent data.

**References in Additional file**

1. Recker RR, Saville PD, Heaney RP. Effect of estrogens and calcium carbonate on bone loss in postmenopausal women. Ann Intern Med 1977; 87:649-55.

2. Lamke B, Sjoberg HE, Sylven M. Bone mineral content in women with Colles' fracture: effect of calcium supplementation. Acta Orthop Scand 1978; 49:143-6.

3. Smith EL, Jr., Reddan W, Smith PE. Physical activity and calcium modalities for bone mineral increase in aged women. Med Sci Sports Exerc 1981; 13:60-4.

4. Inkovaara J, Gothoni G, Halttula R, Heikinheimo R, Tokola O. Calcium, vitamin D and anabolic steroid in treatment of aged bones: double-blind placebo-controlled long-term clinical trial. Age Ageing 1983; 12:124-30.

5. Recker RR, Heaney RP. The effect of milk supplements on calcium metabolism, bone metabolism and calcium balance. Am J Clin Nutr 1985; 41:254-63.

6. Hansson T, Roos B. The effect of fluoride and calcium on spinal bone mineral content: a controlled, prospective (3 years) study. Calcif Tissue Int 1987; 40:315-7.

7. Polley KJ, Nordin BE, Baghurst PA, Walker CJ, Chatterton BE. Effect of calcium supplementation on forearm bone mineral content in postmenopausal women: a prospective, sequential controlled trial. J Nutr 1987; 117:1929-35.

8. Riis B, Thomsen K, Christiansen C. Does calcium supplementation prevent postmenopausal bone loss? A double-blind, controlled clinical study. N Engl J Med 1987; 316:173-7.

9. Smith EL, Gilligan C, Smith PE, Sempos CT. Calcium supplementation and bone loss in middle-aged women. Am J Clin Nutr 1989; 50:833-42.

10. Dawson-Hughes B, Dallal GE, Krall EA, Sadowski L, Sahyoun N, Tannenbaum S. A controlled trial of the effect of calcium supplementation on bone density in postmenopausal women. N Engl J Med 1990; 323:878-83.

11. Fujita T, Fukase M, Miyamoto H, Matsumoto T, Ohue T. Increase of bone mineral density by calcium supplement with oyster shell electrolysate. Bone Miner 1990; 11:85-91.

12. Orwoll ES, Oviatt SK, McClung MR, Deftos LJ, Sexton G. The rate of bone mineral loss in normal men and the effects of calcium and cholecalciferol supplementation. Ann Intern Med 1990; 112:29-34.

13. Elders PJ, Netelenbos JC, Lips P, van Ginkel FC, Khoe E, Leeuwenkamp OR, et al. Calcium supplementation reduces vertebral bone loss in perimenopausal women: a controlled trial in 248 women between 46 and 55 years of age. J Clin Endocrinol Metab 1991; 73:533-40.

14. Nelson ME, Fisher EC, Dilmanian FA, Dallal GE, Evans WJ. A 1-y walking program and increased dietary calcium in postmenopausal women: effects on bone. Am J Clin Nutr 1991; 53:1304-11.

15. Prince RL, Smith M, Dick IM, Price RI, Webb PG, Henderson NK, et al. Prevention of postmenopausal osteoporosis. A comparative study of exercise, calcium supplementation, and hormone-replacement therapy. N Engl J Med 1991; 325:1189-95.

16. Chapuy MC, Arlot ME, Duboeuf F, Brun J, Crouzet B, Arnaud S, et al. Vitamin D3 and calcium to prevent hip fractures in the elderly women. N Engl J Med 1992; 327:1637-42.

17. Lau EM, Woo J, Leung PC, Swaminathan R, Leung D. The effects of calcium supplementation and exercise on bone density in elderly Chinese women. Osteoporos Int 1992; 2:168-73.

18. Reid IR, Ames RW, Evans MC, Gamble GD, Sharpe SJ. Effect of calcium supplementation on bone loss in postmenopausal women.[Erratum appears in N Engl J Med 1993 Oct 21;329(17):1281]. N Engl J Med 1993; 328:460-4.

19. Aloia JF, Vaswani A, Yeh JK, Ross PL, Flaster E, Dilmanian FA. Calcium supplementation with and without hormone replacement therapy to prevent postmenopausal bone loss. Ann Intern Med 1994; 120:97-103.

20. Chapuy MC, Arlot ME, Delmas PD, Meunier PJ. Effect of calcium and cholecalciferol treatment for three years on hip fractures in elderly women. BMJ 1994; 308:1081-2.

21. Chevalley T, Rizzoli R, Nydegger V, Slosman D, Rapin CH, Michel JP, et al. Effects of calcium supplements on femoral bone mineral density and vertebral fracture rate in vitamin-D-replete elderly patients. Osteoporos Int 1994; 4:245-52.

22. Strause L, Saltman P, Smith KT, Bracker M, Andon MB. Spinal bone loss in postmenopausal women supplemented with calcium and trace minerals. J Nutr 1994; 124:1060-4.

23. Prince R, Devine A, Dick I, Criddle A, Kerr D, Kent N, et al. The effects of calcium supplementation (milk powder or tablets) and exercise on bone density in postmenopausal women. J Bone Miner Res 1995; 10:1068-75.

24. Fujita T, Ohue T, Fujii Y, Miyauchi A, Takagi Y. Heated oyster shell-seaweed calcium (AAA Ca) on osteoporosis. Calcif Tissue Int 1996; 58:226-30.

25. Perez-Jaraiz MD, Revilla M, Alvarez de los Heros JI, Villa LF, Rico H. Prophylaxis of osteoporosis with calcium, estrogens and/or eelcatonin: comparative longitudinal study of bone mass. Maturitas 1996; 23:327-32.

26. Recker RR, Hinders S, Davies KM, Heaney RP, Stegman MR, Lappe JM, et al. Correcting calcium nutritional deficiency prevents spine fractures in elderly women. J Bone Miner Res 1996; 11:1961-6.

27. Dawson-Hughes B, Harris SS, Krall EA, Dallal GE. Effect of calcium and vitamin D supplementation on bone density in men and women 65 years of age or older. N Engl J Med 1997; 337:670-6.

28. Baeksgaard L, Andersen KP, Hyldstrup L. Calcium and vitamin D supplementation increases spinal BMD in healthy, postmenopausal women. Osteoporos Int 1998; 8:255-60.

29. Ricci TA, Chowdhury HA, Heymsfield SB, Stahl T, Pierson RN, Jr., Shapses SA. Calcium supplementation suppresses bone turnover during weight reduction in postmenopausal women. J Bone Miner Res 1998; 13:1045-50.

30. Riggs BL, O'Fallon WM, Muhs J, O'Connor MK, Kumar R, Melton LJ, 3rd. Long-term effects of calcium supplementation on serum parathyroid hormone level, bone turnover, and bone loss in elderly women. J Bone Miner Res 1998; 13:168-74.

31. Storm D, Eslin R, Porter ES, Musgrave K, Vereault D, Patton C, et al. Calcium supplementation prevents seasonal bone loss and changes in biochemical markers of bone turnover in elderly New England women: a randomized placebo-controlled trial. J Clin Endocrinol Metab 1998; 83:3817-25.

32. Castelo-Branco C, Pons F, Vicente JJ, Sanjuan A, Vanrell JA. Preventing postmenopausal bone loss with ossein-hydroxyapatite compounds. Results of a two-year, prospective trial. J Reprod Med 1999; 44:601-5.

33. Ruml LA, Sakhaee K, Peterson R, Adams-Huet B, Pak CY. The effect of calcium citrate on bone density in the early and mid-postmenopausal period: a randomized placebo-controlled study. Am J Ther 1999; 6:303-11.

34. Fujita T, Fujii Y, Goto B, Miyauchi A, Takagi Y. Peripheral computed tomography (pQCT) detected short-term effect of AAACa (heated oyster shell with heated algal ingredient HAI): A double-blind comparison with CaCO3 and placebo. J Bone Miner Metab 2000; 18:212-5.

35. Peacock M, Liu G, Carey M, McClintock R, Ambrosius W, Hui S, et al. Effect of calcium or 25OH vitamin D3 dietary supplementation on bone loss at the hip in men and women over the age of 60. J Clin Endocrinol Metab 2000; 85:3011-9.

36. Cleghorn DB, O'Loughlin PD, Schroeder BJ, Nordin BE. An open, crossover trial of calcium-fortified milk in prevention of early postmenopausal bone loss. Med J Aust 2001; 175:242-5.

37. Lau EM, Woo J, Lam V, Hong A. Milk supplementation of the diet of postmenopausal Chinese women on a low calcium intake retards bone loss. J Bone Miner Res 2001; 16:1704-9.

38. Son SM, Chun YN. Effect of oral therapy with alphacalcidol or calcium in Korean elderly women with osteopenia and low dietary calcium. Nutr Res 2001; 21:1347-55.

39. Chapuy MC, Pamphile R, Paris E, Kempf C, Schlichting M, Arnaud S, et al. Combined calcium and vitamin D3 supplementation in elderly women: confirmation of reversal of secondary hyperparathyroidism and hip fracture risk: the Decalyos II study. Osteoporos Int 2002; 13:257-64.

40. Chee WS, Suriah AR, Chan SP, Zaitun Y, Chan YM. The effect of milk supplementation on bone mineral density in postmenopausal Chinese women in Malaysia. Osteoporos Int 2003; 14:828-34.

41. Grados F, Brazier M, Kamel S, Duver S, Heurtebize N, Maamer M, et al. Effects on bone mineral density of calcium and vitamin D supplementation in elderly women with vitamin D deficiency. Joint Bone Spine 2003; 70:203-8.

42. Albertazzi P, Steel SA, Howarth EM, Purdie DW. Comparison of the effects of two different types of calcium supplementation on markers of bone metabolism in a postmenopausal osteopenic population with low calcium intake: a double-blind placebo-controlled trial. Climacteric 2004; 7:33-40.

43. Avenell A, Grant AM, McGee M, McPherson G, Campbell MK, McGee MA, et al. The effects of an open design on trial participant recruitment, compliance and retention--a randomized controlled trial comparison with a blinded, placebo-controlled design. Clin Trials 2004; 1:490-8.

44. Doetsch AM, Faber J, Lynnerup N, Watjen I, Bliddal H, Danneskiold-Samsoe B. The effect of calcium and vitamin D3 supplementation on the healing of the proximal humerus fracture: a randomized placebo-controlled study. Calcif Tissue Int 2004; 75:183-8.

45. Fujita T, Ohue M, Fujii Y, Miyauchi A, Takagi Y. Reappraisal of Katsuragi calcium study, a prospective, double-blind, placebo-controlled study of the effect of active absorbable algal calcium (AAACa) on vertebral deformity and fracture. J Bone Miner Metab 2004; 22:32-8.

46. Harwood RH, Sahota O, Gaynor K, Masud T, Hosking DJ, Nottingham Neck of Femur S. A randomised, controlled comparison of different calcium and vitamin D supplementation regimens in elderly women after hip fracture: The Nottingham Neck of Femur (NONOF) Study. Age Ageing 2004; 33:45-51.

47. Larsen ER, Mosekilde L, Foldspang A. Vitamin D and calcium supplementation prevents osteoporotic fractures in elderly community dwelling residents: a pragmatic population-based 3-year intervention study. J Bone Miner Res 2004; 19:370-8.

48. Meier C, Woitge HW, Witte K, Lemmer B, Seibel MJ. Supplementation with oral vitamin D3 and calcium during winter prevents seasonal bone loss: a randomized controlled open-label prospective trial. J Bone Miner Res 2004; 19:1221-30.

49. Coiro V, Volpi R, Zanardi G, Manfredi G, Varacca G, Saccani Jotti G. Effect of calcium supplementation as a high calcium mineral water on bone densitometry and laboratory parameters in menopausal women. [Italian]. Prog Nutr 2005; 7:192-200.

50. Grant AM, Avenell A, Campbell MK, McDonald AM, MacLennan GS, McPherson GC, et al. Oral vitamin D3 and calcium for secondary prevention of low-trauma fractures in elderly people (Randomised Evaluation of Calcium Or vitamin D, RECORD): a randomised placebo-controlled trial. Lancet 2005; 365:1621-8.

51. Porthouse J, Cockayne S, King C, Saxon L, Steele E, Aspray T, et al. Randomised controlled trial of calcium and supplementation with cholecalciferol (vitamin D3) for prevention of fractures in primary care. BMJ 2005; 330:1003.

52. Riedt CS, Cifuentes M, Stahl T, Chowdhury HA, Schlussel Y, Shapses SA. Overweight postmenopausal women lose bone with moderate weight reduction and 1 g/day calcium intake. J Bone Miner Res 2005; 20:455-63.

53. Daly RM, Brown M, Bass S, Kukuljan S, Nowson C. Calcium- and vitamin D3-fortified milk reduces bone loss at clinically relevant skeletal sites in older men: a 2-year randomized controlled trial. J Bone Miner Res 2006; 21:397-405.

54. Jackson RD, LaCroix AZ, Gass M, Wallace RB, Robbins J, Lewis CE, et al. Calcium plus vitamin D supplementation and the risk of fractures.[Erratum appears in N Engl J Med. 2006 Mar 9;354(10):1102]. N Engl J Med 2006; 354:669-83.

55. Prince RL, Devine A, Dhaliwal SS, Dick IM. Effects of calcium supplementation on clinical fracture and bone structure: results of a 5-year, double-blind, placebo-controlled trial in elderly women. Arch Intern Med 2006; 166:869-75.

56. Reid IR, Mason B, Horne A, Ames R, Reid HE, Bava U, et al. Randomized controlled trial of calcium in healthy older women. Am J Med 2006; 119:777-85.

57. Bolton-Smith C, McMurdo ME, Paterson CR, Mole PA, Harvey JM, Fenton ST, et al. Two-year randomized controlled trial of vitamin K1 (phylloquinone) and vitamin D3 plus calcium on the bone health of older women. J Bone Miner Res 2007; 22:509-19.

58. Bonnick S, Broy S, Kaiser F, Teutsch C, Rosenberg E, DeLucca P, et al. Treatment with alendronate plus calcium, alendronate alone, or calcium alone for postmenopausal low bone mineral density. Curr Med Res Opin 2007; 23:1341-9.

59. Hitz MF, Jensen JE, Eskildsen PC. Bone mineral density and bone markers in patients with a recent low-energy fracture: effect of 1 y of treatment with calcium and vitamin D. Am J Clin Nutr 2007; 86:251-9.

60. Manios Y, Moschonis G, Trovas G, Lyritis GP. Changes in biochemical indexes of bone metabolism and bone mineral density after a 12-mo dietary intervention program: the Postmenopausal Health Study. Am J Clin Nutr 2007; 86:781-9.

61. Bischoff-Ferrari HA, Rees JR, Grau MV, Barry E, Gui J, Baron JA. Effect of calcium supplementation on fracture risk: a double-blind randomized controlled trial. Am J Clin Nutr 2008; 87:1945-51.

62. Reid IR, Ames R, Mason B, Reid HE, Bacon CJ, Bolland MJ, et al. Randomized controlled trial of calcium supplementation in healthy, nonosteoporotic, older men. Arch Intern Med 2008; 168:2276-82.

63. Zhu K, Devine A, Dick IM, Wilson SG, Prince RL. Effects of calcium and vitamin D supplementation on hip bone mineral density and calcium-related analytes in elderly ambulatory Australian women: a five-year randomized controlled trial. J Clin Endocrinol Metab 2008; 93:743-9.

64. Kukuljan S, Nowson CA, Bass SL, Sanders K, Nicholson GC, Seibel MJ, et al. Effects of a multi-component exercise program and calcium-vitamin-D3-fortified milk on bone mineral density in older men: a randomised controlled trial. Osteoporos Int 2009; 20:1241-51.

65. Chailurkit LO, Saetung S, Thakkinstian A, Ongphiphadhanakul B, Rajatanavin R. Discrepant influence of vitamin D status on parathyroid hormone and bone mass after two years of calcium supplementation. Clin Endocrinol (Oxf) 2010; 73:167-72.

66. Karkkainen M, Tuppurainen M, Salovaara K, Sandini L, Rikkonen T, Sirola J, et al. Effect of calcium and vitamin D supplementation on bone mineral density in women aged 65-71years: a 3-year randomized population-based trial (OSTPRE-FPS). Osteoporos Int 2010; 21:2047-55.

67. Moschonis G, Katsaroli I, Lyritis GP, Manios Y. The effects of a 30-month dietary intervention on bone mineral density: the Postmenopausal Health Study. Br J Nutr 2010; 104:100-7.

68. Salovaara K, Tuppurainen M, Karkkainen M, Rikkonen T, Sandini L, Sirola J, et al. Effect of vitamin D(3) and calcium on fracture risk in 65- to 71-year-old women: a population-based 3-year randomized, controlled trial--the OSTPRE-FPS. J Bone Miner Res 2010; 25:1487-95.

69. Kukuljan S, Nowson CA, Sanders KM, Nicholson GC, Seibel MJ, Salmon J, et al. Independent and combined effects of calcium-vitamin D3 and exercise on bone structure and strength in older men: an 18-month factorial design randomized controlled trial. J Clin Endocrinol Metab 2011; 96:955-63.

70. Gui JC, Brasic JR, Liu XD, Gong GY, Zhang GM, Liu CJ, et al. Bone mineral density in postmenopausal Chinese women treated with calcium fortification in soymilk and cow's milk. Osteoporos Int 2012; 23:1563-70.

71. Nakamura K, Saito T, Kobayashi R, Oshiki R, Kitamura K, Oyama M, et al. Effect of low-dose calcium supplements on bone loss in perimenopausal and postmenopausal Asian women: a randomized controlled trial. J Bone Miner Res 2012; 27:2264-70.

72. Sambrook PN, Cameron ID, Chen JS, Cumming RG, Durvasula S, Herrmann M, et al. Does increased sunlight exposure work as a strategy to improve vitamin D status in the elderly: a cluster randomised controlled trial. Osteoporos Int 2012; 23:615-24.

73. Prentice RL, Pettinger MB, Jackson RD, Wactawski-Wende J, Lacroix AZ, Anderson GL, et al. Health risks and benefits from calcium and vitamin D supplementation: Women's Health Initiative clinical trial and cohort study. Osteoporos Int 2013; 24:567-80.

74. Rajatanavin R, Chailurkit L, Saetung S, Thakkinstian A, Nimitphong H. The efficacy of calcium supplementation alone in elderly Thai women over a 2-year period: a randomized controlled trial. Osteoporos Int 2013; 24:2871-7.

75. Wootton R, Brereton PJ, Clark MB, Hesp R, Hodkinson HM, Klenerman L, et al. Fractured neck of femur in the elderly: an attempt to identify patients at risk. Clin Sci (Lond) 1979; 57:93-101.

76. Paganini-Hill A, Ross RK, Gerkins VR, Henderson BE, Arthur M, Mack TM. Menopausal estrogen therapy and hip fractures. Ann Intern Med 1981; 95:28-31.

77. Riggs BL, Seeman E, Hodgson SF, Taves DR, O'Fallon WM. Effect of the fluoride/calcium regimen on vertebral fracture occurrence in postmenopausal osteoporosis. Comparison with conventional therapy. N Engl J Med 1982; 306:446-50.

78. Aloia JF, Cohn SH, Vaswani A, Yeh JK, Yuen K, Ellis K. Risk factors for postmenopausal osteoporosis. Am J Med 1985; 78:95-100.

79. Sandler RB, Slemenda CW, LaPorte RE, Cauley JA, Schramm MM, Barresi ML, et al. Postmenopausal bone density and milk consumption in childhood and adolescence. Am J Clin Nutr 1985; 42:270-4.

80. Sowers MR, Wallace RB, Lemke JH. Correlates of mid-radius bone density among postmenopausal women: a community study. Am J Clin Nutr 1985; 41:1045-53.

81. Yano K, Heilbrun LK, Wasnich RD, Hankin JH, Vogel JM. The relationship between diet and bone mineral content of multiple skeletal sites in elderly Japanese-American men and women living in Hawaii. Am J Clin Nutr 1985; 42:877-88.

82. Freudenheim JL, Johnson NE, Smith EL. Relationships between usual nutrient intake and bone-mineral content of women 35-65 years of age: longitudinal and cross-sectional analysis. Am J Clin Nutr 1986; 44:863-76.

83. Dawson-Hughes B, Jacques P, Shipp C. Dietary calcium intake and bone loss from the spine in healthy postmenopausal women. Am J Clin Nutr 1987; 46:685-7.

84. Ettinger B, Genant HK, Cann CE. Postmenopausal bone loss is prevented by treatment with low-dosage estrogen with calcium. Ann Intern Med 1987; 106:40-5.

85. Lukert BP, Carey M, McCarty B, Tiemann S, Goodnight L, Helm M, et al. Influence of nutritional factors on calcium-regulating hormones and bone loss. Calcif Tissue Int 1987; 40:119-25.

86. Nordin BE, Polley KJ. Metabolic consequences of the menopause. A cross-sectional, longitudinal, and intervention study on 557 normal postmenopausal women. Calcif Tissue Int 1987; 41 Suppl 1:S1-59.

87. Riggs BL, Wahner HW, Melton LJ, 3rd, Richelson LS, Judd HL, O'Fallon WM. Dietary calcium intake and rates of bone loss in women. J Clin Invest 1987; 80:979-82.

88. Angus RM, Sambrook PN, Pocock NA, Eisman JA. Dietary intake and bone mineral density. Bone Miner 1988; 4:265-77.

89. Cooper C, Barker DJ, Wickham C. Physical activity, muscle strength, and calcium intake in fracture of the proximal femur in Britain. BMJ 1988; 297:1443-6.

90. Holbrook TL, Barrett-Connor E, Wingard DL. Dietary calcium and risk of hip fracture: 14-year prospective population study. Lancet 1988; 2:1046-9.

91. Lau E, Donnan S, Barker DJ, Cooper C. Physical activity and calcium intake in fracture of the proximal femur in Hong Kong. BMJ 1988; 297:1441-3.

92. Stevenson JC, Whitehead MI, Padwick M, Endacott JA, Sutton C, Banks LM, et al. Dietary intake of calcium and postmenopausal bone loss. BMJ 1988; 297:15-7.

93. Tylavsky FA, Anderson JJ. Dietary factors in bone health of elderly lactoovovegetarian and omnivorous women. Am J Clin Nutr 1988; 48:842-9.

94. Cimino PM, Brinker MR, Cook SD, Harding AF. The effects of calcium on bone mineral density in postmenopausal women with colles fractures. J La State Med Soc 1989; 141:24-9.

95. Kleerekoper M, Peterson E, Nelson D, Tilley B, Phillips E, Schork MA, et al. Identification of women at risk for developing postmenopausal osteoporosis with vertebral fractures: role of history and single photon absorptiometry. Bone Miner 1989; 7:171-86.

96. Stevenson JC, Lees B, Devenport M, Cust MP, Ganger KF. Determinants of bone density in normal women: risk factors for future osteoporosis? BMJ 1989; 298:924-8.

97. Wickham CA, Walsh K, Cooper C, Barker DJ, Margetts BM, Morris J, et al. Dietary calcium, physical activity, and risk of hip fracture: a prospective study. BMJ 1989; 299:889-92.

98. Lutz J, Tesar R. Mother-daughter pairs: spinal and femoral bone densities and dietary intakes. Am J Clin Nutr 1990; 52:872-7.

99. Slemenda CW, Hui SL, Longcope C, Wellman H, Johnston CC, Jr. Predictors of bone mass in perimenopausal women. A prospective study of clinical data using photon absorptiometry. Ann Intern Med 1990; 112:96-101.

100. van Beresteijn EC, van 't Hof MA, Schaafsma G, de Waard H, Duursma SA. Habitual dietary calcium intake and cortical bone loss in perimenopausal women: a longitudinal study. Calcif Tissue Int 1990; 47:338-44.

101. van Beresteijn EC, van't Hof MA, de Waard H, Raymakers JA, Duursma SA. Relation of axial bone mass to habitual calcium intake and to cortical bone loss in healthy early postmenopausal women. Bone 1990; 11:7-13.

102. Andon MB, Smith KT, Bracker M, Sartoris D, Saltman P, Strause L. Spinal bone density and calcium intake in healthy postmenopausal women. Am J Clin Nutr 1991; 54:927-9.

103. Chi I, Pun KK. Dietary calcium intake and other risk factors: study of the fractured patients in Hong Kong. J Nutr Elder 1991; 10:73-87.

104. Hansen MA, Overgaard K, Riis BJ, Christiansen C. Potential risk factors for development of postmenopausal osteoporosis--examined over a 12-year period. Osteoporos Int 1991; 1:95-102.

105. Lacey JM, Anderson JJ, Fujita T, Yoshimoto Y, Fukase M, Tsuchie S, et al. Correlates of cortical bone mass among premenopausal and postmenopausal Japanese women. J Bone Miner Res 1991; 6:651-9.

106. Paganini-Hill A, Chao A, Ross RK, Henderson BE. Exercise and other factors in the prevention of hip fracture: the Leisure World study. Epidemiology 1991; 2:16-25.

107. Wheadon M, Goulding A, Barbezat GO, Campbell AJ. Lactose malabsorption and calcium intake as risk factors for osteoporosis in elderly New Zealand women. N Z Med J 1991; 104:417-9.

108. Buchs B, Rizzoli R, Slosman D, Nydegger V, Bonjour JP. [Bone mineral density of the lumbar spine, the femoral neck and femoral diaphysis in a Genevan population sample]. Schweiz Med Wochenschr 1992; 122:1129-36.

109. Howard G, Andon M, Bracker M, Saltman P, Strause L. Low serum copper, a risk factor additional to low dietary calcium in postmenopausal bone loss. J Trace Elem Exp Med 1992; 5:23-31.

110. Kanis JA, Johnell O, Gullberg B, Allander E, Dilsen G, Gennari C, et al. Evidence for efficacy of drugs affecting bone metabolism in preventing hip fracture. BMJ 1992; 305:1124-8.

111. Kelsey JL, Browner WS, Seeley DG, Nevitt MC, Cummings SR. Risk factors for fractures of the distal forearm and proximal humerus. The Study of Osteoporotic Fractures Research Group. Am J Epidemiol 1992; 135:477-89.

112. Kreiger N, Gross A, Hunter G. Dietary factors and fracture in postmenopausal women: a case-control study. Int J Epidemiol 1992; 21:953-8.

113. Nieves JW, Grisso JA, Kelsey JL. A case-control study of hip fracture: evaluation of selected dietary variables and teenage physical activity. Osteoporos Int 1992; 2:122-7.

114. Reid IR, Ames R, Evans MC, Sharpe S, Gamble G, France JT, et al. Determinants of total body and regional bone mineral density in normal postmenopausal women--a key role for fat mass. J Clin Endocrinol Metab 1992; 75:45-51.

115. Spector TD, Edwards AC, Thompson PW. Use of a risk factor and dietary calcium questionnaire in predicting bone density and subsequent bone loss at the menopause. Ann Rheum Dis 1992; 51:1252-3.

116. Bauer DC, Browner WS, Cauley JA, Orwoll ES, Scott JC, Black DM, et al. Factors associated with appendicular bone mass in older women. The Study of Osteoporotic Fractures Research Group. Ann Intern Med 1993; 118:657-65.

117. Hernandez-Avila M, Stampfer MJ, Ravnikar VA, Willett WC, Schiff I, Francis M, et al. Caffeine and other predictors of bone density among pre- and perimenopausal women. Epidemiology 1993; 4:128-34.

118. Hu JF, Zhao XH, Jia JB, Parpia B, Campbell TC. Dietary calcium and bone density among middle-aged and elderly women in China. Am J Clin Nutr 1993; 58:219-27.

119. Jaglal SB, Kreiger N, Darlington G. Past and recent physical activity and risk of hip fracture. Am J Epidemiol 1993; 138:107-18.

120. Jonsson B, Gardsell P, Johnell O, Sernbo I, Gullberg B. Life-style and different fracture prevalence: a cross-sectional comparative population-based study. Calcif Tissue Int 1993; 52:425-33.

121. Lau EM, Cooper C. Epidemiology and prevention of osteoporosis in urbanized Asian populations. Osteoporos Int 1993; 3 Suppl 1:23-6.

122. Looker AC, Harris TB, Madans JH, Sempos CT. Dietary calcium and hip fracture risk: the NHANES I Epidemiologic Follow-Up Study. Osteoporos Int 1993; 3:177-84.

123. Nordin BEC, Cleghorn DB, Chatterton BE, Morris HA, Need AG. A 5-year longitudinal study of forearm bone mass in 307 postmenopausal women. J Bone Miner Res 1993; 8:1427-32.

124. Ooms ME, Lips P, Van Lingen A, Valkenburg HA. Determinants of bone mineral density and risk factors for osteoporosis in healthy elderly women. J Bone Miner Res 1993; 8:669-75.

125. Perez Cano R, Galan Galan F, Dilsen G. Risk factors for hip fracture in Spanish and Turkish women. Bone 1993; 14 Suppl 1:S69-72.

126. Prince R, Dick I, Devine A, Kerr D, Criddle RA, Price R, et al. Importance of bone resorption in the determination of bone density in women more than 10 years past the menopause. J Bone Miner Res 1993; 8:1273-9.

127. Ribot C, Tremollieres F, Pouilles JM, Albarede JL, Mansat M, Utheza G, et al. Risk factors for hip fracture. MEDOS study: results of the Toulouse Centre. Bone 1993; 14 Suppl 1:S77-80.

128. Shaw CK. An epidemiologic study of osteoporosis in Taiwan. Ann Epidemiol 1993; 3:264-71.

129. Sowers MR, Clark MK, Jannausch ML, Wallace RB. Body size, estrogen use and thiazide diuretic use affect 5-year radial bone loss in postmenopausal women. Osteoporos Int 1993; 3:314-21.

130. Stracke H, Renner E, Knie G, Leidig G, Minne H, Federlin K. Osteoporosis and bone metabolic parameters in dependence upon calcium intake through milk and milk products. Eur J Clin Nutr 1993; 47:617-22.

131. Wyshak G. Dietary animal fat intake, calcium intake, and bone fractures in women 50 years and older. J Womens Health 1993; 2:329-34.

132. Cumming RG, Klineberg RJ. Case-control study of risk factors for hip fractures in the elderly. Am J Epidemiol 1994; 139:493-503.

133. Gilfillan CP, Silberberg S, Scrivenor P, Griffiths RC, McCloud PI, Burger HG. Determinants of forearm mineral density and its correlation with fracture history in women. Maturitas 1994; 20:199-208.

134. Hu JF, Zhao XH, Parpia B, Chen JS, Campbell TC. Assessment of a modified household food weighing method in a study of bone health in China. Eur J Clin Nutr 1994; 48:442-52.

135. Kroger H, Tuppurainen M, Honkanen R, Alhava E, Saarikoski S. Bone mineral density and risk factors for osteoporosis--a population-based study of 1600 perimenopausal women. Calcif Tissue Int 1994; 55:1-7.

136. Murphy S, Khaw KT, May H, Compston JE. Milk consumption and bone mineral density in middle aged and elderly women. BMJ 1994; 308:939-41.

137. Nguyen TV, Kelly PJ, Sambrook PN, Gilbert C, Pocock NA, Eisman JA. Lifestyle factors and bone density in the elderly: implications for osteoporosis prevention. J Bone Miner Res 1994; 9:1339-46.

138. Reid IR, Ames RW, Evans MC, Sharpe SJ, Gamble GD. Determinants of the rate of bone loss in normal postmenopausal women. J Clin Endocrinol Metab 1994; 79:950-4.

139. Soroko S, Holbrook TL, Edelstein S, Barrett-Connor E. Lifetime milk consumption and bone mineral density in older women. Am J Public Health 1994; 84:1319-22.

140. Tranquilli AL, Lucino E, Garzetti GG, Romanini C. Calcium, phosphorus and magnesium intakes correlate with bone mineral content in postmenopausal women. Gynecol Endocrinol 1994; 8:55-8.

141. Cummings SR, Nevitt MC, Browner WS, Stone K, Fox KM, Ensrud KE, et al. Risk factors for hip fracture in white women. Study of Osteoporotic Fractures Research Group. N Engl J Med 1995; 332:767-73.

142. Davis JW, Ross PD, Johnson NE, Wasnich RD. Estrogen and calcium supplement use among Japanese-American women: effects upon bone loss when used singly and in combination. Bone 1995; 17:369-73.

143. Devine A, Criddle RA, Dick IM, Kerr DA, Prince RL. A longitudinal study of the effect of sodium and calcium intakes on regional bone density in postmenopausal women. Am J Clin Nutr 1995; 62:740-5.

144. Glynn NW, Meilahn EN, Charron M, Anderson SJ, Kuller LH, Cauley JA. Determinants of bone mineral density in older men. J Bone Miner Res 1995; 10:1769-77.

145. Greendale GA, Wells B, Barrett-Connor E, Marcus R, Bush T. Lifestyle factors and bone mineral density: The Postmenopausal Estrogen/Progestins Intervention Study. J Womens Health 1995; 4:231-45.

146. Ho SC, Chan SS, Woo J, Leung PC, Lau J. Determinants of bone mass in the Chinese old-old population. Osteoporos Int 1995; 5:161-6.

147. Holbrook TL, Barrett-Connor E. An 18-year prospective study of dietary calcium and bone mineral density in the hip. Calcif Tissue Int 1995; 56:364-7.

148. Johnell O, Gullberg B, Kanis JA, Allander E, Elffors L, Dequeker J, et al. Risk factors for hip fracture in European women: the MEDOS Study. Mediterranean Osteoporosis Study. J Bone Miner Res 1995; 10:1802-15.

149. Meyer HE, Henriksen C, Falch JA, Pedersen JI, Tverdal A. Risk factors for hip fracture in a high incidence area: a case-control study from Oslo, Norway. Osteoporos Int 1995; 5:239-46.

150. Michaelsson K, Holmberg L, Mallmin H, Sorensen S, Wolk A, Bergstrom R, et al. Diet and hip fracture risk: a case-control study. Study Group of the Multiple Risk Survey on Swedish Women for Eating Assessment. Int J Epidemiol 1995; 24:771-82.

151. Tavani A, Negri E, La Vecchia C. Calcium, dairy products, and the risk of hip fracture in women in northern Italy. Epidemiology 1995; 6:554-7.

152. Vico L, Pouget JF, Calmels P, Chatard JC, Rehailia M, Minaire P, et al. The relations between physical ability and bone mass in women aged over 65 years. J Bone Miner Res 1995; 10:374-83.

153. Ward JA, Lord SR, Williams P, Anstey K, Zivanovic E. Physiologic, health and lifestyle factors associated with femoral neck bone density in older women. Bone 1995; 16:373s-8s.

154. Bendavid EJ, Shan J, Barrett-Connor E. Factors associated with bone mineral density in middle-aged men. J Bone Miner Res 1996; 11:1185-90.

155. Chan HH, Lau EM, Woo J, Lin F, Sham A, Leung PC. Dietary calcium intake, physical activity and the risk of vertebral fracture in Chinese. Osteoporos Int 1996; 6:228-32.

156. Clemente PA, Armengol R, Francesch A, Vila J, Domenech B. Osteoporosis and calcium ingest. [Spanish]. Progresos en Obstetricia y Ginecologia 1996; 39:289-92.

157. Cooper C, Atkinson EJ, Hensrud DD, Wahner HW, O'Fallon WM, Riggs BL, et al. Dietary protein intake and bone mass in women. Calcif Tissue Int 1996; 58:320-5.

158. Cosman F, Nieves J, Wilkinson C, Schnering D, Shen V, Lindsay R. Bone density change and biochemical indices of skeletal turnover. Calcif Tissue Int 1996; 58:236-43.

159. Honkanen R, Pulkkinen P, Jarvinen R, Kroger H, Lindstedt K, Tuppurainen M, et al. Does lactose intolerance predispose to low bone density? A population-based study of perimenopausal Finnish women. Bone 1996; 19:23-8.

160. Hoover PA, Webber CE, Beaumont LF, Blake JM. Postmenopausal bone mineral density: relationship to calcium intake, calcium absorption, residual estrogen, body composition, and physical activity. Can J Physiol Pharmacol 1996; 74:911-7.

161. Huang Z, Himes JH, McGovern PG. Nutrition and subsequent hip fracture risk among a national cohort of white women. Am J Epidemiol 1996; 144:124-34.

162. Nguyen TV, Eisman JA, Kelly PJ, Sambrook PN. Risk factors for osteoporotic fractures in elderly men. Am J Epidemiol 1996; 144:255-63.

163. O'Neill TW, Marsden D, Adams JE, Silman AJ. Risk factors, falls, and fracture of the distal forearm in Manchester, UK. J Epidemiol Community Health 1996; 50:288-92.

164. Orwoll ES, Bauer DC, Vogt TM, Fox KM. Axial bone mass in older women. Study of Osteoporotic Fractures Research Group. Ann Intern Med 1996; 124:187-96.

165. Pouilles JM, Tremollieres F, Ribot C. [Vertebral bone loss in perimenopause. Results of a 7-year longitudinal study]. Presse Med 1996; 25:277-80.

166. Sone T, Miyake M, Takeda N, Tomomitsu T, Otsuka N, Fukunaga M. Influence of exercise and degenerative vertebral changes on BMD: a cross-sectional study in Japanese men. Gerontology 1996; 42 Suppl 1:57-66.

167. Ueda A, Yoshimura N, Morioka S, Kasamatsu T, Kinoshita H, Hashimoto T. [A population based study on factors related to bone mineral density in Wakayama Prefecture]. Nihon Koshu Eisei Zasshi 1996; 43:50-61.

168. Ulrich CM, Georgiou CC, Snow-Harter CM, Gillis DE. Bone mineral density in mother-daughter pairs: relations to lifetime exercise, lifetime milk consumption, and calcium supplements. Am J Clin Nutr 1996; 63:72-9.

169. Valdivia G, Giaconi J, Arteaga E, Pumarino H, Gajardo H, Villarroel L. [Hip fracture: a case-control study in the metropolitan region I]. Rev Med Chil 1996; 124:189-97.

170. Cumming RG, Cummings SR, Nevitt MC, Scott J, Ensrud KE, Vogt TM, et al. Calcium intake and fracture risk: results from the study of osteoporotic fractures. Am J Epidemiol 1997; 145:926-34.

171. Earnshaw SA, Worley A, Hosking DJ. Current diet does not relate to bone mineral density after the menopause. The Nottingham Early Postmenopausal Intervention Cohort (EPIC) Study Group. Br J Nutr 1997; 78:65-72.

172. Fujiwara S, Kasagi F, Yamada M, Kodama K. Risk factors for hip fracture in a Japanese cohort. J Bone Miner Res 1997; 12:998-1004.

173. Meyer HE, Pedersen JI, Loken EB, Tverdal A. Dietary factors and the incidence of hip fracture in middle-aged Norwegians. A prospective study. Am J Epidemiol 1997; 145:117-23.

174. Michaelsson K, Bergstrom R, Holmberg L, Mallmin H, Wolk A, Ljunghall S. A high dietary calcium intake is needed for a positive effect on bone density in Swedish postmenopausal women. Osteoporos Int 1997; 7:155-61.

175. Owusu W, Willett WC, Feskanich D, Ascherio A, Spiegelman D, Colditz GA. Calcium intake and the incidence of forearm and hip fractures among men. J Nutr 1997; 127:1782-7.

176. Suleiman S, Nelson M, Li F, Buxton-Thomas M, Moniz C. Effect of calcium intake and physical activity level on bone mass and turnover in healthy, white, postmenopausal women. Am J Clin Nutr 1997; 66:937-43.

177. Suzuki T, Yoshida H, Hashimoto T, Yoshimura N, Fujiwara S, Fukunaga M, et al. Case-control study of risk factors for hip fractures in the Japanese elderly by a Mediterranean Osteoporosis Study (MEDOS) questionnaire. Bone 1997; 21:461-7.

178. Wang MC, Luz Villa M, Marcus R, Kelsey JL. Associations of vitamin C, calcium and protein with bone mass in postmenopausal Mexican American women. Osteoporos Int 1997; 7:533-8.

179. Burger H, de Laet CE, van Daele PL, Weel AE, Witteman JC, Hofman A, et al. Risk factors for increased bone loss in an elderly population: the Rotterdam Study. Am J Epidemiol 1998; 147:871-9.

180. Clark P, de la Pena F, Gomez Garcia F, Orozco JA, Tugwell P. Risk factors for osteoporotic hip fractures in Mexicans. Arch Med Res 1998; 29:253-7.

181. Hosking DJ, Ross PD, Thompson DE, Wasnich RD, McClung M, Bjarnason NH, et al. Evidence that increased calcium intake does not prevent early postmenopausal bone loss. Clin Ther 1998; 20:933-44.

182. Lau EM, Kwok T, Woo J, Ho SC. Bone mineral density in Chinese elderly female vegetarians, vegans, lacto-vegetarians and omnivores. Eur J Clin Nutr 1998; 52:60-4.

183. Masaryk P, Lunt M, Benevolenskaya L, Cannata J, Dequeker J, Dohenhof C, et al. Effects of menstrual history and use of medications on bone mineral density: the EVOS Study. Calcif Tissue Int 1998; 63:271-6.

184. Mosquera MT, Maurel DL, Pavon S, Arregui A, Moreno C, Vazquez J. [Incidence and risk factors in fractures of the proximal femur due to osteoporosis]. Rev Panam Salud Publica 1998; 3:211-9.

185. Mussolino ME, Looker AC, Madans JH, Langlois JA, Orwoll ES. Risk factors for hip fracture in white men: the NHANES I Epidemiologic Follow-up Study. J Bone Miner Res 1998; 13:918-24.

186. Rodriguez JA, Novik V. [Calcium intake and bone density in menopause. Data of a sample of Chilean women followed-up for 5 years with calcium supplementation]. Rev Med Chil 1998; 126:145-50.

187. Turner LW, Fu Q, Taylor JE, Wang MQ. Osteoporotic fracture among older U.S. women: risk factors quantified. J Aging Health 1998; 10:372-91.

188. Uusi-Rasi K, Sievanen H, Vuori I, Pasanen M, Heinonen A, Oja P. Associations of physical activity and calcium intake with bone mass and size in healthy women at different ages. J Bone Miner Res 1998; 13:133-42.

189. Aptel I, Cance-Rouzaud A, Grandjean H. Association between calcium ingested from drinking water and femoral bone density in elderly women: evidence from the EPIDOS cohort. J Bone Miner Res 1999; 14:829-33.

190. Bernad M, Jaramillo G, Aguado P, del Campo T, Coya J, Martin Mola E, et al. [Polymorphism of the gene of vitamin D receptor and bone mineral density in postmenopausal women]. Med Clin (Barc) 1999; 112:651-5.

191. Brot C, Jorgensen N, Madsen OR, Jensen LB, Sorensen OH. Relationships between bone mineral density, serum vitamin D metabolites and calcium:phosphorus intake in healthy perimenopausal women. J Intern Med 1999; 245:509-16.

192. Dennison E, Eastell R, Fall CH, Kellingray S, Wood PJ, Cooper C. Determinants of bone loss in elderly men and women: a prospective population-based study. Osteoporos Int 1999; 10:384-91.

193. Goulding A, Taylor RW, Keil D, Gold E, Lewis-Barned NJ, Williams SM. Lactose malabsorption and rate of bone loss in older women. Age Ageing 1999; 28:175-80.

194. Kanis J, Johnell O, Gullberg B, Allander E, Elffors L, Ranstam J, et al. Risk factors for hip fracture in men from southern Europe: the MEDOS study. Mediterranean Osteoporosis Study. Osteoporos Int 1999; 9:45-54.

195. Melhus H, Michaelsson K, Holmberg L, Wolk A, Ljunghall S. Smoking, antioxidant vitamins, and the risk of hip fracture. J Bone Miner Res 1999; 14:129-35.

196. Metz JA, Morris CD, Roberts LA, McClung MR, McCarron DA. Blood pressure and calcium intake are related to bone density in adult males. Br J Nutr 1999; 81:383-8.

197. Munger RG, Cerhan JR, Chiu BC. Prospective study of dietary protein intake and risk of hip fracture in postmenopausal women. Am J Clin Nutr 1999; 69:147-52.

198. Turner LW, Hunt S, Kendrick O, Eddy J. Dairy-product intake and hip fracture among older women: issues for health behavior. Psychol Rep 1999; 85:423-30.

199. Varenna M, Binelli L, Zucchi F, Ghiringhelli D, Gallazzi M, Sinigaglia L. Prevalence of osteoporosis by educational level in a cohort of postmenopausal women. Osteoporos Int 1999; 9:236-41.

200. Di Monaco M, Di Monaco R, Manca M, Cavanna A. Handgrip strength is an independent predictor of distal radius bone mineral density in postmenopausal women. Clin Rheumatol 2000; 19:473-6.

201. Dirschl DR, Piedrahita L, Henderson RC. Bone mineral density 6 years after a hip fracture: a prospective, longitudinal study. Bone 2000; 26:95-8.

202. Guthrie JR, Ebeling PR, Dennerstein L, Wark JD. Risk factors for osteoporosis: prevalence, change, and association with bone density. Medscape Womens Health 2000; 5:E2.

203. Hannan MT, Felson DT, Dawson-Hughes B, Tucker KL, Cupples LA, Wilson PW, et al. Risk factors for longitudinal bone loss in elderly men and women: the Framingham Osteoporosis Study. J Bone Miner Res 2000; 15:710-20.

204. Honkanen RJ, Honkanen K, Kroger H, Alhava E, Tuppurainen M, Saarikoski S. Risk factors for perimenopausal distal forearm fracture. Osteoporos Int 2000; 11:265-70.

205. Huo D, Li L. [A case - control study on risk factors for hip fracture in the middle - aged and elderly in Beijing]. Zhonghua Liu Xing Bing Xue Za Zhi 2000; 21:37-40.

206. Huopio J, Kroger H, Honkanen R, Saarikoski S, Alhava E. Risk factors for perimenopausal fractures: a prospective study. Osteoporos Int 2000; 11:219-27.

207. Huuskonen J, Vaisanen SB, Kroger H, Jurvelin C, Bouchard C, Alhava E, et al. Determinants of bone mineral density in middle aged men: a population-based study. Osteoporos Int 2000; 11:702-8.

208. Jaime PC, Marucci MDFN, De Oliveira Latorre MDRD, Tanaka T, Florindo AA, De Freitas Zerbini CA. Influence of dietetic calcium intake on the bone mineral density of men who are 50 year old and older. [Portuguese]. Rev Bras Reumatol 2000; 40:105-11.

209. Kato I, Toniolo P, Zeleniuch-Jacquotte A, Shore RE, Koenig KL, Akhmedkhanov A, et al. Diet, smoking and anthropometric indices and postmenopausal bone fractures: a prospective study. Int J Epidemiol 2000; 29:85-92.

210. Nguyen TV, Center JR, Eisman JA. Osteoporosis in elderly men and women: effects of dietary calcium, physical activity, and body mass index. J Bone Miner Res 2000; 15:322-31.

211. Orwoll ES, Bevan L, Phipps KR. Determinants of bone mineral density in older men. Osteoporos Int 2000; 11:815-21.

212. Picard D, Imbach A, Couturier M, Lepage R, Ste Marie LG. Longitudinal study of bone density and its determinants in women in peri- or early menopause. Calcif Tissue Int 2000; 67:356-60.

213. Jitapunkul S, Yuktananandana P, Parkpian V. Risk factors of hip fracture among Thai female patients. J Med Assoc Thai 2001; 84:1576-81.

214. Lau EM, Suriwongpaisal P, Lee JK, Das De S, Festin MR, Saw SM, et al. Risk factors for hip fracture in Asian men and women: the Asian osteoporosis study. J Bone Miner Res 2001; 16:572-80.

215. Lumbers M, New SA, Gibson S, Murphy MC. Nutritional status in elderly female hip fracture patients: comparison with an age-matched home living group attending day centres. Br J Nutr 2001; 85:733-40.

216. Lunt M, Masaryk P, Scheidt-Nave C, Nijs J, Poor G, Pols H, et al. The effects of lifestyle, dietary dairy intake and diabetes on bone density and vertebral deformity prevalence: the EVOS study. Osteoporos Int 2001; 12:688-98.

217. Nguyen TV, Center JR, Sambrook PN, Eisman JA. Risk factors for proximal humerus, forearm, and wrist fractures in elderly men and women: the Dubbo Osteoporosis Epidemiology Study. Am J Epidemiol 2001; 153:587-95.

218. Piaseu N, Komindr S, Chailurkit LO, Ongphiphadhanakul B, Chansirikarn S, Rajatanavin R. Differences in bone mineral density and lifestyle factors of postmenopausal women living in Bangkok and other provinces. J Med Assoc Thai 2001; 84:772-81.

219. Ramalho AC, Lazaretti-Castro M, Hauache O, Vieira JG, Takata E, Cafalli F, et al. Osteoporotic fractures of proximal femur: clinical and epidemiological features in a population of the city of Sao Paulo. Sao Paulo Med J 2001; 119:48-53.

220. Sasaki S, Yanagibori R. Association between current nutrient intakes and bone mineral density at calcaneus in pre- and postmenopausal Japanese women. J Nutr Sci Vitaminol (Tokyo) 2001; 47:289-94.

221. Tanaka T, Latorre MRDO, Jaime PC, Florindo AA, Pippa MGB, Zerbini CAF. Risk factors for proximal femur osteoporosis in men aged 50 years or older. Osteoporos Int 2001; 12:942-9.

222. Varenna M, Binelli L, Zucchi F, Ghiringhelli D, Sinigaglia L. Unbalanced diet to lower serum cholesterol level is a risk factor for postmenopausal osteoporosis and distal forearm fracture. Osteoporos Int 2001; 12:296-301.

223. Vestergaard P, Hermann AP, Gram J, Jensen LB, Eiken P, Abrahamsen B, et al. Evaluation of methods for prediction of bone mineral density by clinical and biochemical variables in perimenopausal women. Maturitas 2001; 40:211-20.

224. Blain H, Vuillemin A, Guillemin F, Durant R, Hanesse B, de Talance N, et al. Serum leptin level is a predictor of bone mineral density in postmenopausal women. J Clin Endocrinol Metab 2002; 87:1030-5.

225. Dargent-Molina P, Douchin MN, Cormier C, Meunier PJ, Breart G, Group ES. Use of clinical risk factors in elderly women with low bone mineral density to identify women at higher risk of hip fracture: The EPIDOS prospective study. Osteoporos Int 2002; 13:593-9.

226. del Puente A, Esposito A, Savastano S, Carpinelli A, Postiglione L, Oriente P. Dietary calcium intake and serum vitamin D are major determinants of bone mass variations in women. A longitudinal study. Aging Clin Exp Res 2002; 14:382-8.

227. Ilich-Ernst J, Brownbill RA, Ludemann MA, Fu R. Critical factors for bone health in women across the age span: how important is muscle mass? Medscape Womens Health 2002; 7:2.

228. Lee SH, Dargent-Molina P, Breart G, Study EGEdlO. Risk factors for fractures of the proximal humerus: results from the EPIDOS prospective study. J Bone Miner Res 2002; 17:817-25.

229. Mendez RO, Gomez MA, Lopez AM, Gonzalez H, Wyatt CJ. Effects of calcium and phosphorus intake and excretion on bone density in postmenopausal women in Hermosillo, Mexico. Ann Nutr Metab 2002; 46:249-53.

230. Roman Garcia MM, Garces Puentes MV, Diaz Curiel M. Relationship between bone mineral density and fractures in postmenopausal women of the extremadura comunity. [Spanish]. Rev Esp de Enferm Metabol Oseas 2002; 11:135-9.

231. Wu F, Ames R, Clearwater J, Evans MC, Gamble G, Reid IR. Prospective 10-year study of the determinants of bone density and bone loss in normal postmenopausal women, including the effect of hormone replacement therapy. Clin Endocrinol (Oxf) 2002; 56:703-11.

232. Albrand G, Munoz F, Sornay-Rendu E, DuBoeuf F, Delmas PD. Independent predictors of all osteoporosis-related fractures in healthy postmenopausal women: the OFELY study. Bone 2003; 32:78-85.

233. Ballard JE, Wallace LS, Holiday DB, Herron C, Harrington LL, Mobbs KC, et al. Evaluation of Differences in Bone-Mineral Density in 51 Men Age 65-93 Years: A Cross-Sectional Study. J Aging Phys Act 2003; 11:470-86.

234. Feskanich D, Willett WC, Colditz GA. Calcium, vitamin D, milk consumption, and hip fractures: a prospective study among postmenopausal women. Am J Clin Nutr 2003; 77:504-11.

235. Ilich JZ, Brownbill RA, Tamborini L. Bone and nutrition in elderly women: protein, energy, and calcium as main determinants of bone mineral density.[Erratum appears in Eur J Clin Nutr. 2003 Jul;57(7):880]. Eur J Clin Nutr 2003; 57:554-65.

236. Knoke JD, Barrett-Connor E. Weight loss: a determinant of hip bone loss in older men and women. The Rancho Bernardo Study. Am J Epidemiol 2003; 158:1132-8.

237. Korpelainen R, Korpelainen J, Heikkinen J, Vaananen K, Keinanen-Kiukaanniemi S. Lifestyle factors are associated with osteoporosis in lean women but not in normal and overweight women: a population-based cohort study of 1222 women. Osteoporos Int 2003; 14:34-43.

238. MacInnis RJ, Cassar C, Nowson CA, Paton LM, Flicker L, Hopper JL, et al. Determinants of bone density in 30- to 65-year-old women: a co-twin study. J Bone Miner Res 2003; 18:1650-6.

239. Melton LJ, 3rd, Crowson CS, O'Fallon WM, Wahner HW, Riggs BL. Relative contributions of bone density, bone turnover, and clinical risk factors to long-term fracture prediction. J Bone Miner Res 2003; 18:312-8.

240. Michaelsson K, Melhus H, Bellocco R, Wolk A. Dietary calcium and vitamin D intake in relation to osteoporotic fracture risk. Bone 2003; 32:694-703.

241. Roy DK, O'Neill TW, Finn JD, Lunt M, Silman AJ, Felsenberg D, et al. Determinants of incident vertebral fracture in men and women: results from the European Prospective Osteoporosis Study (EPOS). Osteoporos Int 2003; 14:19-26.

242. Runyan SM, Stadler DD, Bainbridge CN, Miller SC, Moyer-Mileur LJ. Familial resemblance of bone mineralization, calcium intake, and physical activity in early-adolescent daughters, their mothers, and maternal grandmothers. J Am Diet Assoc 2003; 103:1320-5.

243. Sirola J, Kroger H, Honkanen R, Sandini L, Tuppurainen M, Jurvelin JS, et al. Smoking may impair the bone protective effects of nutritional calcium: a population-based approach. J Bone Miner Res 2003; 18:1036-42.

244. Sirola J, Kroger H, Sandini L, Tuppurainen M, Jurvelin JS, Saarikoski S, et al. Interaction of nutritional calcium and HRT in prevention of postmenopausal bone loss: a prospective study. Calcif Tissue Int 2003; 72:659-65.

245. Suzuki Y, Whiting SJ, Davison KS, Chilibeck PD. Total calcium intake is associated with cortical bone mineral density in a cohort of postmenopausal women not taking estrogen. J Nutr Health Aging 2003; 7:296-9.

246. Bakhireva LN, Barrett-Connor E, Kritz-Silverstein D, Morton DJ. Modifiable predictors of bone loss in older men: a prospective study. Am J Prev Med 2004; 26:436-42.

247. Bhattoa HP, Bettembuk P, Ganacharya S, Balogh A. Prevalence and seasonal variation of hypovitaminosis D and its relationship to bone metabolism in community dwelling postmenopausal Hungarian women. Osteoporos Int 2004; 15:447-51.

248. Chu SP, Kelsey JL, Keegan TH, Sternfeld B, Prill M, Quesenberry CP, et al. Risk factors for proximal humerus fracture. Am J Epidemiol 2004; 160:360-7.

249. Devine A, Dhaliwal SS, Dick IM, Bollerslev J, Prince RL. Physical activity and calcium consumption are important determinants of lower limb bone mass in older women. J Bone Miner Res 2004; 19:1634-9.

250. Hagino H, Fujiwara S, Nakashima E, Nanjo Y, Teshima R. Case-control study of risk factors for fractures of the distal radius and proximal humerus among the Japanese population. Osteoporos Int 2004; 15:226-30.

251. Ho SC, Chen YM, Woo JL, Lam SS. High habitual calcium intake attenuates bone loss in early postmenopausal Chinese women: an 18-month follow-up study. J Clin Endocrinol Metab 2004; 89:2166-70.

252. Kurabayashi T, Matsushita H, Kato N, Nagata H, Kikuchi M, Tomita M, et al. Effect of vitamin D receptor and estrogen receptor gene polymorphism on the relationship between dietary calcium and bone mineral density in Japanese women. J Bone Miner Metab 2004; 22:139-47.

253. Lopez-Caudana AE, Tellez-Rojo Solis MM, Hernandez-Avila M, Clark P, Juarez-Marquez SA, Lazcano-Ponce EC, et al. Predictors of bone mineral density in female workers in Morelos State, Mexico. Arch Med Res 2004; 35:172-80.

254. Luetters CM, Keegan TH, Sidney S, Quesenberry CP, Prill M, Sternfeld B, et al. Risk factors for foot fracture among individuals aged 45 years and older. Osteoporos Int 2004; 15:957-63.

255. Macdonald HM, New SA, Golden MH, Campbell MK, Reid DM. Nutritional associations with bone loss during the menopausal transition: evidence of a beneficial effect of calcium, alcohol, and fruit and vegetable nutrients and of a detrimental effect of fatty acids. Am J Clin Nutr 2004; 79:155-65.

256. McCabe LD, Martin BR, McCabe GP, Johnston CC, Weaver CM, Peacock M. Dairy intakes affect bone density in the elderly. Am J Clin Nutr 2004; 80:1066-74.

257. Pongchaiyakul C, Nguyen TV, Kosulwat V, Rojroongwasinkul N, Charoenkiatkul S, Eisman JA, et al. Effects of physical activity and dietary calcium intake on bone mineral density and osteoporosis risk in a rural Thai population. Osteoporos Int 2004; 15:807-13.

258. van der Klift M, de Laet CE, McCloskey EV, Johnell O, Kanis JA, Hofman A, et al. Risk factors for incident vertebral fractures in men and women: the Rotterdam Study. J Bone Miner Res 2004; 19:1172-80.

259. Wengreen HJ, Munger RG, West NA, Cutler DR, Corcoran CD, Zhang J, et al. Dietary protein intake and risk of osteoporotic hip fracture in elderly residents of Utah. J Bone Miner Res 2004; 19:537-45.

260. Yarbrough MM, Williams DP, Allen MM. Risk factors associated with osteoporosis in Hispanic women. J Women Aging 2004; 16:91-104.

261. Cauley JA, Fullman RL, Stone KL, Zmuda JM, Bauer DC, Barrett-Connor E, et al. Factors associated with the lumbar spine and proximal femur bone mineral density in older men. Osteoporos Int 2005; 16:1525-37.

262. Chen YP, Cai DH, Liu XQ, Yang L, Tang LL, Yang YL, et al. Risk factors of osteoporosis among middle-aged and elderly people in community. [Chinese]. Chin J Clin Rehab 2005; 9:156-7.

263. Cheung EY, Ho AY, Lam KF, Tam S, Kung AW. Determinants of bone mineral density in Chinese men. Osteoporos Int 2005; 16:1481-6.

264. Cussler EC, Going SB, Houtkooper LB, Stanford VA, Blew RM, Flint-Wagner HG, et al. Exercise frequency and calcium intake predict 4-year bone changes in postmenopausal women. Osteoporos Int 2005; 16:2129-41.

265. Hassa H, Tanir HM, Senses T, Oge T, Sahin-Mutlu F. Related factors in bone mineral density of lumbal and femur in natural postmenopausal women. Arch Gynecol Obstet 2005; 273:86-9.

266. Kanis JA, Johansson H, Oden A, De Laet C, Johnell O, Eisman JA, et al. A meta-analysis of milk intake and fracture risk: low utility for case finding. Osteoporos Int 2005; 16:799-804.

267. Kelsey JL, Prill MM, Keegan TH, Tanner HE, Bernstein AL, Quesenberry CP, Jr., et al. Reducing the risk for distal forearm fracture: preserve bone mass, slow down, and don't fall! Osteoporos Int 2005; 16:681-90.

268. Kung AW, Ho AY, Ross PD, Reginster JY. Development of a clinical assessment tool in identifying Asian men with low bone mineral density and comparison of its usefulness to quantitative bone ultrasound. Osteoporos Int 2005; 16:849-55.

269. Lau HH, Ng MY, Ho AY, Luk KD, Kung AW. Genetic and environmental determinants of bone mineral density in Chinese women. Bone 2005; 36:700-9.

270. Meier C, Nguyen TV, Center JR, Seibel MJ, Eisman JA. Bone resorption and osteoporotic fractures in elderly men: the dubbo osteoporosis epidemiology study. J Bone Miner Res 2005; 20:579-87.

271. Morita A, Iki M, Dohi Y, Ikeda Y, Kagamimori S, Kagawa Y, et al. Effects of the Cdx-2 polymorphism of the vitamin D receptor gene and lifestyle factors on bone mineral density in a representative sample of Japanese women: the Japanese Population-based Osteoporosis (JPOS) Study. Calcif Tissue Int 2005; 77:339-47.

272. Naves M, Diaz-Lopez JB, Gomez C, Rodriguez-Rebollar A, Serrano-Arias M, Cannata-Andia JB. Prevalence of osteoporosis in men and determinants of changes in bone mass in a non-selected Spanish population. Osteoporos Int 2005; 16:603-9.

273. Ofluoglu D, Gunduz OH, Bekirolu N, Kul-Panza E, Akyuz G. A method for determining the grade of osteoporosis based on risk factors in postmenopausal women. Clin Rheumatol 2005; 24:606-11.

274. Papaioannou A, Joseph L, Ioannidis G, Berger C, Anastassiades T, Brown JP, et al. Risk factors associated with incident clinical vertebral and nonvertebral fractures in postmenopausal women: the Canadian Multicentre Osteoporosis Study (CaMos). Osteoporos Int 2005; 16:568-78.

275. Romero AA, Duarte-Gardea M, Ortiz M, Labrado C, Noe M. Bone mineral density and body mass index of Mexican American Women. Hisp Health Care Int 2005; 3:9-14.

276. Uusi-Rasi K, Sievanen H, Heinonen A, Beck TJ, Vuori I. Determinants of changes in bone mass and femoral neck structure, and physical performance after menopause: a 9-year follow-up of initially peri-menopausal women. Osteoporos Int 2005; 16:616-22.

277. Abraham R, Walton J, Russell L, Wolman R, Wardley-Smith B, Green JR, et al. Dietary determinants of post-menopausal bone loss at the lumbar spine: a possible beneficial effect of iron. Osteoporos Int 2006; 17:1165-73.

278. Babbar RK, Handa AB, Lo CM, Guttmacher SJ, Shindledecker R, Chung W, et al. Bone health of immigrant Chinese women living in New York City. J Community Health 2006; 31:7-23.

279. Dong J, Huang ZW, Piao JH, Gong J. [Association of bone mineral density with gene polymorphisms and environmental factors in Chinese postmenopausal women]. Wei Sheng Yan Jiu 2006; 35:196-200.

280. Jaime PC, Latorre Mdo R, Florindo AA, Tanaka T, Zerbini CA. Dietary intake of Brazilian black and white men and its relationship to the bone mineral density of the femoral neck. Sao Paulo Med J 2006; 124:267-70.

281. Lau EM, Leung PC, Kwok T, Woo J, Lynn H, Orwoll E, et al. The determinants of bone mineral density in Chinese men--results from Mr. Os (Hong Kong), the first cohort study on osteoporosis in Asian men. Osteoporos Int 2006; 17:297-303.

282. Michaelsson K, Wolk A, Jacobsson A, Kindmark A, Grundberg E, Stiger F, et al. The positive effect of dietary vitamin D intake on bone mineral density in men is modulated by the polyadenosine repeat polymorphism of the vitamin D receptor. Bone 2006; 39:1343-51.

283. Nakamura K, Saito T, Nishiwaki T, Ueno K, Nashimoto M, Okuda Y, et al. Correlations between bone mineral density and demographic, lifestyle, and biochemical variables in community-dwelling Japanese women 69 years of age and over. Osteoporos Int 2006; 17:1202-7.

284. van Geel ACM, Geusens PP, Nagtzaam IF, Schreurs CMJR, van der Voort DJM, Rinkens PELM, et al. Timing and risk factors for clinical fractures among postmenopausal women: A 5-year prospective study. BMC Med 2006; 4.

285. Wang MC, Dixon LB. Socioeconomic influences on bone health in postmenopausal women: findings from NHANES III, 1988-1994. Osteoporos Int 2006; 17:91-8.

286. Xu L, Phillips M, D'Este C, Dibley M, Porteous J, Attia J. Diet, activity, and other lifestyle risk factors for forearm fracture in postmenopausal women in China: a case-control study. Menopause 2006; 13:102-10.

287. Angbratt M, Timpka T, Blomberg C, Kronhed AC, Waller J, Wingren G, et al. Prevalence and correlates of insufficient calcium intake in a Swedish population. Public Health Nurs 2007; 24:511-7.

288. Cauley JA, Wu L, Wampler NS, Barnhart JM, Allison M, Chen Z, et al. Clinical risk factors for fractures in multi-ethnic women: the Women's Health Initiative. J Bone Miner Res 2007; 22:1816-26.

289. Center JR, Bliuc D, Nguyen TV, Eisman JA. Risk of subsequent fracture after low-trauma fracture in men and women. Jama 2007; 297:387-94.

290. Choi MJ, Park EJ, Jo HJ. Relationship of nutrient intakes and bone mineral density of elderly women in Daegu, Korea. Nutr Res Pract 2007; 1:328-34.

291. Diez-Perez A, Gonzalez-Macias J, Marin F, Abizanda M, Alvarez R, Gimeno A, et al. Prediction of absolute risk of non-spinal fractures using clinical risk factors and heel quantitative ultrasound. Osteoporos Int 2007; 18:629-39.

292. Fominykh MI, Popov AA, Izmozherova NV, Akimova AV, Tagil'tseva NV, Striukova O. [Osteopenia and osteoporosis of the distal forearm in menopausal females]. Ter Arkh 2007; 79:46-9.

293. Frazao P, Naveira M. [Factors associated with low bone mineral density among white women]. Rev Saude Publica 2007; 41:740-8.

294. Gu W, Rennie KL, Lin X, Wang Y, Yu Z. Differences in bone mineral status between urban and rural Chinese men and women. Bone 2007; 41:393-9.

295. Hamdi Kara I, Aydin S, Gemalmaz A, Akturk Z, Yaman H, Bozdemir N, et al. Habitual tea drinking and bone mineral density in postmenopausal Turkish women: investigation of prevalence of postmenopausal osteoporosis in Turkey (IPPOT Study). Int J Vitam Nutr Res 2007; 77:389-97.

296. Key TJ, Appleby PN, Spencer EA, Roddam AW, Neale RE, Allen NE. Calcium, diet and fracture risk: a prospective study of 1898 incident fractures among 34 696 British women and men. Public Health Nutr 2007; 10:1314-20.

297. Kung AW, Lee KK, Ho AY, Tang G, Luk KD. Ten-year risk of osteoporotic fractures in postmenopausal Chinese women according to clinical risk factors and BMD T-scores: a prospective study. J Bone Miner Res 2007; 22:1080-7.

298. Lewis CE, Ewing SK, Taylor BC, Shikany JM, Fink HA, Ensrud KE, et al. Predictors of non-spine fracture in elderly men: the MrOS study. J Bone Miner Res 2007; 22:211-9.

299. Masoni A, Morosano M, Tomat MF, Pezzotto SM, Sanchez A. [Association between hip fractures and risk factors for osteoporosis. Multivariate analysis]. Medicina (B Aires) 2007; 67:423-8.

300. Napoli N, Thompson J, Civitelli R, Armamento-Villareal RC. Effects of dietary calcium compared with calcium supplements on estrogen metabolism and bone mineral density. Am J Clin Nutr 2007; 85:1428-33.

301. Nguyen ND, Eisman JA, Center JR, Nguyen TV. Risk factors for fracture in nonosteoporotic men and women. J Clin Endocrinol Metab 2007; 92:955-62.

302. Nurzenski MK, Briffa NK, Price RI, Khoo BC, Devine A, Beck TJ, et al. Geometric indices of bone strength are associated with physical activity and dietary calcium intake in healthy older women. J Bone Miner Res 2007; 22:416-24.

303. Saitoglu M, Ardicoglu O, Ozgocmen S, Kamanli A, Kaya A. Osteoporosis risk factors and association with somatotypes in males. Arch Med Res 2007; 38:746-51.

304. Van Geel TACM, Geusens PP, Nagtzaam IF, Van Der Voort DJM, Schreurs CMJR, Rinkens PELM, et al. Risk factors for clinical fractures among postmenopausal women: A 10-year prospective study. Menopause Int 2007; 13:110-5.

305. Varenna M, Binelli L, Casari S, Zucchi F, Sinigaglia L. Effects of dietary calcium intake on body weight and prevalence of osteoporosis in early postmenopausal women. Am J Clin Nutr 2007; 86:639-44.

306. Bianco V, Filippi F, Tassan-Simonat P, Valente I, D'Amico C, Meroni M. Diet assessment and the screening for osteoporosis: a survey in a healthy menopause population. Minerva Ginecol 2008; 60:299-306, -10.

307. Dargent-Molina P, Sabia S, Touvier M, Kesse E, Breart G, Clavel-Chapelon F, et al. Proteins, dietary acid load, and calcium and risk of postmenopausal fractures in the E3N French women prospective study. J Bone Miner Res 2008; 23:1915-22.

308. El Maghraoui A, Mounach A, Gassim S, Ghazi M. Vertebral fracture assessment in healthy men: Prevalence and risk factors. Bone 2008; 43:544-8.

309. Farrin N, Ostadrahimi AR, Mahboob SA, Kolahi S, Ghavami M. Dietary intake and serum bone related chemistry and their correlations in postmenopausal Iranian women. Saudi Med J 2008; 29:1643-8.

310. Keramat A, Patwardhan B, Larijani B, Chopra A, Mithal A, Chakravarty D, et al. The assessment of osteoporosis risk factors in Iranian women compared with Indian women. BMC Musculoskelet Disord 2008; 9:28.

311. Kim J, Lim SY, Kim JH. Nutrient intake risk factors of osteoporosis in postmenopausal women. Asia Pac J Clin Nutr 2008; 17:270-5.

312. Ma F, Lou HP, Pan H, Yu K, Li N, Liu PJ. [Study on the relationship between intake of nutrients and bone density in middle-aged and old people]. Zhonghua Liu Xing Bing Xue Za Zhi 2008; 29:608-10.

313. Meier C, Nguyen TV, Handelsman DJ, Schindler C, Kushnir MM, Rockwood AL, et al. Endogenous sex hormones and incident fracture risk in older men: the Dubbo Osteoporosis Epidemiology Study. Arch Intern Med 2008; 168:47-54.

314. Nieves JW, Barrett-Connor E, Siris ES, Zion M, Barlas S, Chen YT. Calcium and vitamin D intake influence bone mass, but not short-term fracture risk, in Caucasian postmenopausal women from the National Osteoporosis Risk Assessment (NORA) study. Osteoporos Int 2008; 19:673-9.

315. Ozdemir F, Kabayel DD, Ture M. Do dietary calcium intake and hormone replacement therapy affect bone mineral density in women? Trak Univ Tip Fak De 2008; 25:105-9.

316. Pongchaiyakul C, Kosulwat V, Charoenkiatkul S, Chailurkit LO, Rojroongwasinkul N, Rajatanavin R. The association of dietary calcium, bone mineral density and biochemical bone turnover markers in rural Thai women. J Med Assoc Thai 2008; 91:295-302.

317. Uusi-Rasi K, Sievanen H, Pasanen M, Beck TJ, Kannus P. Influence of calcium intake and physical activity on proximal femur bone mass and structure among pre- and postmenopausal women. A 10-year prospective study. Calcif Tissue Int 2008; 82:171-81.

318. Wang S, Lin S, Zhou Y, Wang Z. Social and behavior factors related to aged Chinese women with osteoporosis. Gynecol Endocrinol 2008; 24:538-45.

319. Zhai G, Hart DJ, Valdes AM, Kato BS, Richards JB, Hakim A, et al. Natural history and risk factors for bone loss in postmenopausal Caucasian women: a 15-year follow-up population-based study. Osteoporos Int 2008; 19:1211-7.

320. Atalar E, Aydin G, Keles I, Inal E, Zog G, Arslan A, et al. Factors affecting bone mineral density in men. Rheumatol Int 2009; 29:1025-30.

321. Duncea I, Georgescu C. Low calcium intake is correlated to high prevalence of osteoporosis in Romanian postmenopausal women. Bone 2009; 44:S404.

322. Farrell VA, Harris M, Lohman TG, Going SB, Thomson CA, Weber JL, et al. Comparison between dietary assessment methods for determining associations between nutrient intakes and bone mineral density in postmenopausal women. J Am Diet Assoc 2009; 109:899-904.

323. Hejazi J, Mohtadinia J, Kolahi S, Ebrahimi-Mamaghani M. Nutritional status among postmenopausal osteoporotic women in North West of Iran. Asia Pac J Clin Nutr 2009; 18:48-53.

324. Ho-Pham LT, Nguyen ND, Vu BQ, Pham HN, Nguyen TV. Prevalence and risk factors of radiographic vertebral fracture in postmenopausal Vietnamese women. Bone 2009; 45:213-7.

325. Ho-Pham LT, Nguyen PL, Le TT, Doan TA, Tran NT, Le TA, et al. Veganism, bone mineral density, and body composition: a study in Buddhist nuns. Osteoporos Int 2009; 20:2087-93.

326. Koh WP, Wu AH, Wang R, Ang LW, Heng D, Yuan JM, et al. Gender-specific associations between soy and risk of hip fracture in the Singapore Chinese Health Study. Am J Epidemiol 2009; 170:901-9.

327. Lopes JB, Danilevicius CF, Takayama L, Caparbo VF, Scazufca M, Bonfa E, et al. Vitamin D insufficiency: a risk factor to vertebral fractures in community-dwelling elderly women. Maturitas 2009; 64:218-22.

328. Mavroeidi A, Stewart AD, Reid DM, Macdonald HM. Physical activity and dietary calcium interactions in bone mass in Scottish postmenopausal women. Osteoporos Int 2009; 20:409-16.

329. Nakamura K, Kurahashi N, Ishihara J, Inoue M, Tsugane S, Japan Public Health Centre-based Prospective Study G. Calcium intake and the 10-year incidence of self-reported vertebral fractures in women and men: the Japan Public Health Centre-based Prospective Study. Br J Nutr 2009; 101:285-94.

330. Nawata K, Yamauchi M, Takaoka S, Imaoka M, Kageyama A, Yamaguchi T, et al. Calcium intake is negatively associated with bone turnover independent of PTH in postmenopausal women. Bone 2009; 44:S86-S7.

331. Pinheiro MM, Schuch NJ, Genaro PS, Ciconelli RM, Ferraz MB, Martini LA. Nutrient intakes related to osteoporotic fractures in men and women--the Brazilian Osteoporosis Study (BRAZOS). Nutr J 2009; 8:6.

332. Popov AA, Izmozherova NV, Strunina MV, Teliushchenko MV. [Detection of distal forearm osteopenia and osteoporosis in Perm women aged 50 years or older]. Ter Arkh 2009; 81:52-6.

333. Thomas-John M, Codd MB, Manne S, Watts NB, Mongey AB. Risk factors for the development of osteoporosis and osteoporotic fractures among older men. J Rheumatol 2009; 36:1947-52.

334. Zhong Y, Okoro CA, Balluz LS. Association of total calcium and dietary protein intakes with fracture risk in postmenopausal women: the 1999-2002 National Health and Nutrition Examination Survey (NHANES). Nutrition 2009; 25:647-54.

335. Cauley JA, Blackwell T, Zmuda JM, Fullman RL, Ensrud KE, Stone KL, et al. Correlates of trabecular and cortical volumetric bone mineral density at the femoral neck and lumbar spine: The osteoporotic fractures in men study (MrOS). J Bone Miner Res 2010; 25:1958-71.

336. Chee WS, Jr., Chong PN, Chuah KA, Karupaiah T, Mustafa N, Suniza S, et al. Calcium Intake, Vitamin D and Bone Health Status of Post-menopausal Chinese Women in Kuala Lumpur. Malays J Nutr 2010; 16:233-42.

337. Fardellone P, Cotte FE, Roux C, Lespessailles E, Mercier F, Gaudin AF. Calcium intake and the risk of osteoporosis and fractures in French women. Joint Bone Spine 2010; 77:154-8.

338. Gronskag AB, Forsmo S, Romundstad P, Langhammer A, Schei B. Dairy products and hip fracture risk among elderly women in Norway - The hunt study. Osteoporos Int 2010; 21:S94-S5.

339. Jha RM, Mithal A, Malhotra N, Brown EM. Pilot case-control investigation of risk factors for hip fractures in the urban Indian population. BMC Musculoskelet Disord 2010; 11:49.

340. Lan TY, Hou SM, Chen CY, Chang WC, Lin J, Lin CC, et al. Risk factors for hip fracture in older adults: a case-control study in Taiwan. Osteoporos Int 2010; 21:773-84.

341. Sahni S, Cupples LA, McLean RR, Tucker KL, Broe KE, Kiel DP, et al. Protective effect of high protein and calcium intake on the risk of hip fracture in the Framingham offspring cohort.[Erratum appears in J Bone Miner Res. 2011 Feb;26(2):439]. J Bone Miner Res 2010; 25:2770-6.

342. Shin A, Lim S, Sung J, Myung S, Kim J. Dietary habit and bone mineral density in Korean postmenopausal women. Osteoporos Int 2010; 21:947-55.

343. Shin CS, Choi HJ, Kim MJ, Kim JT, Yu SH, Koo BK, et al. Prevalence and risk factors of osteoporosis in Korea: a community-based cohort study with lumbar spine and hip bone mineral density. Bone 2010; 47:378-87.

344. Shubeska-Stratrova S, Jovcevska Mecevska J, Radivojevic V, Kocevska A, Kocevska B. Bone turnover markers, hip and spine bone mineral density in postmenopausal women with deficient calcium intake. Osteoporos Int 2010; 21:S301.

345. Aggarwal N, Raveendran A, Khandelwal N, Sen RK, Thakur JS, Dhaliwal LK, et al. Prevalence and related risk factors of osteoporosis in peri- and postmenopausal Indian women. J Midlife Health 2011; 2:81-5.

346. Alissa EM, Qadi SG, Alhujaili NA, Alshehri AM, Ferns GA. Effect of diet and lifestyle factors on bone health in postmenopausal women. J Bone Miner Metab 2011; 29:725-35.

347. Benetou V, Orfanos P, Zylis D, Sieri S, Contiero P, Tumino R, et al. Diet and hip fractures among elderly Europeans in the EPIC cohort. Eur J Clin Nutr 2011; 65:132-9.

348. Chan R, Woo J, Leung J. Effects of food groups and dietary nutrients on bone loss in elderly Chinese population. J Nutr Health Aging 2011; 15:287-94.

349. Fairweather-Tait SJ, Skinner J, Guile GR, Cassidy A, Spector TD, MacGregor AJ. Diet and bone mineral density study in postmenopausal women from the TwinsUK registry shows a negative association with a traditional English dietary pattern and a positive association with wine. Am J Clin Nutr 2011; 94:1371-5.

350. Khoo CC, Woo J, Leung PC, Kwok A, Kwok T. Determinants of bone mineral density in older postmenopausal Chinese women. Climacteric 2011; 14:378-83.

351. Kotsalou I, Valsamaki P, Chatzipetrou A, Gerali S. Dietary calcium intake and bone density in middle-aged white men. Eur J Nucl Med Mol Imaging 2011; 38:S417.

352. Nakamura K, Saito T, Oyama M, Oshiki R, Kobayashi R, Nishiwaki T, et al. Vitamin D sufficiency is associated with low incidence of limb and vertebral fractures in community-dwelling elderly Japanese women: the Muramatsu Study. Osteoporos Int 2011; 22:97-103.

353. Ostertag A, Cohen-Solal M, Madec Y, Baudoin C, de Vernejoul MC. Bone changes in spouses having shared lifestyle for 40 years. Joint Bone Spine 2011; 78:285-90.

354. Park HM, Heo J, Park Y. Calcium from plant sources is beneficial to lowering the risk of osteoporosis in postmenopausal Korean women. Nutr Res 2011; 31:27-32.

355. Perez Durillo FT, Torio Durantez J, Villarejo Villar AB, Sanchez Vico AB, Cueto Camarero Mdel M, Durillo JP. [Comparative study of dietary intake and nutritional status in elderly women with and without hip fracture]. Aten Primaria 2011; 43:362-8.

356. Schwetz V, Walter-Finell D, Bubalo V, Wehr E, Pieber T, Obermayer-Pietsch B. Predictors of bone mineral density and 25(OH)D levels in 99 mothers and daughters. Austrian J Clin Endocrinol Metab 2011; 4:17-8.

357. Simoes V, Mascarenhas MR, Barbosa AP, Oliveira A, Bicho M, Do Carmo I. Calcium intake from milk and yogurt consumption and bone mineral density in portuguese women with osteoporotic fractures. Osteoporos Int 2011; 22:S366-S7.

358. Tsang SWY, Bow CH, Chu EYW, Yeung SC, Soong CC, Kung AWC. Clinical risk factor assessment had better discriminative ability than bone mineral density in identifying subjects with vertebral fracture. Osteoporos Int 2011; 22:667-74.

359. Warensjo E, Byberg L, Melhus H, Gedeborg R, Mallmin H, Wolk A, et al. Dietary calcium intake and risk of fracture and osteoporosis: prospective longitudinal cohort study. BMJ 2011; 342:d1473.

360. Yazdani S, Salemi A, Iranpour Asli A, Heidarnia MA, Sarbakhsh P. Determination of clinical decision rule for estimation of bone mineral density in women. Med Princ Pract 2011; 20:416-21.

361. Anderson JJ, Roggenkamp KJ, Suchindran CM. Calcium intakes and femoral and lumbar bone density of elderly U.S. men and women: National Health and Nutrition Examination Survey 2005-2006 analysis. J Clin Endocrinol Metab 2012; 97:4531-9.

362. Arabi A, Baddoura R, El-Rassi R, El-Hajj Fuleihan G. PTH level but not 25 (OH) vitamin D level predicts bone loss rates in the elderly. Osteoporos Int 2012; 23:971-80.

363. Connie LH, Yu-cho W, Elaine LL, Joanne LK, Kathryn TC. Risk factors related to chinese population with fracture. Osteoporos Int 2012; 1):S786.

364. Khan B, English D, Nowson C, Daly R, Ebeling P. Associations of long-term dietary calcium intake with fractures, cardiovascular events and aortic calcification in a population-based, prospective cohort study. J Bone Miner Res 2012; 27.

365. Martinez-Ramirez MJ, Delgado-Martinez AD, Ruiz-Bailen M, de la Fuente C, Martinez-Gonzalez MA, Delgado-Rodriguez M. Protein intake and fracture risk in elderly people: a case-control study. Clin Nutr 2012; 31:391-5.

366. Marwaha RK, Tandon N, Gupta Y, Bhadra K, Narang A, Mani K, et al. The prevalence of and risk factors for radiographic vertebral fractures in older Indian women and men: Delhi Vertebral Osteoporosis Study (DeVOS). Arch Osteoporos 2012; 7:201-7.

367. Mihalcea S, Gasparik I, Farcas DM. Effect of healthy lifestyle on BMD values. Osteoporos Int 2012; 23:S343.

368. Nakamura K, Oyama M, Saito T, Oshiki R, Kobayashi R, Nishiwaki T, et al. Nutritional and biochemical parameters associated with 6-year change in bone mineral density in community-dwelling Japanese women aged 69 years and older: The Muramatsu Study. Nutrition 2012; 28:357-61.

369. Rouzi AA, Al-Sibiani SA, Al-Senani NS, Radaddi RM, Ardawi MS. Independent predictors of all osteoporosis-related fractures among healthy Saudi postmenopausal women: the CEOR Study. Bone 2012; 50:713-22.

370. Sarkis KS, Martini LA, Szejnfeld VL, Pinheiro MM. Low fatness, reduced fat intake and adequate plasmatic concentrations of LDL-cholesterol are associated with high bone mineral density in women: a cross-sectional study with control group. Lipids Health Dis 2012; 11:37.

371. Thomas SDC, Morris HA. Calcium intake predicts bone loss at the distal forearm in amublatory postmen opausal women. Osteoporos Int 2012; 23:S128.

372. Castro-Lionard K, Dargent-Molina P, Fermanian C, Gonthier R, Cassou B. Use of calcium supplements, vitamin D supplements and specific osteoporosis drugs among French women aged 75-85 years: patterns of use and associated factors. Drugs Aging 2013; 30:1029-38.

373. Chatterjee S, Pedrique G, Thomas S, Sweeney AT. Calcium and vitamin D intake in women with low bone mass referred to an endocrine clinic for bone health evaluation. Endocr Pract 2013; 19:558-9.

374. Ebeling P, English D, Nowson C, Daly R, Khan B. Long term effects of higher dietary calcium intake on vertebral fractures and severe abdominal aortic calcification in older Australians. J Bone Miner Res 2013; 28.

375. Feart C, Lorrain S, Ginder Coupez V, Samieri C, Letenneur L, Paineau D, et al. Adherence to a Mediterranean diet and risk of fractures in French older persons. Osteoporos Int 2013; 24:3031-41.

376. Gonnelli S, Caffarelli C, Tanzilli L, Alessi C, Tomai Pitinca MD, Rossi S, et al. The associations of body composition and fat distribution with bone mineral density in elderly Italian men and women. J Clin Densitom 2013; 16:168-77.

377. Jakobsen A, Laurberg P, Vestergaard P, Andersen S. Clinical risk factors for osteoporosis are common among elderly people in Nuuk, Greenland. Int J Circumpolar Health 2013; 72:19596.

378. Kwok AWL, Gong JS, Wang YXJ, Leung JCS, Kwok T, Griffith JF, et al. Prevalence and risk factors of radiographic vertebral fractures in elderly Chinese men and women: Results of Mr. OS (Hong Kong) and Ms. OS (Hong Kong) studies. Osteoporos Int 2013; 24:877-85.

379. Onat SS, Delialioglu SU, Ozel S. The relationship between osteoporotic risk factors and bone mineral density. [Turkish]. Turk Osteoporoz Dergisi 2013; 19:74-80.

380. Quesada-Gomez JM, Diaz-Curiel M, Sosa-Henriquez M, Malouf-Sierra J, Nogues-Solan X, Gomez-Alonso C, et al. Low calcium intake and inadequate vitamin D status in postmenopausal osteoporotic women. J Steroid Biochem Mol Biol 2013; 136:175-7.

381. Sahni S, Tucker KL, Kiel DP, Quach L, Casey VA, Hannan MT. Milk and yogurt consumption are linked with higher bone mineral density but not with hip fracture: the Framingham Offspring Study. Arch Osteoporos 2013; 8:119.

382. Samieri C, Ginder Coupez V, Lorrain S, Letenneur L, Alles B, Feart C, et al. Nutrient patterns and risk of fracture in older subjects: results from the Three-City Study. Osteoporos Int 2013; 24:1295-305.

383. Speer G, Szamosujvari Jr P, Dombai P, Csore K, Mikofalvi K, Steindl T, et al. Dietary calcium intake and calcium supplementation in hungarian patients with osteoporosis. Int J Endocrinol 2013; 2013.

384. Tajik E, Ebrahimi F, Rasouli B. Bone mineral density contributors, body mass index and calcium intake in postmenopausal women. J Med Sci (Faisalabad) 2013; 13:684-91.

385. Tseng WJ, Hung LW, Shieh JS, Abbod MF, Lin J. Hip fracture risk assessment: artificial neural network outperforms conditional logistic regression in an age- and sex-matched case control study. BMC Musculoskelet Disord 2013; 14:207.

386. Vyskocil V, Pavelka T. Vitamin d levels and calcium supplementation one-year follow up in osteopenic patients in west bohemia. J Bone Miner Res 2013; 28.

387. Zhou W, Langsetmo L, Berger C, Poliquin S, Kreiger N, Barr SI, et al. Longitudinal changes in calcium and vitamin D intakes and relationship to bone mineral density in a prospective population-based study: the Canadian Multicentre Osteoporosis Study (CaMos). J Musculoskelet Neuronal Interact 2013; 13:470-9.

388. Alissa EM, Alnahdi WA, Alama N, Ferns GA. Relationship between nutritional profile, measures of adiposity, and bone mineral density in postmenopausal saudi women. J Am Coll Nutr 2014; 33:206-14.

389. Betancourt Ortiz SL. [Bone mineral density, dietary calcium and risk factor for presumptive osteoporosis in ecuadorian aged women]. Nutr Hosp 2014; 30:372-84.

390. Borges CN, de Almeida JM, Lima D, Cabral M, Bandeira F. Prevalence of morphometric vertebral fractures in old men and the agreement between different methods in the city of Recife, Brazil. Rheumatol Int 2014.

391. Domiciano DS, Machado LG, Lopes JB, Figueiredo CP, Caparbo VF, Takayama L, et al. Incidence and risk factors for osteoporotic vertebral fracture in low-income community-dwelling elderly: a population-based prospective cohort study in Brazil. The Sao Paulo Ageing & Health (SPAH) Study. Osteoporos Int 2014.

392. Gunn CA, Weber JL, Kruger MC. Diet, weight, cytokines and bone health in postmenopausal women. J Nutr Health Aging 2014; 18:479-86.

393. Hassan R, Rahman MM, Arefin U, Yusuf MA, Barua S, Moniruzaman M, et al. Risk factors for osteoporotic fracture: A case control study. Int J Rheum Dis 2014; 17:84-5.

394. Kim KC, Chun H, Lai C, Parnell LD, Jang Y, Lee J, et al. The association between genetic variants of RUNX2, ADIPOQ and vertebral fracture in Korean postmenopausal women. J Bone Miner Metab 2014.

395. Kim KM, Choi SH, Lim S, Moon JH, Kim JH, Kim SW, et al. Interactions Between Dietary Calcium Intake and Bone Mineral Density or Bone Geometry in a Low Calcium Intake Population (KNHANES IV 2008-2010). J Clin Endocrinol Metab 2014; 99:2409-17.

396. Paknahad Z, Mohammadifard N, Bonakdar Z, Hasanzadeh A. Nutritional status and its relationship with bone mass density in postmenopausal women admitted in osteodensitometry center, Isfahan-Iran. J Educ Health Promot 2014; 3:48.

397. Radavelli-Bagatini S, Zhu K, Lewis JR, Prince RL. Dairy food intake, peripheral bone structure, and muscle mass in elderly ambulatory women. J Bone Miner Res 2014; 29:1691-700.

398. Sahni S, Mangano KM, Tucker KL, Kiel DP, Casey VA, Hannan MT. Protective association of milk intake on the risk of hip fracture: results from the framingham original cohort. J Bone Miner Res 2014; 29:1756-62.

399. Shankar M. Correlation of BMD and fractures with dietary intake of calcium : A brief analysis of collective datawithin a metropolitan hospital in South Bangalore. Osteoporos Int 2014; 25:S294.

400. Skowronska-Jozwiak E, Jaworski M, Grzywa A, Lorenc R, Lewinski A. Influence of calcium intake on bone mineral density and incidence of fractures in treatment-naive women from Lodz urban area - a part of EPOLOS study. Ann Agric Environ Med 2014; 21:201-4.

401. van den Berg P, van Haard PM, van den Bergh JP, Niesten DD, van der Elst M, Schweitzer DH. First quantification of calcium intake from calcium-dense dairy products in Dutch fracture patients (the Delft cohort study). Nutrients 2014; 6:2404-18.

402. Wlodarek D, Glabska D, Kolota A, Adamczyk P, Czekajlo A, Grzeszczak W, et al. Calcium intake and osteoporosis: the influence of calcium intake from dairy products on hip bone mineral density and fracture incidence - a population-based study in women over 55 years of age. Public Health Nutr 2014; 17:383-9.

403. Christiansen C, Rodbro P, Lund M. Incidence of anticonvulsant osteomalacia and effect of vitamin D: controlled therapeutic trial. Br Med J 1973; 4:695-701.

404. Alhava EM, Aukee S, Karjalainen P, Kettunen K, Juuti M. The influence of calcium and calcium + vitamin D2 treatment on bone mineral after partial gastrectomy. Scand J Gastroenterol 1975; 10:689-93.

405. Christiansen C, Christensen MS, McNair P, Hagen C, Stocklund KE, Transbol I. Prevention of early postmenopausal bone loss: controlled 2-year study in 315 normal females. Eur J Clin Invest 1980; 10:273-9.

406. Takizawa H, Igarashi M, Hayashi Y, Karube S, Kimura H. [Comparison of treatments in senile osteoporosis: follow up for 12 months (author's transl)]. Nihon Seikeigeka Gakkai Zasshi 1980; 54:345-55.

407. Mobarhan SA, Russell RM, Recker RR, Posner DB, Iber FL, Miller P. Metabolic bone disease in alcoholic cirrhosis: a comparison of the effect of vitamin D2, 25-hydroxyvitamin D, or supportive treatment. Hepatology 1984; 4:266-73.

408. Dawson-Hughes B, Dallal GE, Krall EA, Harris S, Sokoll LJ, Falconer G. Effect of vitamin D supplementation on wintertime and overall bone loss in healthy postmenopausal women. Ann Intern Med 1991; 115:505-12.

409. Dawson-Hughes B, Harris SS, Krall EA, Dallal GE, Falconer G, Green CL. Rates of bone loss in postmenopausal women randomly assigned to one of two dosages of vitamin D. Am J Clin Nutr 1995; 61:1140-5.

410. Vogelsang H, Ferenci P, Resch H, Kiss A, Gangl A. Prevention of bone mineral loss in patients with Crohn's disease by long-term oral vitamin D supplementation. Eur J Gastroenterol Hepatol 1995; 7:609-14.

411. Adachi JD, Bensen WG, Bianchi F, Cividino A, Pillersdorf S, Sebaldt RJ, et al. Vitamin D and calcium in the prevention of corticosteroid induced osteoporosis: a 3 year followup. J Rheumatol 1996; 23:995-1000.

412. Bernstein CN, Seeger LL, Anton PA, Artinian L, Geffrey S, Goodman W, et al. A randomized, placebo-controlled trial of calcium supplementation for decreased bone density in corticosteroid-using patients with inflammatory bowel disease: a pilot study. Aliment Pharmacol Ther 1996; 10:777-86.

413. Buckley LM, Leib ES, Cartularo KS, Vacek PM, Cooper SM. Calcium and vitamin D3 supplementation prevents bone loss in the spine secondary to low-dose corticosteroids in patients with rheumatoid arthritis. A randomized, double-blind, placebo-controlled trial. Ann Intern Med 1996; 125:961-8.

414. Lips P, Graafmans WC, Ooms ME, Bezemer PD, Bouter LM. Vitamin D supplementation and fracture incidence in elderly persons. A randomized, placebo-controlled clinical trial. Ann Intern Med 1996; 124:400-6.

415. Mautalen C, Gonzalez D, Mazure R, Vazquez H, Lorenzetti MP, Maurino E, et al. Effect of treatment on bone mass, mineral metabolism, and body composition in untreated celiac disease patients. Am J Gastroenterol 1997; 92:313-8.

416. Komulainen MH, Kroger H, Tuppurainen MT, Heikkinen AM, Alhava E, Honkanen R, et al. HRT and Vit D in prevention of non-vertebral fractures in postmenopausal women; a 5 year randomized trial. Maturitas 1998; 31:45-54.

417. Tuppurainen MT, Komulainen M, Kroger H, Honkanen R, Jurvelin J, Puntila E, et al. Does vitamin D strengthen the increase in femoral neck BMD in osteoporotic women treated with estrogen? Osteoporos Int 1998; 8:32-8.

418. Krieg MA, Jacquet AF, Bremgartner M, Cuttelod S, Thiebaud D, Burckhardt P. Effect of supplementation with vitamin D3 and calcium on quantitative ultrasound of bone in elderly institutionalized women: a longitudinal study. Osteoporos Int 1999; 9:483-8.

419. Hunter D, Major P, Arden N, Swaminathan R, Andrew T, MacGregor AJ, et al. A randomized controlled trial of vitamin D supplementation on preventing postmenopausal bone loss and modifying bone metabolism using identical twin pairs. J Bone Miner Res 2000; 15:2276-83.

420. Patel R, Collins D, Bullock S, Swaminathan R, Blake GM, Fogelman I. The effect of season and vitamin D supplementation on bone mineral density in healthy women: a double-masked crossover study. Osteoporos Int 2001; 12:319-25.

421. Meyer HE, Smedshaug GB, Kvaavik E, Falch JA, Tverdal A, Pedersen JI. Can vitamin D supplementation reduce the risk of fracture in the elderly? A randomized controlled trial. J Bone Miner Res 2002; 17:709-15.

422. Cooper L, Clifton-Bligh PB, Nery ML, Figtree G, Twigg S, Hibbert E, et al. Vitamin D supplementation and bone mineral density in early postmenopausal women. Am J Clin Nutr 2003; 77:1324-9.

423. Trivedi DP, Doll R, Khaw KT. Effect of four monthly oral vitamin D3 (cholecalciferol) supplementation on fractures and mortality in men and women living in the community: randomised double blind controlled trial. BMJ 2003; 326:469.

424. Venkatachalam S, Gupta R, Speden D, Fickling W, Robertson D, Ring E, et al. A randomised controlled trial of parenteral vitamin D in coeliac disease-Bone density changes. Osteoporos Int 2003; 14:S39-S40.

425. Di Daniele N, Carbonelli MG, Candeloro N, Iacopino L, De Lorenzo A, Andreoli A. Effect of supplementation of calcium and vitamin D on bone mineral density and bone mineral content in peri- and post-menopause women; a double-blind, randomized, controlled trial. Pharmacol Res 2004; 50:637-41.

426. Goode LR, Brolin RE, Chowdhury HA, Shapses SA. Bone and gastric bypass surgery: effects of dietary calcium and vitamin D. Obes Res 2004; 12:40-7.

427. Haworth CS, Jones AM, Adams JE, Selby PL, Webb AK. Randomised double blind placebo controlled trial investigating the effect of calcium and vitamin D supplementation on bone mineral density and bone metabolism in adult patients with cystic fibrosis. J Cyst Fibros 2004; 3:233-6.

428. Wissing KM, Broeders N, Moreno-Reyes R, Gervy C, Stallenberg B, Abramowicz D. A controlled study of vitamin D3 to prevent bone loss in renal-transplant patients receiving low doses of steroids. Transplantation 2005; 79:108-15.

429. Jackson RD, LaCroix AZ, Gass M, Wallace RB, Robbins J, Lewis CE, et al. Calcium plus vitamin D supplementation and the risk of fractures. N Engl J Med 2006; 354:669-83.

430. Law M, Withers H, Morris J, Anderson F. Vitamin D supplementation and the prevention of fractures and falls: results of a randomised trial in elderly people in residential accommodation. Age Ageing 2006; 35:482-6.

431. Mikati MA, Dib L, Yamout B, Sawaya R, Rahi AC, Fuleihan Gel H. Two randomized vitamin D trials in ambulatory patients on anticonvulsants: impact on bone. Neurology 2006; 67:2005-14.

432. Moschonis G, Manios Y. Skeletal site-dependent response of bone mineral density and quantitative ultrasound parameters following a 12-month dietary intervention using dairy products fortified with calcium and vitamin D: the Postmenopausal Health Study. Br J Nutr 2006; 96:1140-8.

433. Zhang ZL, He JW, Huang QR, Qin YJ, Hu YQ, Li M, et al. [Relationship between the polymorphism of start codon and CDX2 site in vitamin D receptor gene and the effect of calcium supplementation on bone mineral density of postmenopausal women]. Zhonghua Yi Xue Yi Chuan Xue Za Zhi 2006; 23:397-401.

434. Lyons RA, Johansen A, Brophy S, Newcombe RG, Phillips CJ, Lervy B, et al. Preventing fractures among older people living in institutional care: a pragmatic randomised double blind placebo controlled trial of vitamin D supplementation. Osteoporos Int 2007; 18:811-8.

435. Smith H, Anderson F, Raphael H, Maslin P, Crozier S, Cooper C. Effect of annual intramuscular vitamin D on fracture risk in elderly men and women--a population-based, randomized, double-blind, placebo-controlled trial. Rheumatology (Oxford) 2007; 46:1852-7.

436. Andersen R, Molgaard C, Skovgaard LT, Brot C, Cashman KD, Jakobsen J, et al. Effect of vitamin D supplementation on bone and vitamin D status among Pakistani immigrants in Denmark: a randomised double-blinded placebo-controlled intervention study. Br J Nutr 2008; 100:197-207.

437. Lappe J, Cullen D, Haynatzki G, Recker R, Ahlf R, Thompson K. Calcium and vitamin d supplementation decreases incidence of stress fractures in female navy recruits. J Bone Miner Res 2008; 23:741-9.

438. Carlin AM, Rao DS, Yager KM, Parikh NJ, Kapke A. Treatment of vitamin D depletion after Roux-en-Y gastric bypass: a randomized prospective clinical trial. Surg Obes Relat Dis 2009; 5:444-9.

439. Viljakainen HT, Vaisanen M, Kemi V, Rikkonen T, Kroger H, Laitinen EK, et al. Wintertime vitamin D supplementation inhibits seasonal variation of calcitropic hormones and maintains bone turnover in healthy men. J Bone Miner Res 2009; 24:346-52.

440. Islam MZ, Shamim AA, Viljakainen HT, Akhtaruzzaman M, Jehan AH, Khan HU, et al. Effect of vitamin D, calcium and multiple micronutrient supplementation on vitamin D and bone status in Bangladeshi premenopausal garment factory workers with hypovitaminosis D: a double-blinded, randomised, placebo-controlled 1-year intervention. Br J Nutr 2010; 104:241-7.

441. Sanders KM, Stuart AL, Williamson EJ, Simpson JA, Kotowicz MA, Young D, et al. Annual high-dose oral vitamin D and falls and fractures in older women: a randomized controlled trial. JAMA 2010; 303:1815-22.

442. Moschonis G, Kanellakis S, Papaioannou N, Schaafsma A, Manios Y. Possible site-specific effect of an intervention combining nutrition and lifestyle counselling with consumption of fortified dairy products on bone mass: the Postmenopausal Health Study II. J Bone Miner Metab 2011; 29:501-6.

443. Verschueren SM, Bogaerts A, Delecluse C, Claessens AL, Haentjens P, Vanderschueren D, et al. The effects of whole-body vibration training and vitamin D supplementation on muscle strength, muscle mass, and bone density in institutionalized elderly women: a 6-month randomized, controlled trial. J Bone Miner Res 2011; 26:42-9.

444. Grimnes G, Joakimsen R, Figenschau Y, Torjesen PA, Almas B, Jorde R. The effect of high-dose vitamin D on bone mineral density and bone turnover markers in postmenopausal women with low bone mass--a randomized controlled 1-year trial. Osteoporos Int 2012; 23:201-11.

445. Iuliano-Burns S, Ayton J, Hillam S, Jones G, King K, Macleod S, et al. Skeletal and hormonal responses to vitamin D supplementation during sunlight deprivation in Antarctic expeditioners. Osteoporos Int 2012; 23:2461-7.

446. Nieves JW, Cosman F, Grubert E, Ambrose B, Ralston SH, Lindsay R. Skeletal effects of vitamin D supplementation in postmenopausal black women. Calcif Tissue Int 2012; 91:316-24.

447. Bolland MJ, Wilsher ML, Grey A, Horne AM, Fenwick S, Gamble GD, et al. Randomised controlled trial of vitamin D supplementation in sarcoidosis. BMJ Open 2013; 3:e003562.

448. Wamberg L, Pedersen SB, Richelsen B, Rejnmark L. The effect of high-dose vitamin D supplementation on calciotropic hormones and bone mineral density in obese subjects with low levels of circulating 25-hydroxyvitamin d: results from a randomized controlled study. Calcif Tissue Int 2013; 93:69-77.

449. Czech-Kowalska J, Latka-Grot J, Bulsiewicz D, Jaworski M, Pludowski P, Wygledowska G, et al. Impact of vitamin D supplementation during lactation on vitamin D status and body composition of mother-infant pairs: a MAVID randomized controlled trial. PLoS One 2014; 9:e107708.

450. Gaffney-Stomberg E, Lutz LJ, Rood JC, Cable SJ, Pasiakos SM, Young AJ, et al. Calcium and vitamin D supplementation maintains parathyroid hormone and improves bone density during initial military training: a randomized, double-blind, placebo controlled trial. Bone 2014; 68:46-56.

451. Mieczkowski M, Zebrowski P, Wojtaszek E, Stompor T, Przedlacki J, Bartoszewicz Z, et al. Long-term cholecalciferol administration in hemodialysis patients: a single-center randomized pilot study. Med Sci Monit 2014; 20:2228-34.

452. Norenstedt S, Pernow Y, Zedenius J, Nordenstrom J, Saaf M, Granath F, et al. Vitamin D supplementation after parathyroidectomy: effect on bone mineral density-a randomized double-blind study. J Bone Miner Res 2014; 29:960-7.

453. Rolighed L, Rejnmark L, Sikjaer T, Heickendorff L, Vestergaard P, Mosekilde L, et al. Vitamin D treatment in primary hyperparathyroidism: a randomized placebo controlled trial. J Clin Endocrinol Metab 2014; 99:1072-80.

454. Chen Y, Zhang Q, Wang Y, Xiao Y, Fu R, Bao H, et al. Estimating the causal effect of milk powder supplementation on bone mineral density: a randomized controlled trial with both non-compliance and loss to follow-up. Eur J Clin Nutr 2015; 69:824-30.

455. Hansen KE, Johnson RE, Chambers KR, Johnson MG, Lemon CC, Vo TN, et al. Treatment of Vitamin D Insufficiency in Postmenopausal Women: A Randomized Clinical Trial. JAMA Intern Med 2015; 175:1612-21.

456. Overton ET, Chan ES, Brown TT, Tebas P, McComsey GA, Melbourne KM, et al. Vitamin D and Calcium Attenuate Bone Loss With Antiretroviral Therapy Initiation: A Randomized Trial. Ann Intern Med 2015; 162:815-24.

457. Rousseau AF, Foidart-Desalle M, Ledoux D, Remy C, Croisier JL, Damas P, et al. Effects of cholecalciferol supplementation and optimized calcium intakes on vitamin D status, muscle strength and bone health: a one-year pilot randomized controlled trial in adults with severe burns. Burns 2015; 41:317-25.

458. Silk LN, Greene DA, Baker MK, Jander CB. Tibial bone responses to 6-month calcium and vitamin D supplementation in young male jockeys: A randomised controlled trial. Bone 2015; 81:554-61.
